# Photoactivated Cyclic Polyphthalaldehyde Microcapsules for Payload Delivery

**DOI:** 10.1021/acsami.4c07609

**Published:** 2024-08-07

**Authors:** Youngsu Shin, Jared M. Schwartz, Anthony C. Engler, Brad Jones, Oleg Davydovich, Paul A. Kohl

**Affiliations:** † School of Chemical and Biomolecular Engineering, Georgia Institute of Technology, Atlanta, Georgia 30332, United States; ‡ Cain Department of Chemical Engineering, Louisiana State University, Baton Rouge, Louisiana 70803, United States; § Sandia National Laboratories, Albuquerque, New Mexico 87185, United States

**Keywords:** stimuli-responsive, phototriggerable, microcapsule, metastable polymer, transient polymer, polyphthalaldehyde, emulsification, photoacid generator

## Abstract

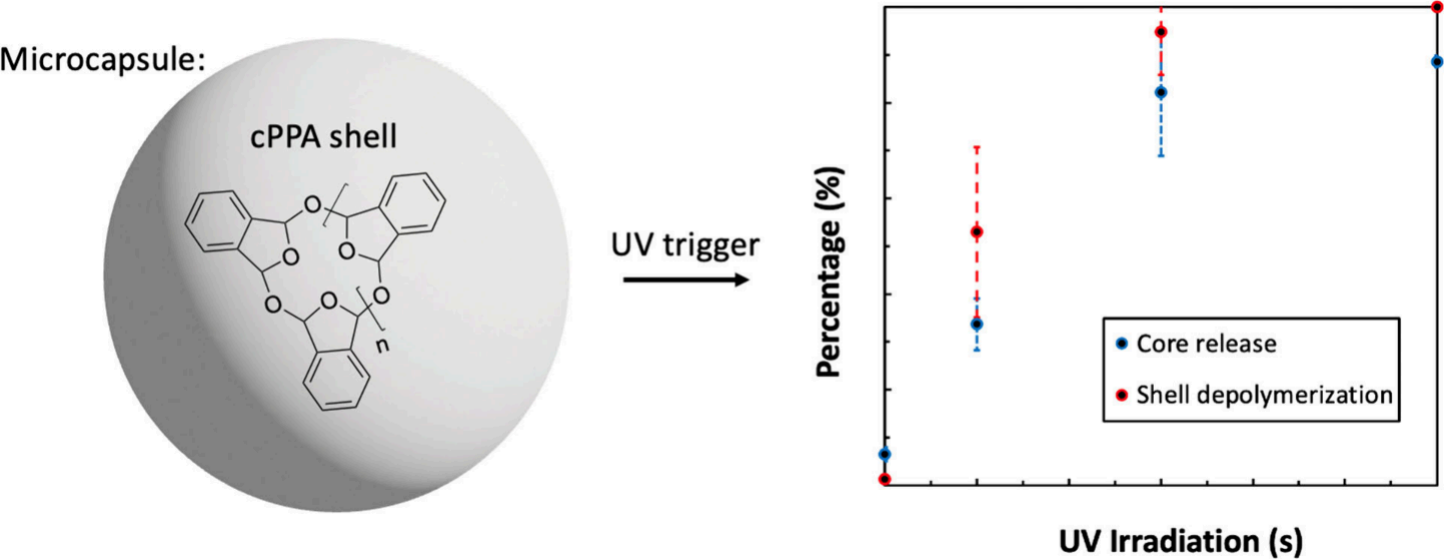

Microcapsules with a cyclic polyphthalaldehyde (cPPA) shell and oil core were fabricated by an emulsification process. The low ceiling temperature cPPA shell was made phototriggerable by incorporating a photoacid generator (PAG). Photoactivation of the PAG created a strong acid which catalyzed cPPA depolymerization, resulting in the release of the core payload, as quantified by ^1^H NMR. The high molecular weight cPPA (197 kDa) yielded uniform spherical microcapsules. The core diameter was 24.8 times greater than the cPPA shell thickness (2.4 to 21.6 μm). Nonionic bis(cyclohexylsulfonyl)diazomethane (BCSD) and *N*-hydroxynaphthalimide triflate (HNT) PAGs were used as the PAG in the microcapsule shells. BCSD required dual stimuli of UV radiation and post-exposure baking at 60 °C to activate cPPA depolymerization while room temperature irradiation of HNT resulted in instantaneous core release. A 300 s UV exposure (365 nm, 10.8 J/cm^2^) of the cPPA/HNT microcapsules resulted in 66.5 ± 9.4% core release. Faster core release was achieved by replacing cPPA with a phthalaldehyde/propanal copolymer. A 30 s UV exposure (365 nm, 1.08 J/cm^2^) resulted in 82 ± 13% core release for the 75 mol % phthalaldehyde/25 mol % propanal copolymer microcapsules. The photoresponsive shell provides a versatile polymer microcapsule technology for on-demand, controlled release of hydrophobic core payloads.

## Introduction

The triggered release of core/shell microcapsules is a possible approach to the controlled release of active core materials. Microcapsules have been used in many applications such as drug delivery,^1,2^ self-healing materials,^3,4^ marine antifoul coatings,^5,6^ food additives,^7,8^ and chemical recycling.^9^ Stimuli-responsive polymers are a particularly useful microcapsule shell material because their decomposition can be triggered by a pH change,^10−12^ elevated temperature,^13^ or ultraviolet (UV) exposure^14−17^ to release the core. Electromagnetic radiation triggers, especially visible and UV radiation, are often selected because they are convenient to use without direct physical contact with the microcapsule, and the activation wavelength is tunable.

TiO_2_^15−17^ and gold^18^ nanoparticles have been embedded in a polymeric shell for photoactivated depolymerization by using a layer-by-layer polyelectrolyte assembly process or by covering the polymer shell with a thin TiO_2_ layer.^19^ However, the quantum efficiency of TiO_2_ is limited by the fast recombination of photogenerated charge carriers, and the catalytic activity is hindered by TiO_2_ aggregation. Also, the TiO_2_ separation, recovery, and reuse are challenging.^20−22^ Graphene oxide^23^ has been used to make photoreactive microcapsules via a microfluidic system. Both the layer-by-layer and microfluidic techniques to form the microcapsule shell with nanoparticles are intricate, time-intensive, and challenging to scale. Alternatively, an emulsification and solvent evaporation with a metastable polymer shell are efficient methods to make microcapsules in bulk. The use of a low ceiling temperature (*T*_C_) polymer as the shell is attractive because it is metastable above its *T*_C_ due to the thermodynamic instability. For example, polyphthalaldehyde (PPA) has a *T*_C_ of −36 °C.^24,25^ PPA is metastable at room temperature by either end-capping the linear form (lPPA) or synthesizing the cyclic form (cPPA) to remove the active chain ends. Depolymerization of cPPA causes the polymer to spontaneously revert back to its monomer, *o*-phthalaldehyde (*o*PA). cPPA depolymerization can be triggered by elevated temperature,^26,27^ acid,^28,29^ single electron transfer,^30^ or mechanical force.^31^

PPA has been used in the microcapsule shell because the polymer can be easily depolymerized to release the core. DiLauro et al. produced end-capped lPPA microcapsules with reactive groups to initiate lPPA depolymerization.^32,33^ Tang et al. produced microcapsules with a cPPA shell and jojoba oil core by using an emulsification and solvent evaporation method. They studied the microcapsule morphology and thermal stability, the core release mechanism by acid gas exposure, and the ion coactivation effect.^34,35^ Eriksson et al. produced microcapsules with hexadecane oil encapsulated in an lPPA shell and studied the core release by direct UV radiation.^36^ Their results are not congruent with other reports^36^ of PPA thin films in UV systems. lPPA and cPPA polymers have been used for making microcapsules. Only cPPA was chosen as the shell material in this study for its superior chemical and thermal stability. DiLauro et al.^33^ showed that lPPA gradually depolymerized at room temperature at a rate of 1% to 3% per day due to inherent instability of end-caps, while Schwartz et al.^37^ observed that cPPA depolymerized only 1% after 21 days at accelerated aging conditions (40 °C). It was speculated that the purified cPPA would remain stable for 13 years at 20 °C.^37^

Photoacid generators (PAG) have been used to catalyze PPA depolymerization.^38−43^ The activated PAG attacks the acetal linkage in the PPA chain and leads to a cascading depolymerization reaction.^28,29^ However, PPA microcapsules using photoactivated PAG have not been investigated. Koo et al.^14^ reported polyelectrolyte multilayer microcapsules with PAG, but their core release occurred by inducing a pH change. Photoactivated microcapsules with a metastable polymer may expand the applications of transient polymers in controlled release.

In this report, phototriggerable microcapsules with a cPPA/PAG shell and oil core were fabricated, using an emulsification and solvent evaporation method. Nonionic PAGs, bis(cyclohexylsulfonyl)diazomethane (BCSD) and *N*-hydroxynaphthalimide triflate (HNT), and an ionic PAG, Rhodorsil FABA, were investigated. These PAGs were chosen for study here because other PAGs interfered with microcapsule formation.

**Figure 1:**
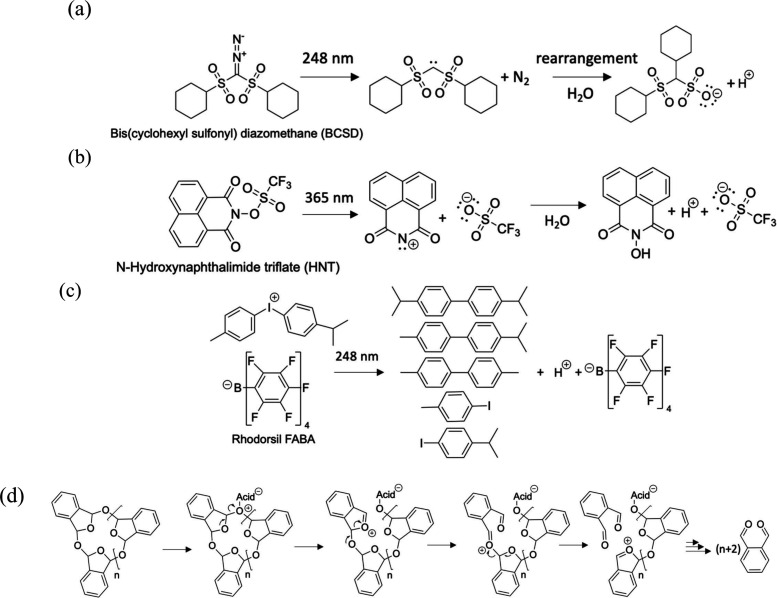
Molecular structure of (a) BCSD,^45^ (b) HNT,^47^ and (c) Rhodorsil FABA^44^ and their respective photoactivated products are shown. The strong acid produced from photoactivated PAGs leads to cPPA depolymerization by breaking the acetal linkage,^28,29^ as illustrated in (d).

The UV-activated PAGs catalyzed the depolymerization of the cPPA shell after exposure.^38−43^ UV-activated Rhodorsil FABA creates a strong Bronsted acid with a p*K*_a_ between −13 and −15 due to its highly delocalized structure and abundant fluorine content.^44^ A strong acid is generated by BCSD^45^ and Rhodorsil FABA^46^ at 248 nm and by HNT^47^ at 365 nm radiation. Overall, core release rates were tuned by the different types of PAGs, UV exposure time, and post-exposure thermal treatment. A schematic diagram of the activated PAGs under UV radiation and cPPA depolymerization is shown in Figure 1.

## Experimental Methods

All chemicals were purchased from VWR unless otherwise specified. Low molecular weight (MW) cPPA (*M*_n_ = 30 to 80 kDa with 1.4 to 2.2 dispersity (*Đ*)), high MW cPPA (*M*_n_ = 196.66 kDa with 2.7 *Đ*), and cyclic poly(phthalaldehyde-*co*-propanal) with 75 mol % phthalaldehyde and 25 mol % propanal (*M*_n_ = 19.5 kDa with 2.3 *Đ*) were synthesized by cationic polymerization of *o*-phthalaldehyde (*o*PA) and precipitated, following the procedures described in the literature.^37,48^ The resulting 75 mol % PPA/25 mol % PA copolymer has a random monomer distribution. All homo- and copolymers used in this study have a cyclic architecture, synthesized by a trans-acetalization reaction with boron trifluoride diethyl etherate catalyst with the synthesis at −78 °C in anhydrous dichloromethane (DCM). Pyridine with 67 mol excess to the catalyst was added to quench the polymerization. The resulting solution was precipitated into vigorously stirred methanol. The white solid polymer was air-dried overnight and stored at −85 °C before usage.^37,48^ The number-average molecular weight (*M*_n_) and dispersity index (*Đ*) were determined by GPC (Shimadzu with a LC-20 AD HPLC pump and a refractive index detector, RID-20 A, 120 V). The GPC sample was prepared in tetrahydrofuran with an eluent flow rate of 1.0 mL min^–1^ with polystyrene standard.

Poly(vinyl alcohol) (PVA), *M*_w_ = 9 to 10 kDa and 80% hydrolyzed, was used as a surfactant in the emulsion experiments. BCSD, Rhodorsil FABA, and HNT were purchased from BOC Sciences, TCI Chemicals, and Sigma-Aldrich, respectively.

A Caframo overhead stirrer was used for emulsification. The UV source used was a 1000 W Hg(Xe) Oriel Instruments flood exposure lamp with a 248 or 365 nm bandpass filter, depending on the wavelengths of interest. The thermal stability of the microcapsules was measured using thermogravimetric analysis (TGA) using a Q50 tool (TA Instruments) under a nitrogen atmosphere with a constant flow rate of 40 mL min^–1^. The furnace temperature was ramped up at 30 °C min^–1^ to 60 °C and held isothermally for 500 min. Optical images for microcapsules were obtained with an Olympus SZX-STAD2 microscope. Nuclear magnetic resonance (NMR) spectra were recorded using a Bruker Avance III 400 MHz instrument. Chemical shifts are reported in δ (ppm) relative to the residual solvent peak from CDCl_3_ (δ = 7.26 ppm for ^1^H).

Emulsion experiments were performed in a clean room with filters blocking UV radiation. Microcapsules were prepared by the internal phase separation method from Loxley et al.^49^ and Eriksson et al.^50^ The oil phase was a homogeneous solution consisting of 0.66 g of cPPA, 0.5 g of dodecane (DD), 0.5 mL of acetone, and PAG (specified as a wt % with respect to cPPA) in 5 mL of DCM. This solution was added dropwise to a mixture consisting of 20 mL of deionized (DI) water with 1 wt % PVA stirred at 1000 rpm. After adding the oil phase, the stirring was increased to 2500 rpm. After emulsification, the mixture was transferred to a 25 mL round-bottom (RB) flask containing 30 mL of DI water. The solution was vigorously stirred in an open RB flask using a magnetic stir bar to promote DCM evaporation in a chemical hood (DCM evaporation step). Powdery and dry microcapsules were obtained by vacuum filtration using Whatman 2 filter paper. The microcapsules were dried at 60 °C for roughly 4 h to remove residual solvent. The microcapsules were stored in a refrigerator at 3 °C. The size variance of microcapsules (i.e., diameter of microcapsules) using 200 microcapsules taken with an Olympus SZX-STAD2 microscope with 5× Olympus objective was measured using ImageJ software.

Microcapsules (8 to 12 mg) were placed as a single layer in a 20 mL scintillation vial or quartz glassware and were exposed to UV radiation. After UV exposure, the microcapsules with BCSD were heated in an oven at 60 °C. The microcapsules with HNT did not have a post-exposure thermal treatment. The microcapsules were washed with 4 mL of diethyl ether for 1 min, which acts as a solvent for the DD and *o*PA monomer but a nonsolvent for the cPPA. A 1 mL aliquot of the solution was retained, and the remainder was discarded. The diethyl ether was evaporated by flowing dry nitrogen gas over the sample. The residuals were analyzed by ^1^H NMR to quantify the DD released from the capsules and the cPPA depolymerization through use of an internal standard, dimethylformamide (DMF). The washed microcapsules were separately analyzed by ^1^H NMR to quantify the cPPA and DD remaining within the microcapsules. Microcapsules were either covered by aluminum foil or processed in a clean room to prevent inadvertent UV exposure.

## Results and Discussion

**Figure 2:**
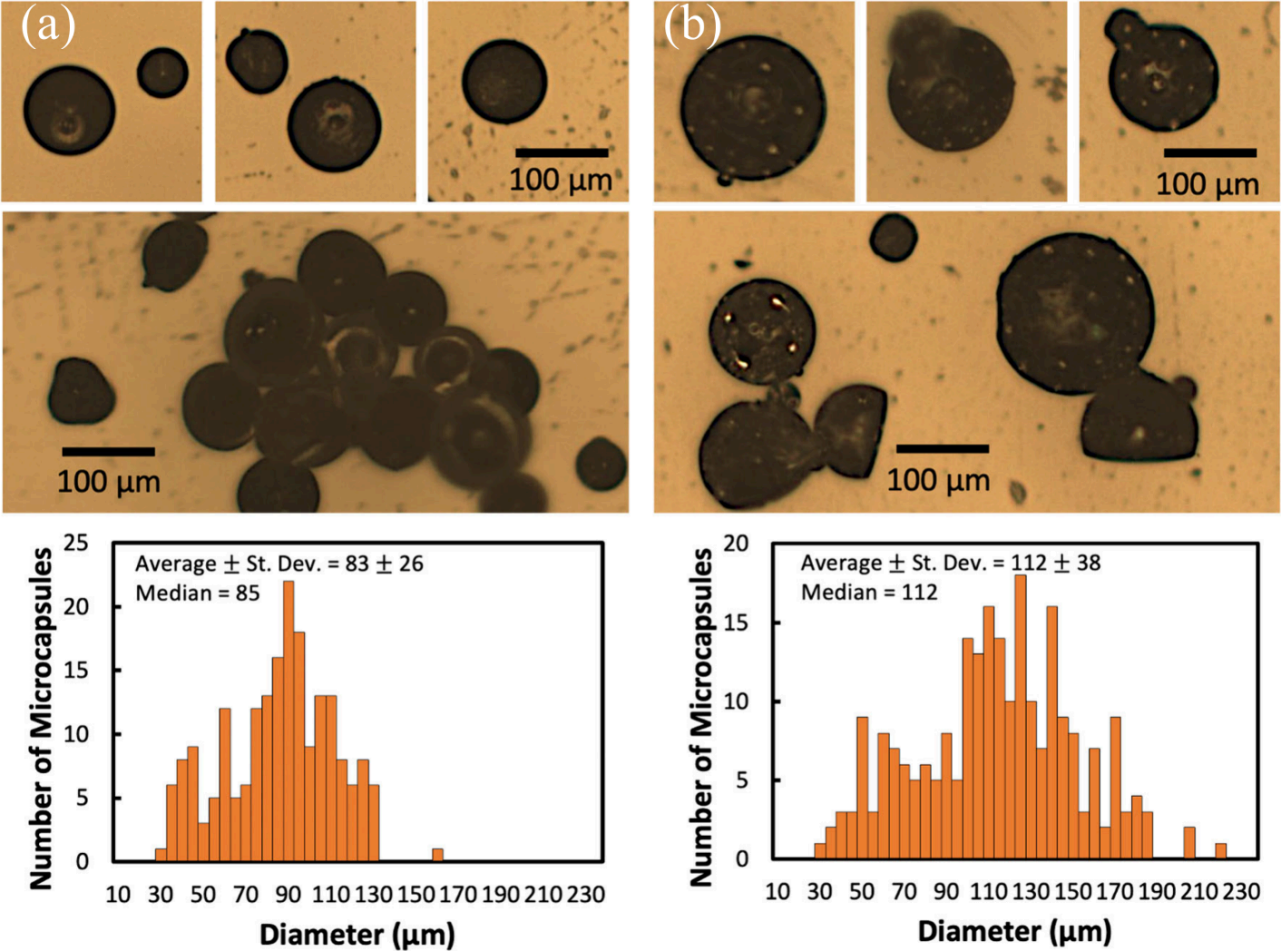
Characterization of low MW cPPA/DD microcapsules with 5 wt % BCSD PAG with (a) shorter evaporation time (60 to 80 min) and (b) longer evaporation time (overnight) after emulsification is shown. Both (a) and (b) showed blueberry-shaped morphology (1st row) and concave morphology (2nd row). Only (b) displayed a rough surface with small dimples. The size variances of the microcapsules are shown with their average standard deviation (St. Dev.) and median.

The emulsion procedure with low MW cPPA and DD produced blueberry-shaped and concave-shaped microcapsules. (Note: the terminology used here to describe the microcapsule shapes comes from that used in previous publications.^34,49,50^) The capsule surfaces became visibly rougher with small dimples when the DCM evaporation time after emulsification was increased from (a) 60 to 80 min to (b) overnight in Figure 2. Images of microcapsules containing BCSD, along with their respective size variance, are also shown. The addition of Rhodorsil FABA to the shell did not change the microcapsule size or morphology (between 30 and 200 μm (Figure S1)).

In a similar study, Eriksson et al.^50^ used the same slow evaporation method with a hexadecane core and an lPPA shell to obtain spherical, acorn-shaped, and blueberry-shaped structures in the solution phase. The same morphologies were observed in this study. Blueberry-shaped and spherical microcapsules were observed, and both had a high core loading. Concave-shaped microcapsules (head of acorn-shaped structures in the solution phase) with a low core loading were also observed. The oil core was most likely present outside of the concave-shaped structures. The oil was removed during vacuum filtration to obtain dry and powdery microcapsules.

**Figure 3:**
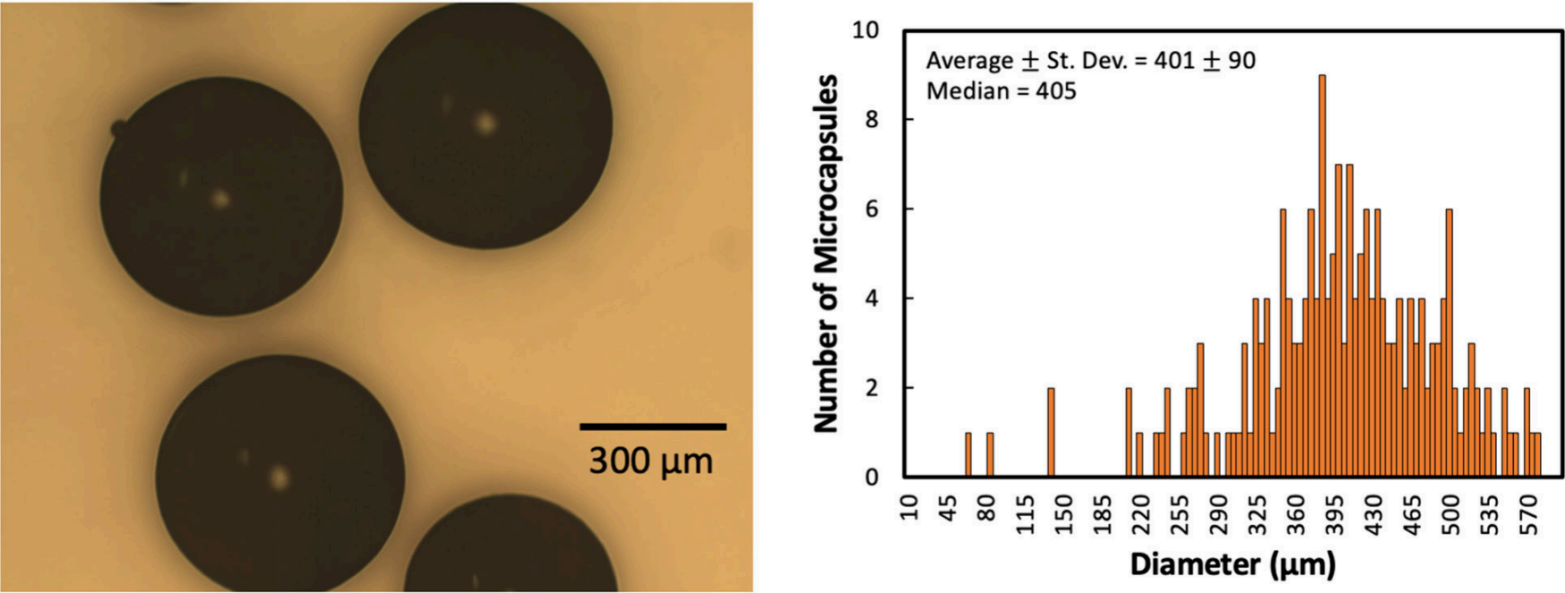
Characterization of high MW cPPA/DD microcapsules with 5 wt % HNT PAG. Larger spherical microcapsules were obtained compared to low MW cPPA/DD microcapsules. The size variances of microcapsules are shown with their average standard deviation (St. Dev.) and median.

Microcapsules with a high loading and uniform, spherical morphology were obtained when high MW cPPA was used, as shown in Figure 3. High MW cPPA was better able to encapsulate the oil core than low MW cPPA. Previous microcapsule studies discussing various morphologies, such as acorn-shaped structures, used low MW PPA (5 to 8 kDa in Eriksson et al.^50^ and 55 kDa in Tang et al.^34^). The weight of the DD core was about 2.4 times that of the cPPA shell, as calculated from ^1^H NMR analysis (Figure S2 and Table S2). This corresponds to a DD-to-cPPA volume ratio of about 3.84 using the DD density (0.75 g/mL^51^) and cPPA density (1.2 g/mL). Accordingly, the core diameter was calculated to be 24.8 times greater than the shell thickness (2.4 to 21.6 μm). The detailed calculation is explained in the Supporting Information. These thin-shell microcapsules are preferred because the role of the shell is to protect only the core, and the core is the payload to be delivered. A thin shell implies a higher payload content per unit microcapsule.

Low MW cPPA/DD microcapsules with 5 wt % BCSD were exposed to 248 nm radiation to study their depolymerization and core release profiles. ^1^H NMR was used to confirm that BCSD was not lost during emulsification (Figure S3 and Table S3). BCSD did not dissolve in DD, so most BCSD was likely contained in the cPPA shell. ^1^H NMR analysis before and after UV exposure showed no difference, and core release was not observed after UV exposure. This means that cPPA did not depolymerize after UV exposure, and the core remained trapped inside the cPPA shell.

Thermal treatment after UV exposure was used to increase the acid activity or mobility. When held at room temperature, the microcapsules visually appeared the same before and after UV exposure. In contrast, a post-exposure thermal treatment at 60 °C for 40 min caused the cPPA shell to depolymerize, resulting in an oily liquid mixture of DD core and *o*PA. TGA showed that unexposed cPPA/DD microcapsules with 5 wt % BCSD in the shell lost 3% of their weight during a 25 h heat treatment at 60 °C (Figure S4). Thus, the 40 min post-exposure heat treatment was not the sole cause of the BCSD activation. A 40 min heat treatment was chosen in this study to minimize the evaporation loss of the core and monomer produced.

The depolymerized cPPA and released core were collected using diethyl ether after UV irradiation and post-exposure heat treatment. The diethyl ether was evaporated by flowing dry nitrogen, and the residuals (a mixture of DD released and depolymerized cPPA) were analyzed by ^1^H NMR. The washed microcapsules were also analyzed by ^1^H NMR to quantify the extent of cPPA depolymerization and core release (Figures S5 to S10 and Table S4). A known amount of dimethylformamide (DMF) was added to the ^1^H NMR samples to quantify the concentration of the products. The integration peaks associated with cPPA (2H from 6.25 to 7.1 ppm), *o*PA (2H at 10.55 ppm), and DD oil (6H at 0.85 to 0.90 ppm) were compared to the DMF standard (6H from 2.88 to 2.95 ppm) to determine the respective mass loss or mass remaining in the microcapsules. A ^1^H NMR analysis is described in detail in the Supporting Information.

**Figure 4:**
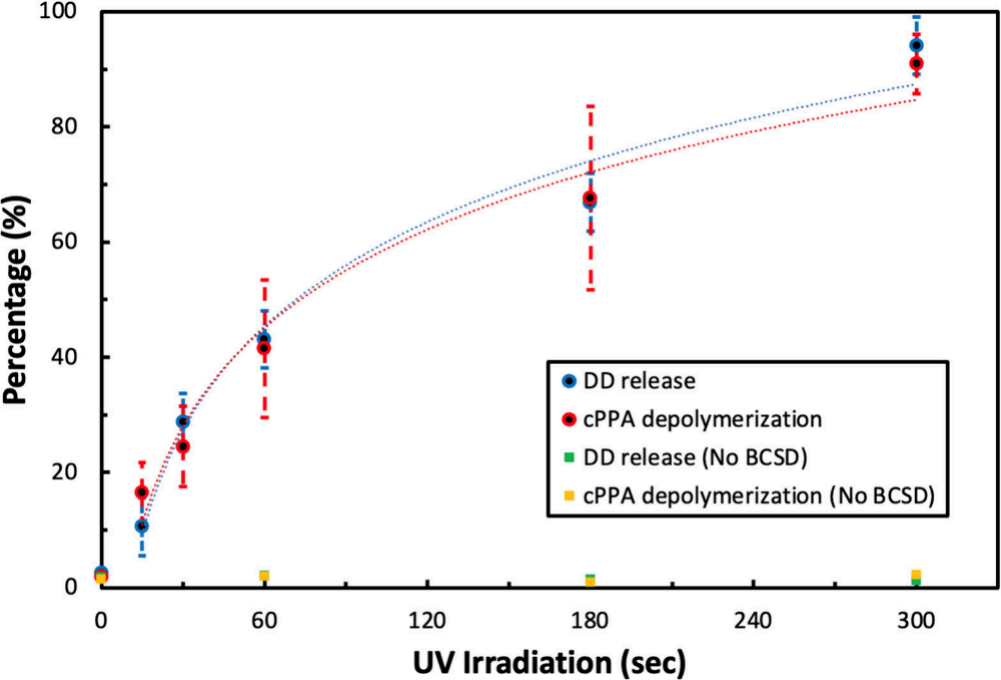
Core release and shell depolymerization profiles are shown for low MW cPPA/DD/BCSD microcapsules. The blue and red circles represent DD release and cPPA depolymerization, respectively, while green and yellow squares represent DD release and cPPA depolymerization from cPPA/DD microcapsules without BCSD. The error bars represent one standard deviation.

The core release and cPPA depolymerization profiles are shown in Figure 4, and the numerical values are tabulated in Table S4. Smaller microcapsules were easily broken, while larger microcapsules required a higher UV dose due to the greater path length for the 248 nm radiation. The size variance of the microcapsules was less than 100 μm (Figure 2).

Microcapsules without BCSD were UV irradiated as a control, and the data are shown in Figure 4. Less than 5% of cPPA depolymerization and DD release were obtained from microcapsules without BCSD even after 10.8 J/cm^2^ UV dose and heat treatment. The small residuals were likely caused by broken capsules or acorn-shaped structures. In the acorn-shaped structures, the core was exposed to the water phase during the emulsification process, and some DD remained on the microcapsules after filtration. This shows that cPPA did not depolymerize under UV irradiation, and PAG was necessary to photoactivate the cPPA shell.

The cPPA shells of the smaller microcapsules depolymerized quickly at low UV doses, as shown in Figure 4. The core release profile also followed this trend within experimental error because the core was extracted from the depolymerized shell. When the microcapsules were not exposed to UV radiation, approximately 2% of the DD was observed outside the microcapsules. The initial DD present on the microcapsules could interfere with microcapsule irradiation. cPPA/DD/5 wt % BCSD microcapsules remained mostly intact after high UV dose. Although N_2_ was released during the reaction, no gas generation effects, such as bubbling or expansion, were observed. After 24 h, 8.0 ± 6.3% of the core was released after a 10.8 J/cm^2^ UV dose and 11.1 ± 6.7% of the cPPA depolymerized. The microcapsules can be first exposed to 248 nm radiation followed by core release initiated by heat at the desired time.

Compared to the stable microcapsules in this study, Eriksson et al. achieved core release and shell depolymerization with a single UV exposure without incorporating PAG in the microcapsule shell.^50^ Also, their microcapsules ruptured, and the core was released after 1 h heat treatment at 90 °C.^50^ In contrast, the microcapsules made here without PAG maintained their structure during a 24 h, 90 °C heat treatment. Broken capsules were observed after 41 h at 90 °C thermal treatment (Figure S11). The difference in stability between these and Eriksson’s microcapsules^50^ is likely due to the type of PPA (cPPA vs lPPA) used in the microcapsule shell. lPPA is less stable due to the relative weakness of its end-caps compared to the acetal linkage; thus, it depolymerizes without external stimulus.^33^

**Figure 5:**
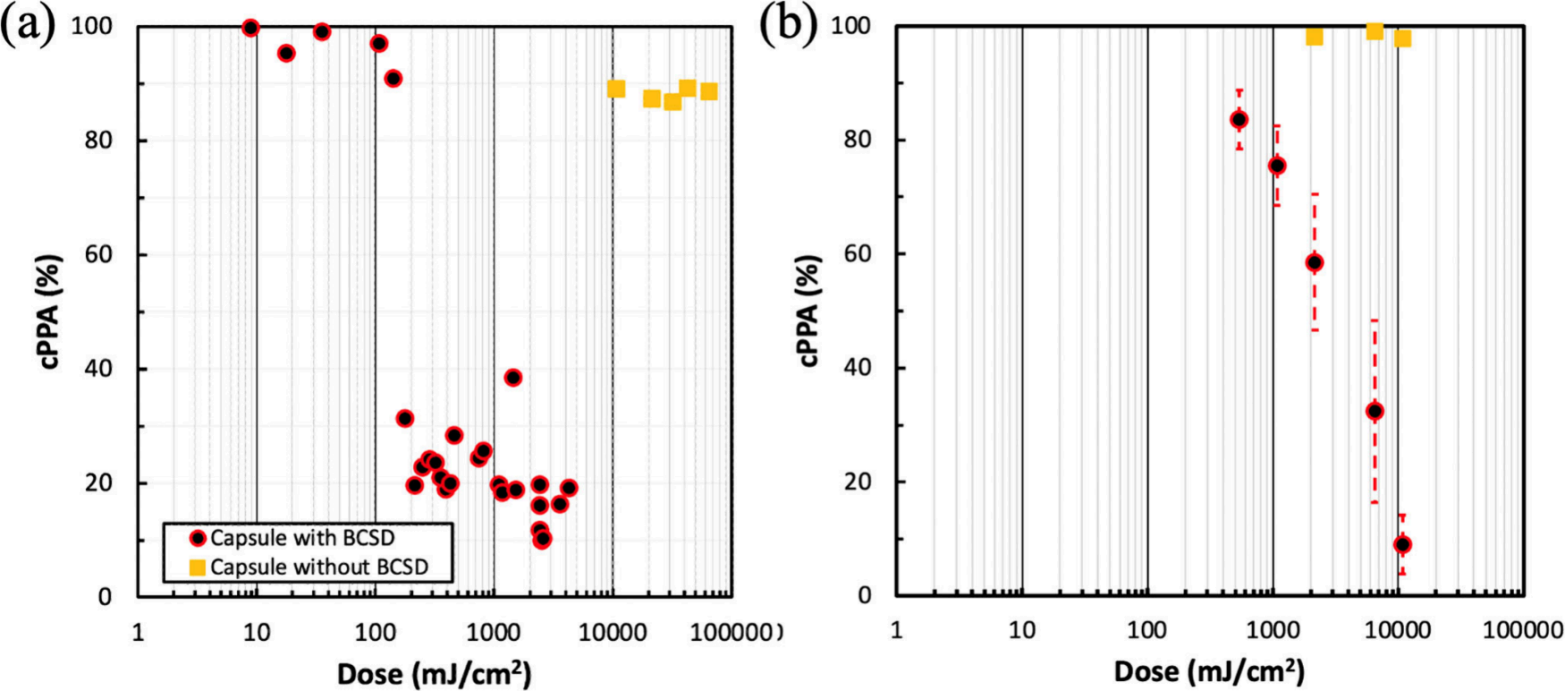
cPPA wt % vs UV dose (248 nm). (a) The remaining cPPA wt % in cPPA/DD microcapsules with 20 wt % BCSD after UV exposure and 240 min of heating at 60 °C is shown. (b) The results are converted from cPPA depolymerization percentage in Figure 4. The remaining cPPA wt % in UV-exposed cPPA/DD microcapsules with 5 wt % BCSD after 40 min of heating at 60 °C is shown.

The microcapsule weight change was investigated to quantify the cPPA depolymerization resulting from UV and heat treatments. cPPA/DD microcapsules with 20 wt % BCSD were used, and the cPPA weight change is plotted in Figure 5a. cPPA particles (i.e., microcapsules without core) were made by the same process described in the Experimental Methods section, except DD was excluded. The weight for the 20 wt % BCSD/cPPA particles (Figure S12) decreased for 250 min at 60 °C after UV exposure. The weight started to decrease after 290 min at 60 °C for samples without UV exposure. This means that 290 min of heating at 60 °C was required to thermally activate the PAG. In addition, a weight change was observed with 20 wt % BCSD/cPPA/DD microcapsules during the 250 min heat treatment. The weight change was assumed to be due to DD evaporation through the cPPA shell, which was about 50 wt % core loss. As a result, a 250 min heat treatment at 60 °C was chosen to examine the full effect of BCSD with UV exposure, and 50 wt % core loss was assumed for all measurements. The microcapsule weight change was converted into the cPPA weight change considering the initial amount of cPPA, DD, and BCSD to make the microcapsules. The cPPA wt % was compared before and after UV radiation and post-exposure heat treatment (Figure 5a).

The cPPA depolymerization (%) for the cPPA/DD microcapsules with 5 wt % BCSD was analyzed by ^1^H NMR (Figure 4) and converted to cPPA wt %, as shown in Figure 5b. A 0.144 J/cm^2^ UV dose caused a sudden drop in cPPA weight from 96 to 19 wt % when the cPPA had 20 wt % BCSD. A >1.0 J/cm^2^ UV dose was required for rapid cPPA weight change when 5 wt % BCSD was included in the shell. This shows that 20 wt % BCSD microcapsules required only 10% of the UV dose to achieve the same weight change compared to when 5 wt % BCSD was incorporation in the shell. A higher UV dose (0.2 J/cm^2^ to 10 J/cm^2^) was required when there was less BCSD (20 wt % to 5 wt %) in the cPPA shell, and a shorter post-exposure heating time (240 to 40 min) was required to achieve >80% cPPA weight change. As a control test, cPPA/DD microcapsules without BCSD were tested in the same manner resulting in 10 wt % loss after a 60 J/cm^2^ UV dose. The numerical values used in Figure 5 are listed in Table S5.

In addition to BCSD, Rhodorsil FABA PAG was used in the microcapsule shell because Rhodorsil FABA has high efficiency in PPA depolymerization.^38−43^ Rhodorsil FABA dissociates into an iodonium cation and an FABA anion during the emulsification process. Only the FABA anion was found to be present inside the microcapsules, as determined by ^1^H and ^19^F NMR (Figure S13). The solubility of the FABA ion in DCM solvent in the oil phase caused the FABA anions to be included in the microcapsules, while the iodonium cation was washed away during the emulsification process. Thus, Rhodorsil FABA was not pursued because the iodonium ions, the acid-generating portion of Rhodorsil FABA (Figure 1), were not present in the microcapsules. Nonionic PAGs are simpler since they do not dissociate during emulsification. As a result, nonionic PAGs are more appropriate for the emulsification process presented in this study.

**Figure 6:**
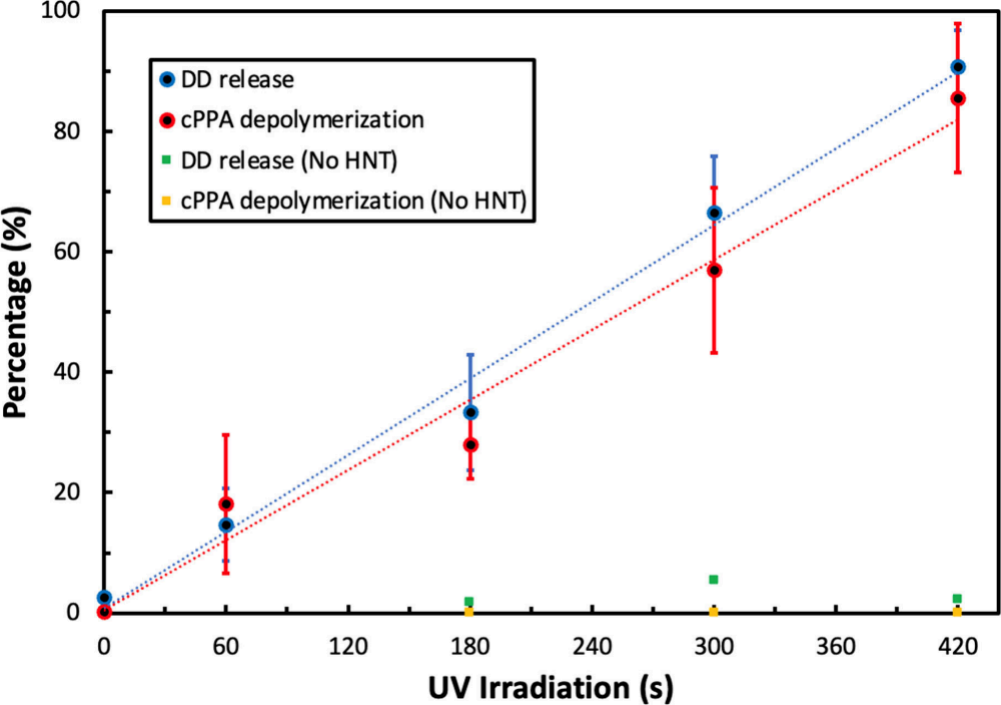
Core release and shell depolymerization profiles of high MW cPPA/DD/HNT microcapsules are shown. Blue and red circles represent DD release and cPPA depolymerization, respectively, while green and yellow squares represent DD release and cPPA depolymerization from cPPA/DD microcapsules without HNT, respectively. The error bars represent one standard deviation.

High MW cPPA/DD microcapsules with 5 wt % HNT were exposed to 365 nm radiation to study their degradation and release profile. ^1^H NMR was used to show that HNT was not lost during emulsification (Figure S2 and Table S1). HNT did not dissolve in DD, so most HNT was assumed to be present in the cPPA shell. The core release and cPPA depolymerization profiles after UV exposure are listed in Figure 6. ^1^H NMR used to quantify the core release (%) and cPPA depolymerization (%) are shown in Figures S14 to S18, and the numerical values are tabulated in Table S6. A linear trend with UV dose was observed (Figure 6). The relatively large error bars in Figure 6 are likely due to the wide size variance, as shown in Figure 3. A similar number of microcapsules degraded as the UV dose was increased. In contrast, a logarithmic change in capsule degradation and core release was observed with low MW cPPA microcapsules (Figure 4). The size variance was less than 200 μm (Figure 2). Many microcapsules degraded as a result of the initial UV dose, and the rate of microcapsule degradation slowed at higher UV doses.

Additionally, a greater UV dose was needed to obtain 80% core release with the larger, high MW cPPA/DD/HNT microcapsules (15.12 J/cm^2^, equivalent to 420 s UV irradiation) compared to the smaller, low MW cPPA/DD/BCSD (10.80 J/cm^2^, equivalent to 300 s UV irradiation) due to the thicker shell in the high MW microcapsules.

**Figure 7:**
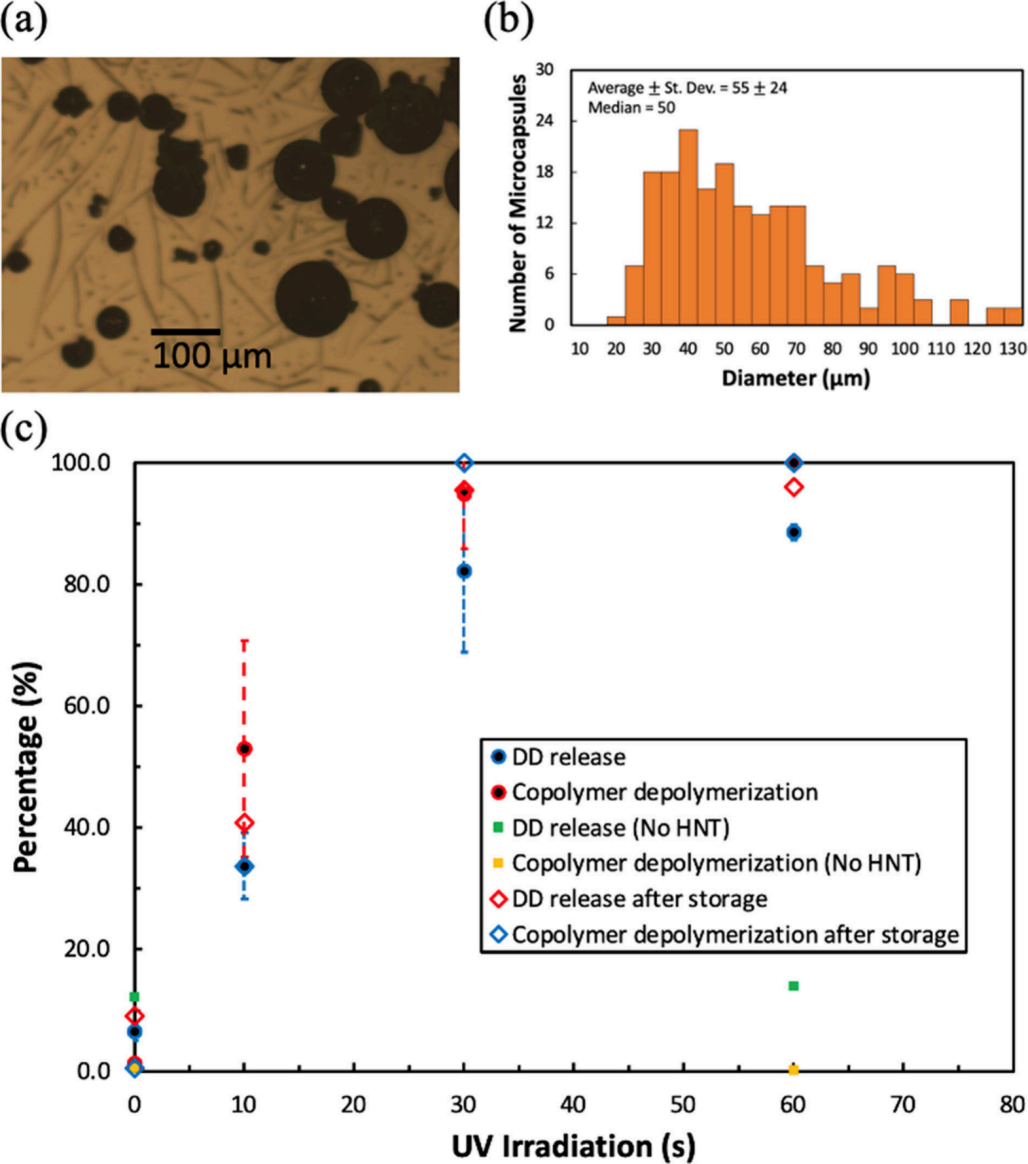
(a) Microscopic image, (b) size variance with the average standard deviation (St. Dev.) and median, and (c) core release and shell depolymerization profiles are shown for copolymer/DD/HNT microcapsules. The error bars represent one standard deviation. The microcapsules stored at 3 °C for two months were also investigated, and the data are shown as hollow diamond in (c).

Cyclic poly(phthalaldehyde-*co*-propanal) (cPcP) was used in place of cPPA in the microcapsule shell to improve photoactivation rates. It has previously been reported that the copolymer is easier to depolymerize than cPPA, and propanal has a higher vapor pressure than phthalaldehyde.^52^ cPcP with 75 mol % phthalaldehyde and 25 mol % propanal was used to make microcapsules with DD core and 5 wt % HNT PAG. 25 mol % propanal in the copolymer was the upper limit.^48^ This ratio was chosen for this study to maximize the impact of product’s vapor pressure. The microcapsule morphology and size variance are shown in Figures 7a and 7b, respectively. The core release and copolymer depolymerization profiles are shown in Figure 7c. ^1^H NMR was used to quantify the core release (%) and copolymer depolymerization (%), as shown in Figures S21 to S24. The numerical values are listed in Table S8. The MW of the copolymer (*M*_n_ = 19.5 kDa) was smaller than the low MW cPPA. The small size variance and less consistent shape are likely due to the low cPcP MW. As a control, cPcP and DD core microcapsules were made without HNT PAG. The core release and depolymerization were quantified using the same method that was employed for the homopolymer microcapsules (Figures 4 and 6).

A 10 wt % core release was obtained without UV exposure for microcapsules with and without HNT PAG. This initial core release was likely caused by broken capsules or acorn-shaped structures. The core was exposed to the water phase in acorn-shaped structures during the emulsification process, and some DD likely remained on the microcapsules after filtration. A broad peak of cPcP (0.82 to 1.18 ppm) overlapped with DD (0.85 to 0.90 ppm) at low doses, which interferes with DD quantification. The cPcP peaks were not observed after 1 min of UV exposure (365 nm, 2.16 J/cm^2^) because cPcP depolymerized, as shown in Figure S24. Only 1 min of UV exposure resulted in 100% shell depolymerization, as shown in Figure 7c. The copolymer microcapsules with 5 wt % HNT achieved over 80% core release after 30 s UV exposure (1.08 J/cm^2^). A similar result was obtained with cPPA/DD/HNT after 420 s of UV exposure (15.12 J/cm^2^). A 10 wt % core release was obtained after 60 s UV exposure (2.16 J/cm^2^) with the control copolymer microcapsules, which did not have HNT PAG. This shows that PAG is required to photoactivate the copolymer microcapsules as with the homopolymer microcapsules.

The homo/copolymer microcapsules made in this study remained intact at −85 and 3 °C for several months, showing that they can be stored for long periods of time. The core release and shell depolymerization were investigated after two months of storage at 3 °C. The data are shown in Figure 7c. No copolymer microcapsule degradation without UV exposure was observed. The extent of core release from the microcapsules was 10%. The core release of stored microcapsules after UV exposure was mostly within the experimental error of those exposed without storage. ^1^H NMR was used to quantify the core release and copolymer depolymerization (Figures S25 to S28), and the numerical values are tabulated in Table S9.

The thermal stability of the microcapsules fabricated in this study were investigated, as shown in Figure S29. The copolymer microcapsules had the lowest onset temperature at 116 °C and showed a faster weight change. The propanal monomer from the depolymerized copolymer evaporated quickly due to its low boiling point (48 °C).^53^ High MW cPPA microcapsules showed minimal weight loss up to 120 °C. The weight loss onset for high MW cPPA microcapsules was 160 °C. The high MW cPPA/5 wt % HNT microcapsules showed a weight loss starting at 120 °C. The DD core has a boiling point of 216.23 °C.^51^ Without PAG, the normalized weight approached zero at a high temperature. With PAG, a char residual was observed. It is possible that PAG underwent additional chemical reactions. Figure S29 shows that changing from cPPA to cPcP did not affect the thermal decomposition temperature.

## Conclusion

In this study, low MW and high MW cPPA were used to form microcapsules with DD oil cores by an emulsification process and the solvent evaporation method. Low MW cPPA gave various morphologies with random core loading, while high MW cPPA gave a uniform sphere morphology with larger microcapsule size and homogeneous core loading. Different PAGs were incorporated into the microcapsule shell to achieve photoactivated core release. BCSD required additional heat treatment after UV exposure. In contrast, HNT was activated by only UV exposure, so cPPA depolymerization and core release were obtained without post-exposure heat treatment. Microcapsules were also made using cyclic poly(phthalaldehyde–propanal) with 75 mol % phthalaldehyde and 25 mol % propanal. Shell depolymerization was achieved at a much lower UV dose due to the inherent sensitivity of the copolymer compared to the phthalaldehyde homopolymer.

This oil core delivery system can be used to transport other curing agents, paint adhesives, or hydrophobic catalysts for recycling or generation. For example, Grubbs-type catalysts were recently encapsulated to provide latency for frontal polymerization.^54^ cPPA microcapsules have been attempted to achieve UV-initiated frontal polymerization as our ongoing work. DCM was used as a solvent for polymerization and microcapsule fabrication in this study; however, it could be replaced by less hazardous solvents with similar polarity, solubility, and vapor pressure.

## Supporting Information Available

^1^H NMR spectra of homopolymer and copolymer, microcapsules, and the quantification of PAG in microcapsules; quantification of core diameter and shell thickness using high MW cPPA microcapsules; ^1^H NMR spectra of ether extract and washed microcapsules to quantify DD release (%) and cPPA depolymerization (%) and their integration values; isothermal thermal gravimetric analysis of cPPA/DD microcapsules and cPPA particles (i.e., microcapsules without core); optical images of cPPA/DD microcapsules under 90 °C heat treatment; dynamic thermal gravimetric analysis for the series of microcapsules used in this study (PDF)
