# Supplementary material for: Photoactivated Cyclic Polyphthalaldehyde Microcapsules for Payload Delivery
Source: ACS Appl Mater Interfaces. 2024 Aug 7;16(33):43951–60. doi: 10.1021/acsami.4c07609 (PMC11345721; doi:10.1021/acsami.4c07609)
Supplement: Supplementary file 1 — am4c07609_si_001.pdf [file am4c07609_si_001.pdf]

Supporting Information

Photo-Activated Cyclic Poly(phthalaldehyde) Microcapsules for Payload Delivery

Youngsu Shin[1], Jared M. Schwartz[1], Anthony C. Engler[2], Brad Jones[3], Oleg Davydovich[3], Paul A. Kohl[1]

[1]School of Chemical and Biomolecular Engineering, Georgia Institute of Technology, Atlanta, Georgia 30332

[2]Cain Department of Chemical Engineering, Louisiana State University, Baton Rouge, Louisiana 70803

[3]Sandia National Laboratories, Albuquerque, New Mexico 87185

*Email: kohl@gatech.edu

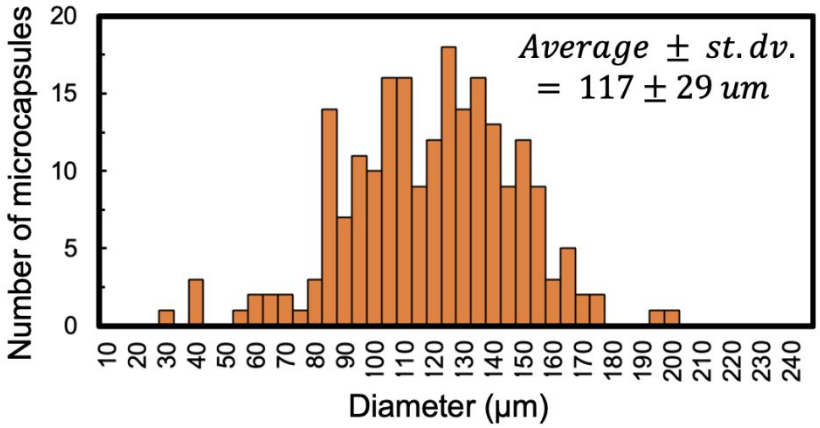

Figure S1. The size distribution of cPPA/DD microcapsules with 5% FABA (n=200) is shown.

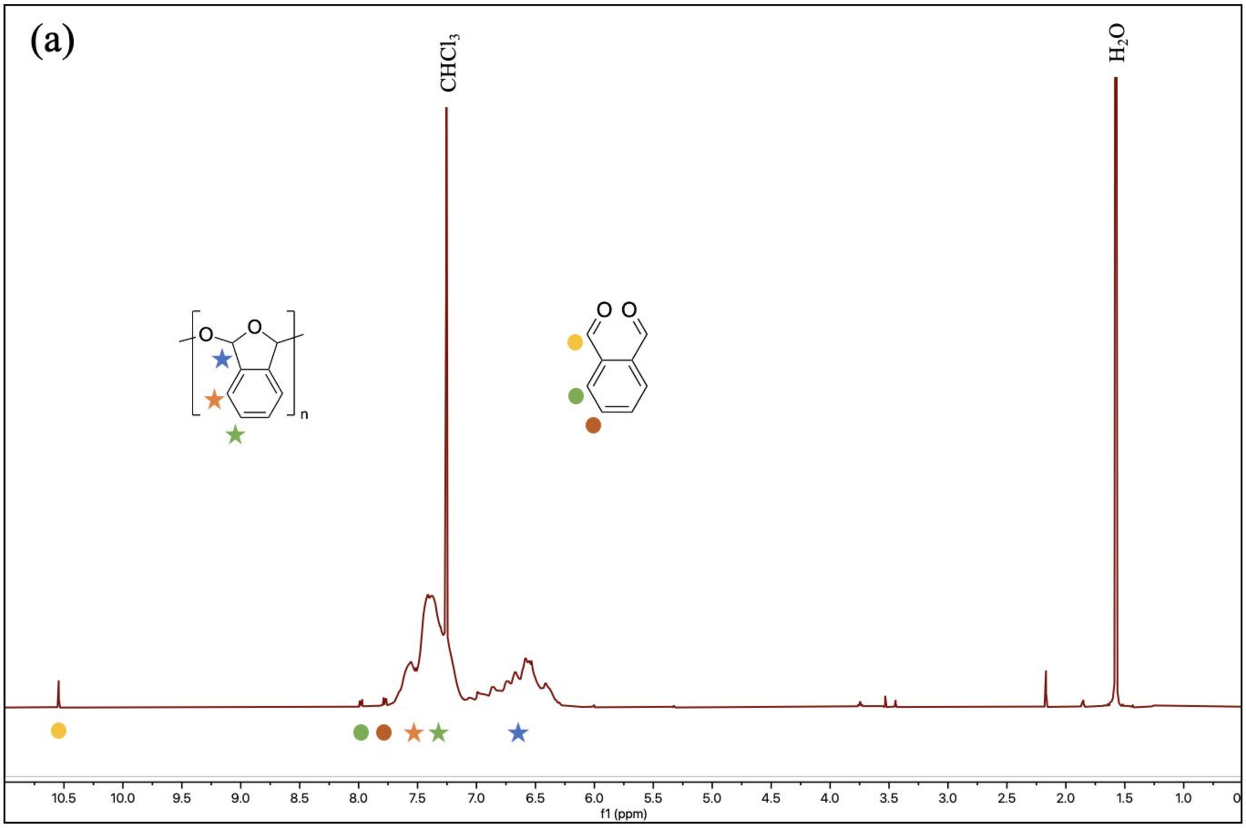

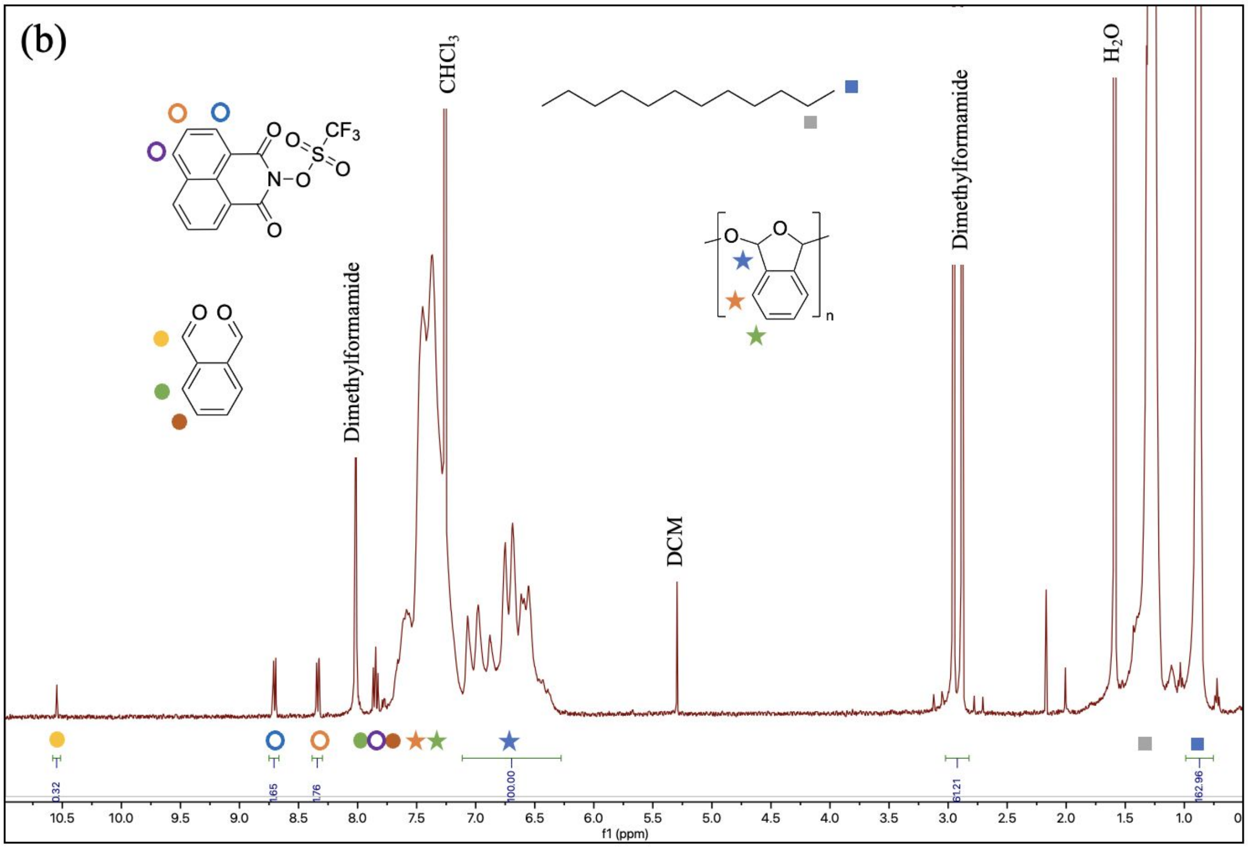

Figure S2. ^{1}H NMR spectra of (a) high MW cPPA used for microcapsule fabrication and (b) high MW cPPA/DD microcapsules with 5 wt% HNT are shown.

Table S1. Integration values (I.V.s) of cPPA and HNT in Figure S2 (b).

	cPPA	HNT
ppm	6.25 to 7.1	8.7
I.V.	100	1.92
The number of protons	2	2

HNT/cPPA wt% was calculated to be 4.94 %, close to the quantity of HNT added during microcapsule fabrication.

Five samples of high MW cPPA/DD microcapsules w/ 5wt% BCSD were used to calculate the shell thickness and core diameter by ^{1}H NMR spectra analysis. One example is shown in Figure S2. The integral values of five samples are tabulated in Table S2.

Table S2. cPPA (mg)/DD (mg) in high MW cPPA/DD/5 wt% BCSD microcapsule, using Integration values (I.V.s).

	cPPA	DD	
ppm	6.25 to 7.1	0.85 to 0.90	
The number of protons	2	2	
	I.V.		cPPA (mg)/DD (mg)
Sample 1 (Figure S2. (b))	32.6	20	2.50
Sample 2	31.7	20	2.43
Sample 3	33.3	20	2.55
Sample 4	31.5	20	2.41
Sample 5	31.4	20	2.41

cPPA (mg)/DD (mg) was calculated by using the molecular weight of each component and the I.V.s.

The average with one standard deviation of cPPA (mg)/DD (mg) is 2.46 $\pm$ 0.06. Assuming concentric geometry of the core and microcapsule, the volume ratio was calculated to be 3.84. According to Figure 3, the diameter of microcapsules was 65 to 580 μm. Diameter of the liquid core and the thickness of the shell were calculated to be 60 to 536 μm and 2.4 to 21.6 μm, respectively. As a result, the core diameter was 24.8 times greater than the cPPA shell thickness.

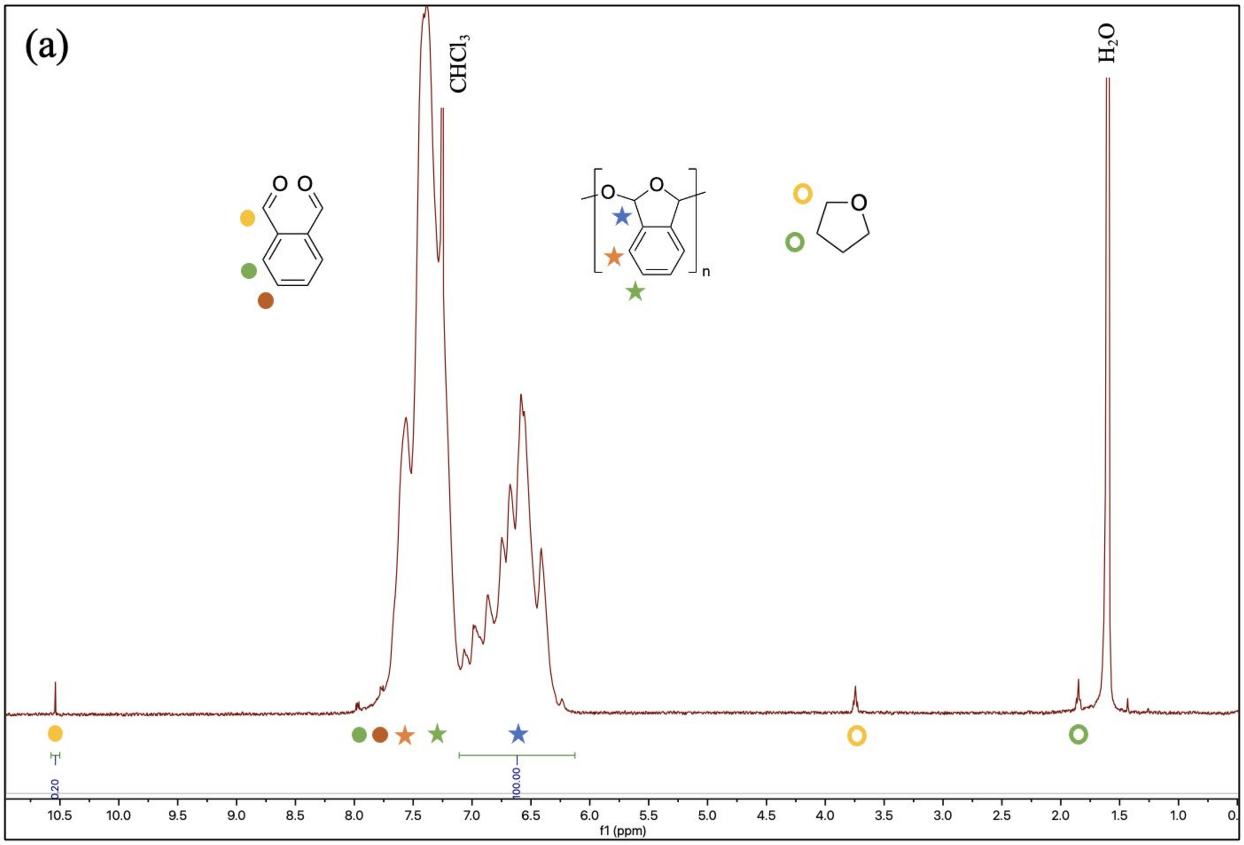

(a)
CHCl3
H2O
n
0.20
100.00
10.5
10.0
9.5
9.0
8.5
8.0
7.5
7.0
6.5
6.0
5.5
5.0
4.5
4.0
3.5
3.0
2.5
2.0
1.5
1.0
f1 (ppm)

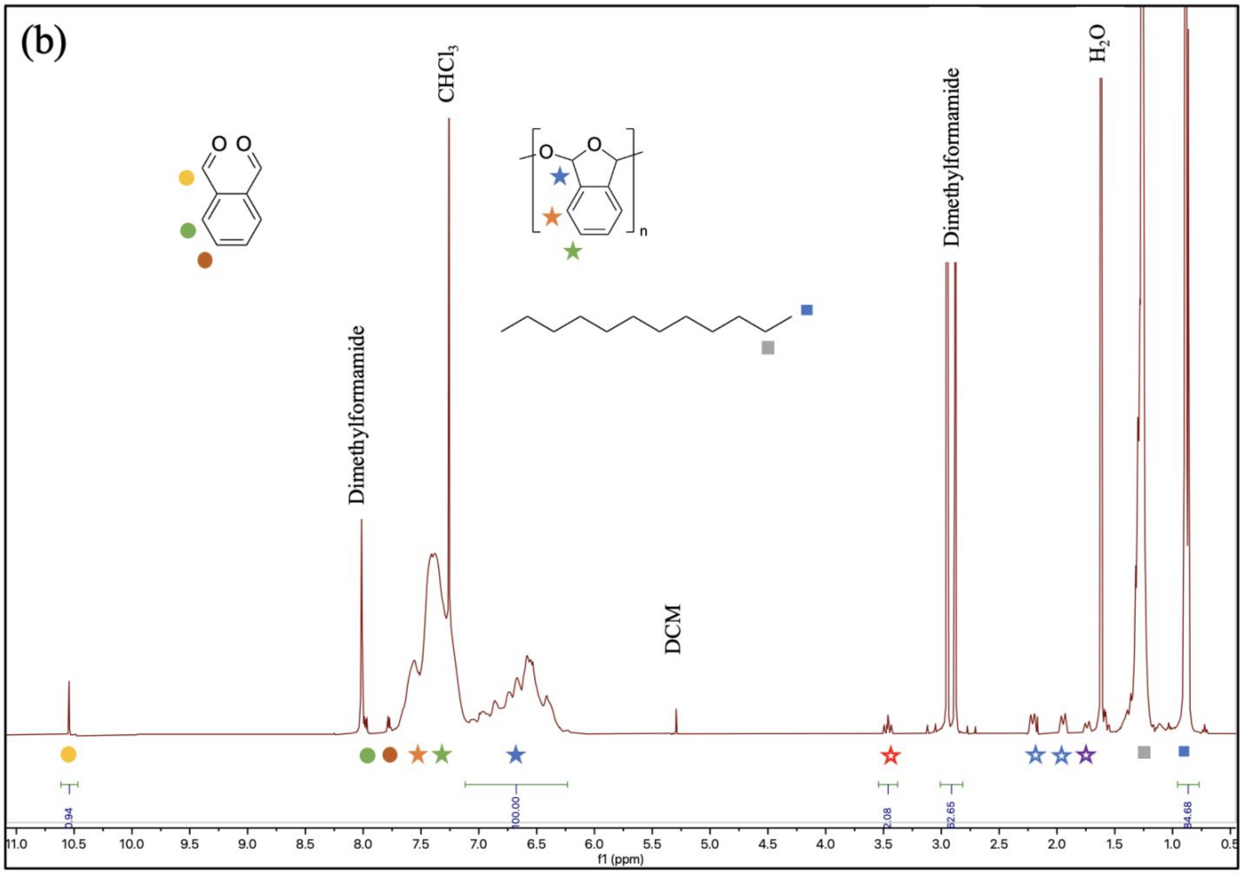

Figure S3. ^{1}H NMR spectra of (a) low MW cPPA used for microcapsule fabrication and (b) low MW cPPA/DD microcapsules with 5 wt% BCSD are shown. A small amount of THF impurities was left and shown in the spectrum.

Table S3. Integration values (I.V.s) of cPPA and BCSD.

	cPPA	BCSD
ppm	6.25 to 7.1	3.5
I.V.	100	2.02
The number of protons	2	1

BCSD/cPPA wt% was calculated to be 5.04 %, close to the quantity of BCSD added during microcapsule fabrication.

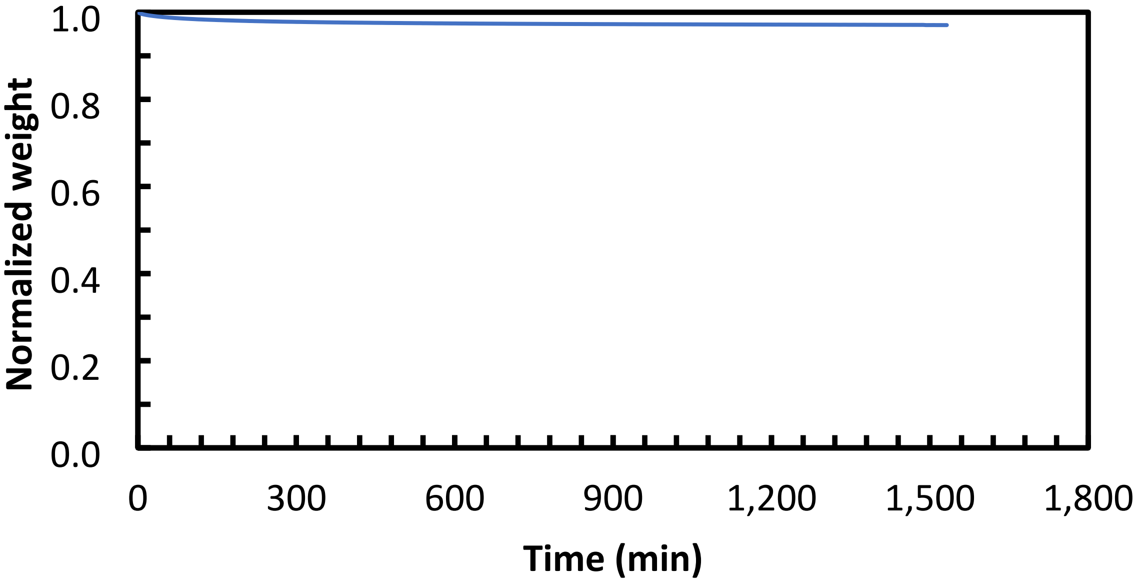

Figure S4. Isothermal thermal gravimetric analysis of cPPA/DD microcapsules with 5 wt% BCSD at 60 ℃ is shown. 3 wt% change was observed over 25 hr heat treatment.

Figure S5 to S10 show ^{1}H NMR spectra of low MW cPPA/DD microcapsules with 5 wt% BCSD after a diethyl ether wash. The NMR spectra were analyzed to quantify DD release (%) and cPPA depolymerization (%). The integration values used to construct Figure 4 are tabulated in Table S4. To quantify the contents of microcapsules, 8 – 12 mg of microcapsules were dissolved in $CDCl_3$ containing a precisely controlled amount of dimethylformamide (DMF). The peak integrations associated with PPA (2H from 6.25 – 7.1 ppm), monomer (2H at 10.55 ppm) and DD oil (6H at 0.85 to 0.90 ppm) were compared to DMF (6H from 2.88 – 2.95 ppm) to calculate the respective mass loss or mass remaining.

The mass of chemical A is quantified using the equation below.

$$m_A = \frac{IV_A \cdot MW_A \cdot \#H_{DMF}}{IV_{DMF} \cdot MW_{DMF} \cdot \#H_A} \cdot m_{DMF} (S1)$$

where IV_A is an integration value of the peak associated with a chemical specie A, $\#H_A$ is the number of hydrogens of the corresponding peak, MW_A is a molecular weight of a chemical specie A, and m_{DMF} is the mass of DMF added to the NMR sample.

The mass of chemical species from ^{1}H NMR was used to calculate DD release from diethyl ether extract (DE) and DD trapped in washed microcapsules (WM) and to calculate depolymerized PPA by UV and heat treatment as shown below.

$$DD\ release\ (\%) = \frac{m_{DD} in\ DE}{m_{DD} in\ DE + m_{DD}\ in\ WM} \cdot 100(\%) (S2)$$

$$cPPA\ depolymerization\ (\%) = \frac{m_{monomer}\ in\ DE + m_{monomer}\ in\ WM}{m_{PPA}\ in\ WM + m_{monomer}\ in\ DE + m_{monomer}\ in\ WM} \cdot 100(\%) (S3)$$

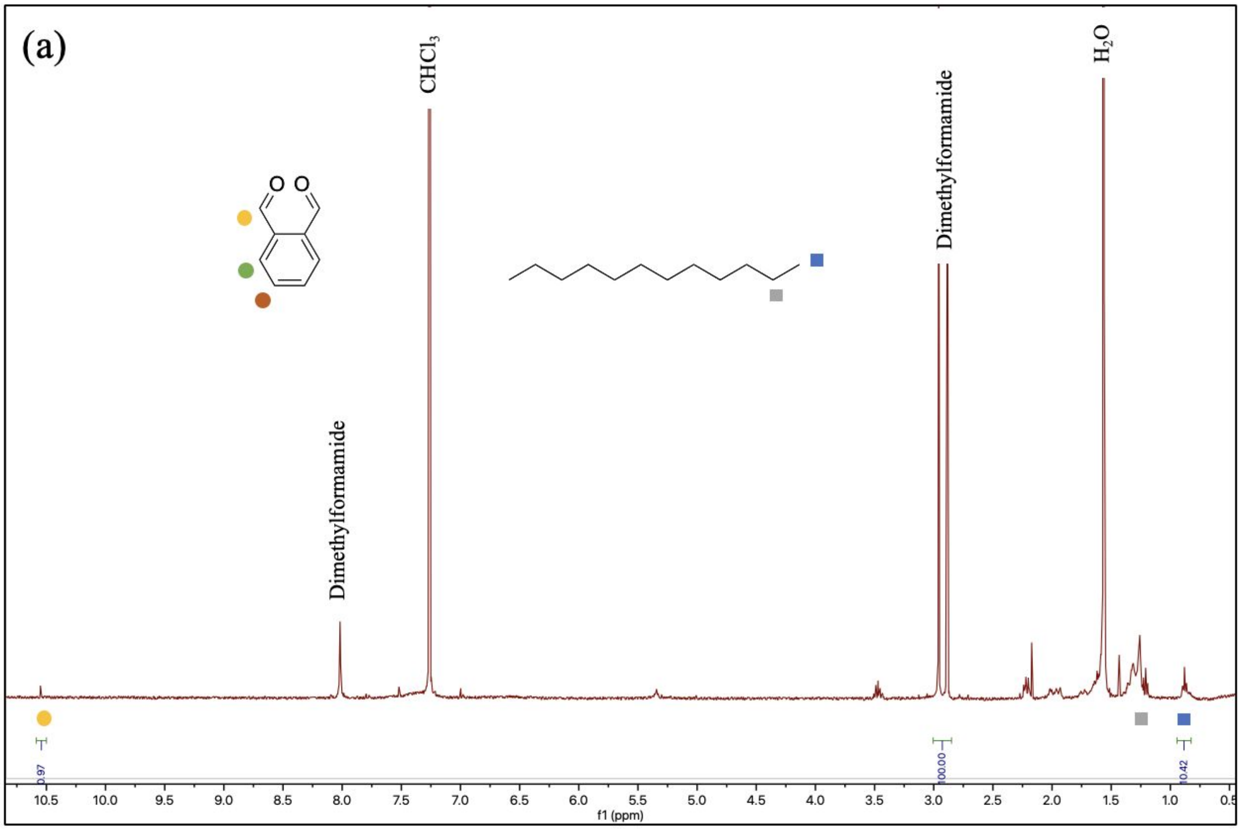

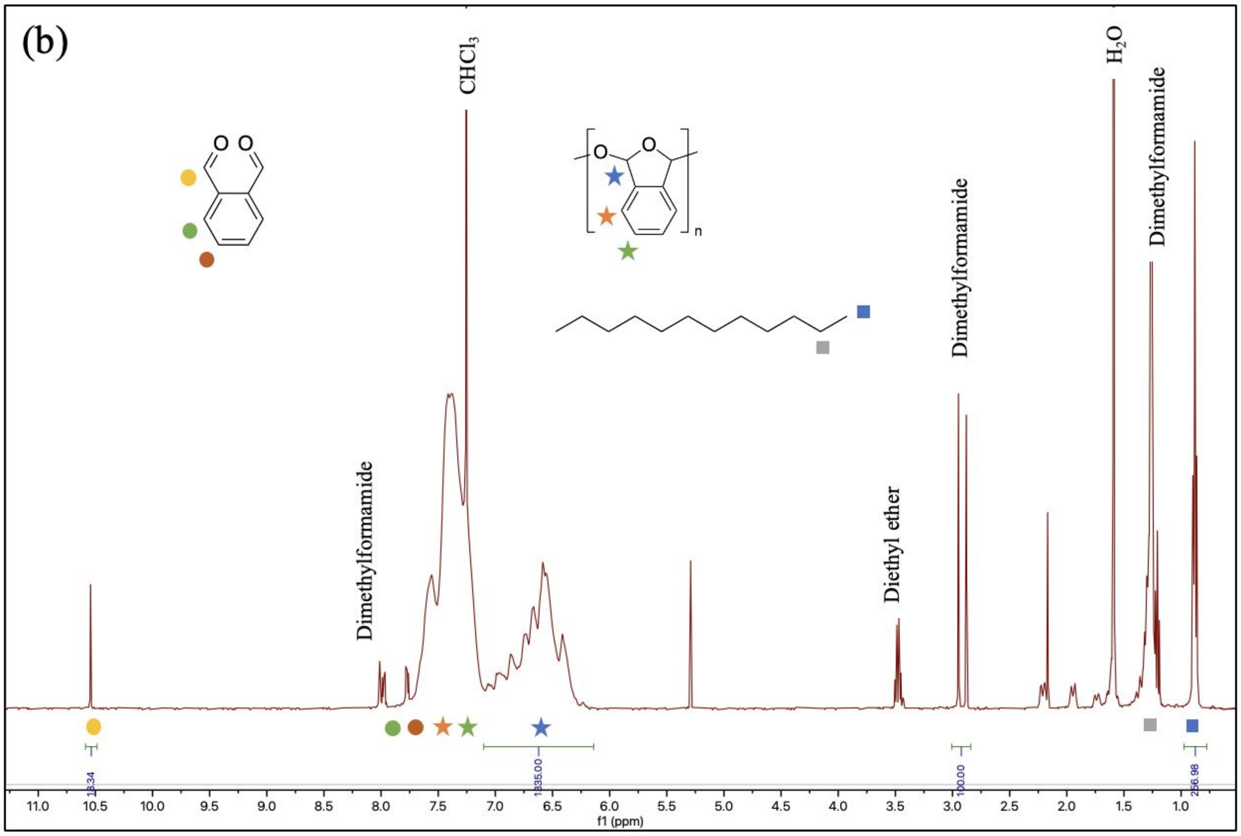

Figure S5. (a) ^{1}H NMR spectrum of washed low MW cPPA/DD/BCSD microcapsules shows the residuals from diethyl ether extraction after 0 s of UV irradiation. (b) ^{1}H NMR spectrum is shown for the remaining microcapsules which were washed with diethyl ether and dissolved in $CDCl_3$.

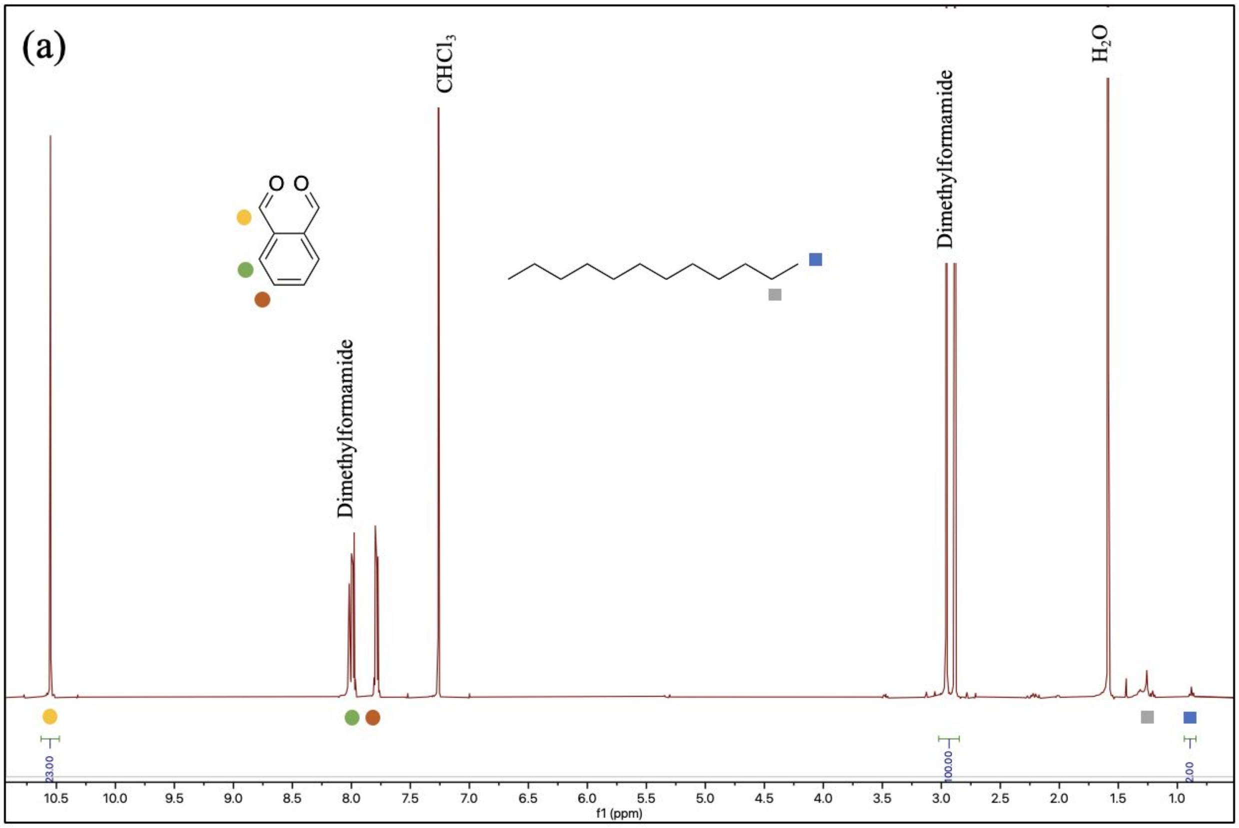

(a)
CHCl3
Dimethylformamide
H2O
Dimethylformamide
23.00
100.00
2.00
f1 (ppm)

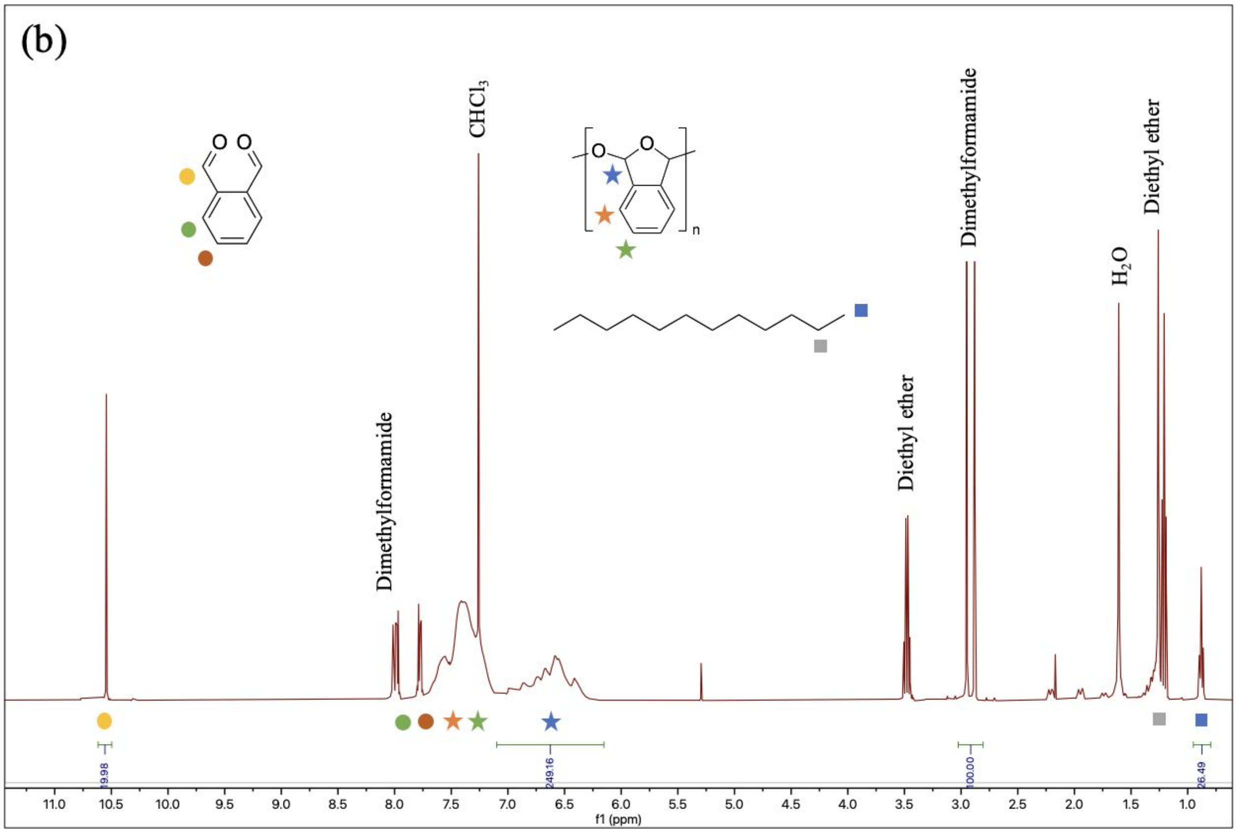

(b)
CHCl3
Dimethylformamide
Diethyl ether
H2O
Dimethylformamide
Diethyl ether
n
249.16
100.00
26.49
f1 (ppm)

Figure S6. (a) ^{1}H NMR spectrum of washed low MW cPPA/DD/BCSD microcapsules shows the residuals from diethyl ether extraction after 15 s of UV irradiation. (b) ^{1}H NMR spectrum is shown for the remaining microcapsules which were washed with diethyl ether and dissolved in $CDCl_3$.

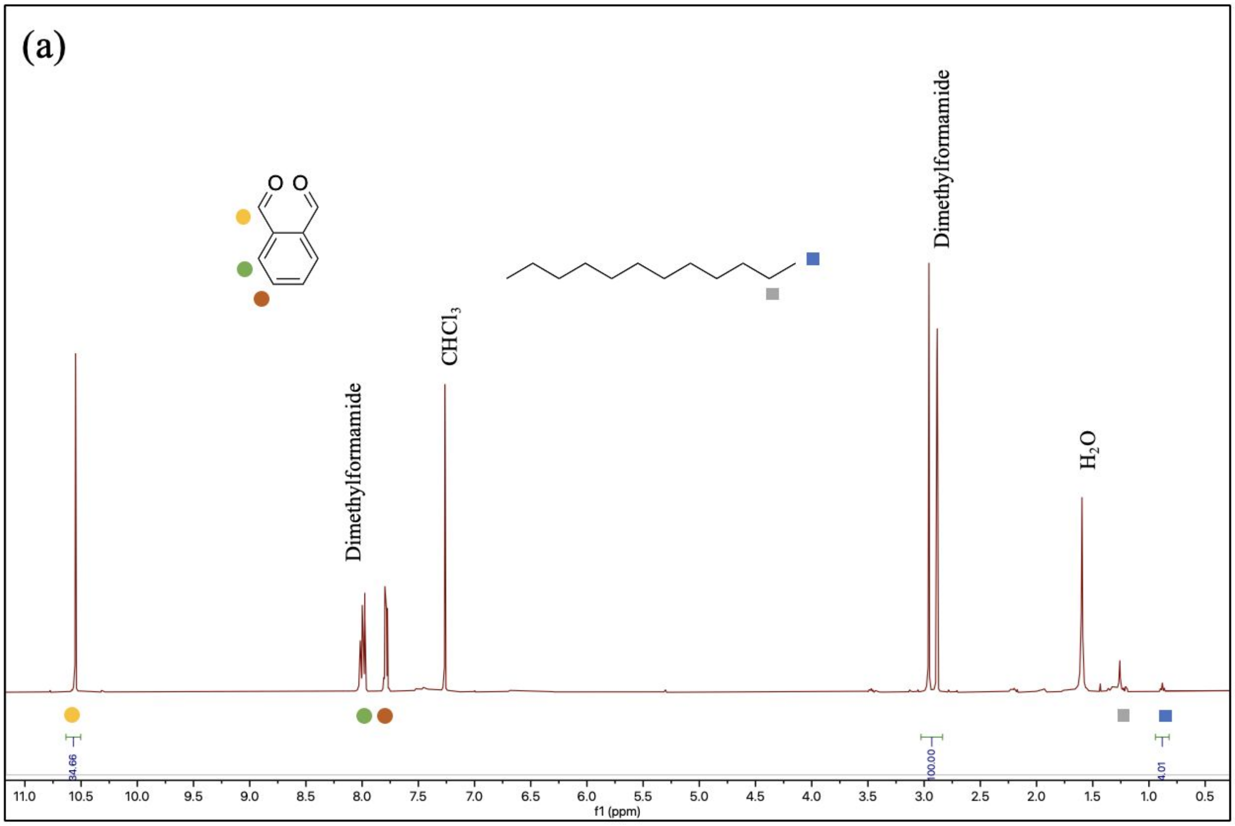

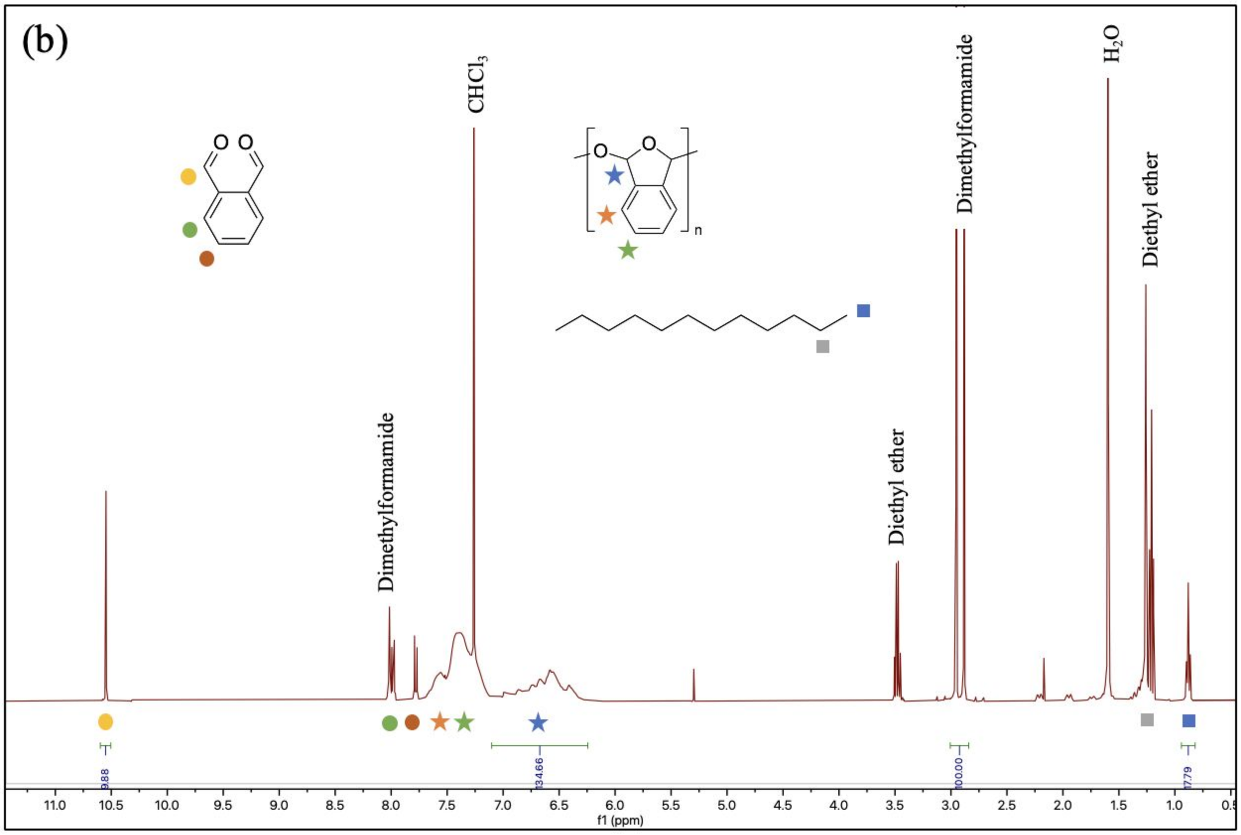

Figure S7. (a) ^1H NMR spectrum of washed low MW cPPA/DD/BCSD microcapsules shows the residuals from diethyl ether extraction after 30 s of UV irradiation. (b) ^1H NMR spectrum is shown for the remaining microcapsules which were washed with diethyl ether and dissolved in $CDCl_3$.

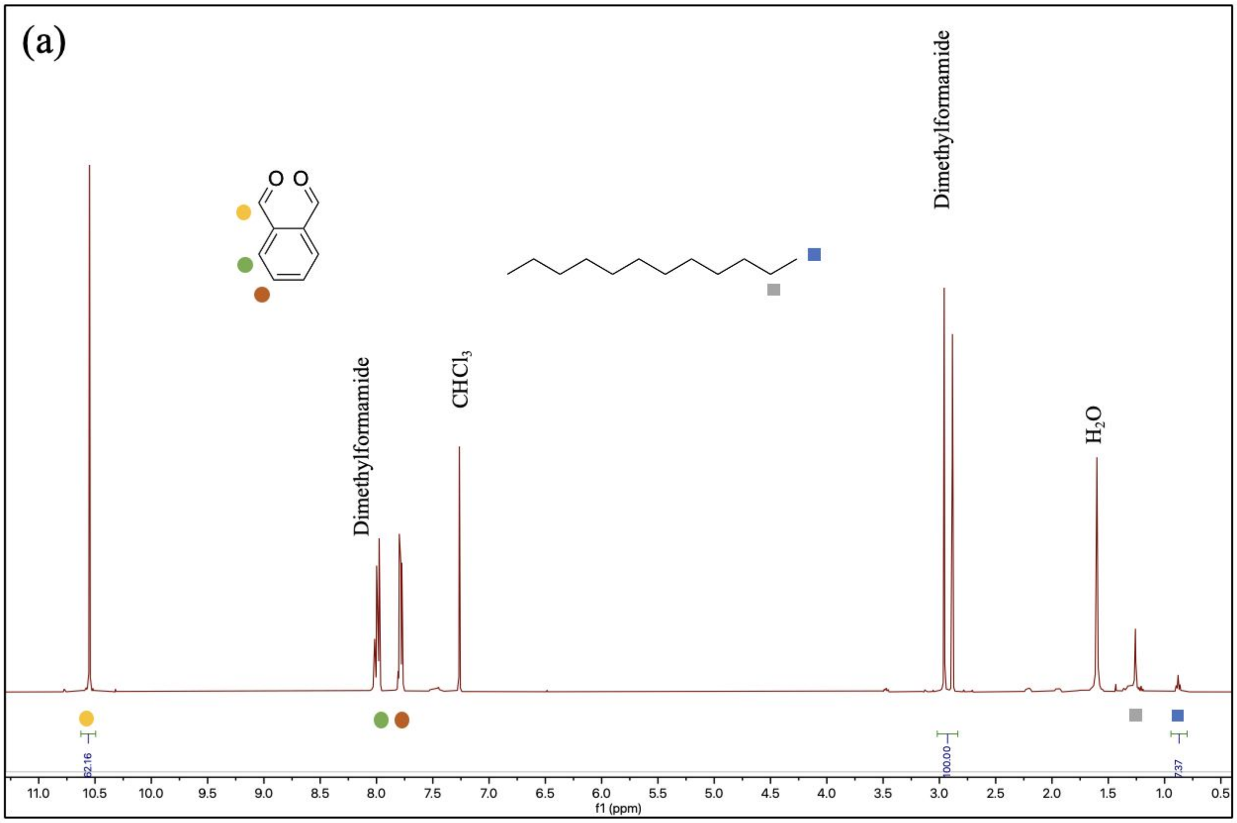
(a)
Dimethylformamide
Dimethylformamide
CHCl3
H2O
62.16
100.00
7.37
f1 (ppm)

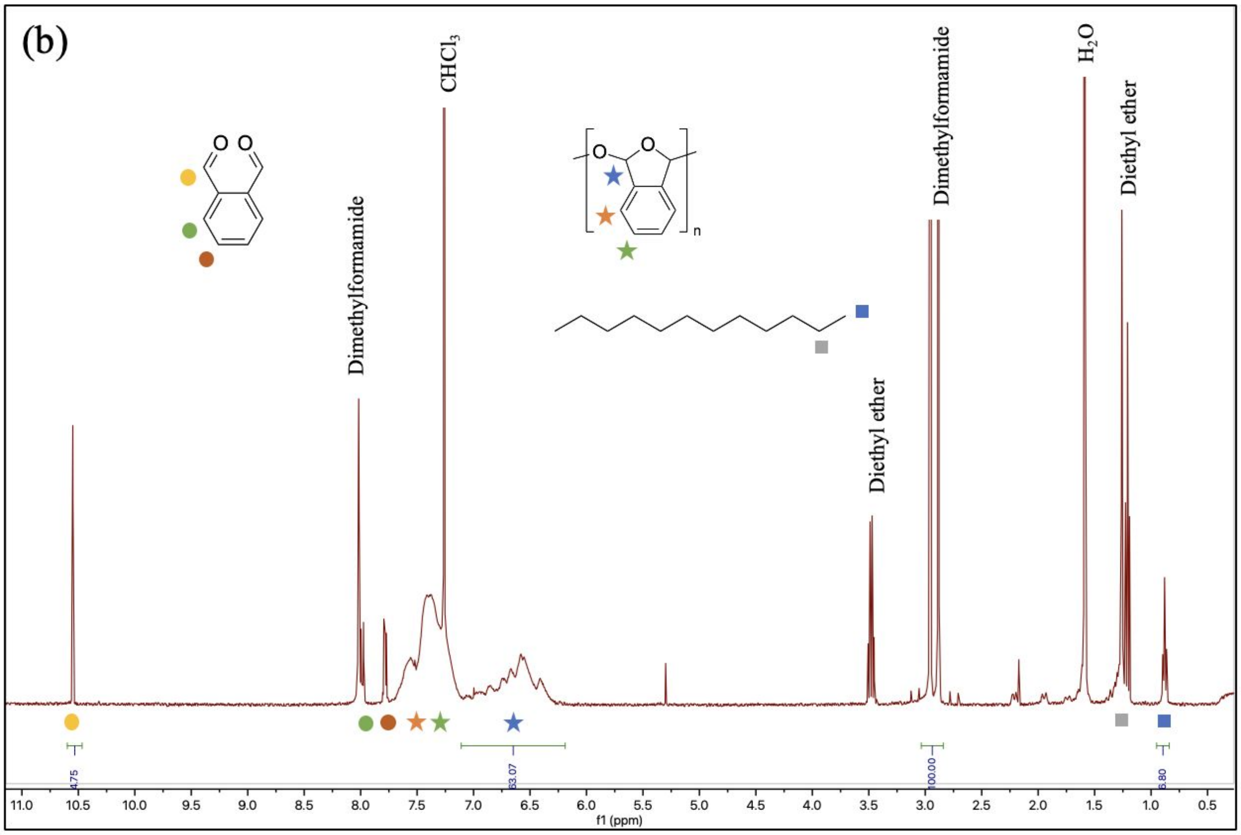
(b)
Dimethylformamide
CHCl3
Diethyl ether
Dimethylformamide
H2O
Diethyl ether
4.75
63.07
100.00
6.80
f1 (ppm)

Figure S8. (a) ^{1}H NMR spectrum of washed low MW cPPA/DD/BCSD microcapsules shows the residuals from diethyl ether extraction after 60 s of UV irradiation. (b) ^{1}H NMR spectrum is shown for the remaining microcapsules which were washed with diethyl ether and dissolved in $CDCl_3$.

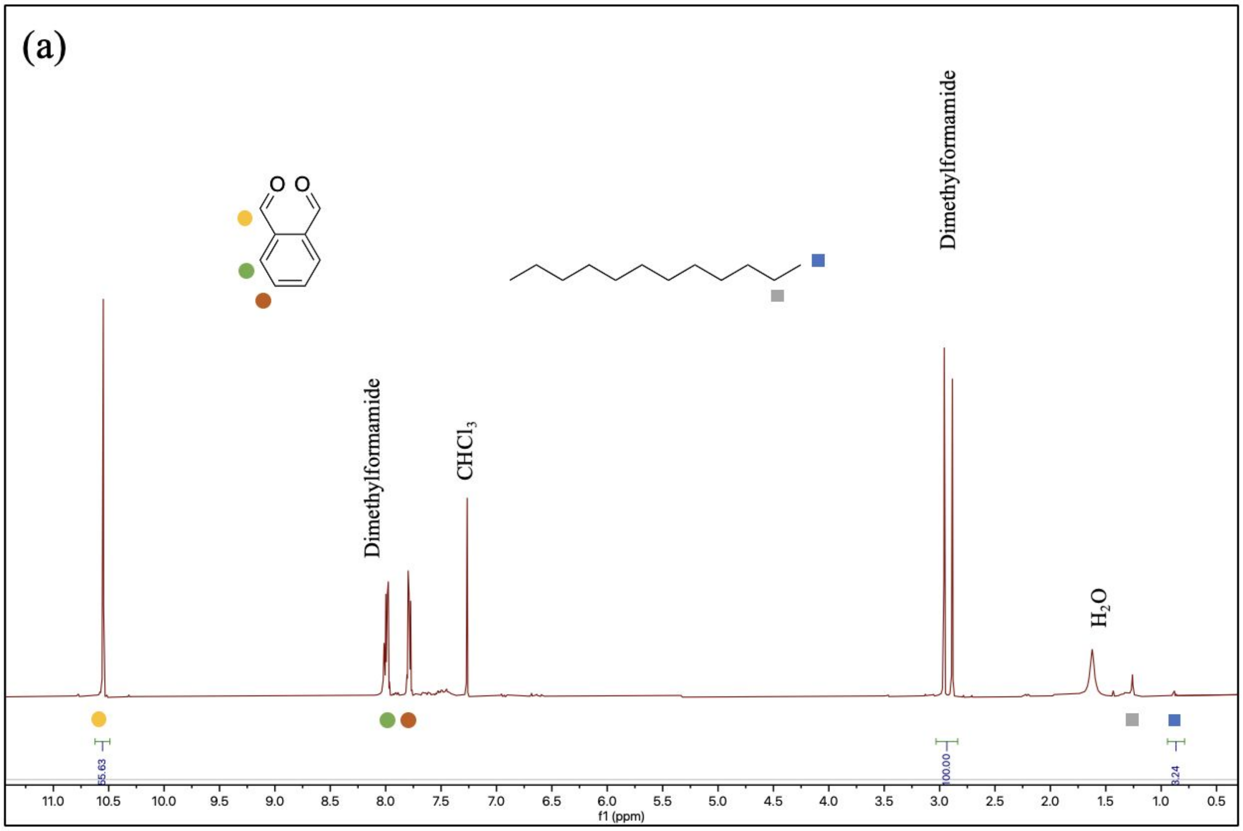

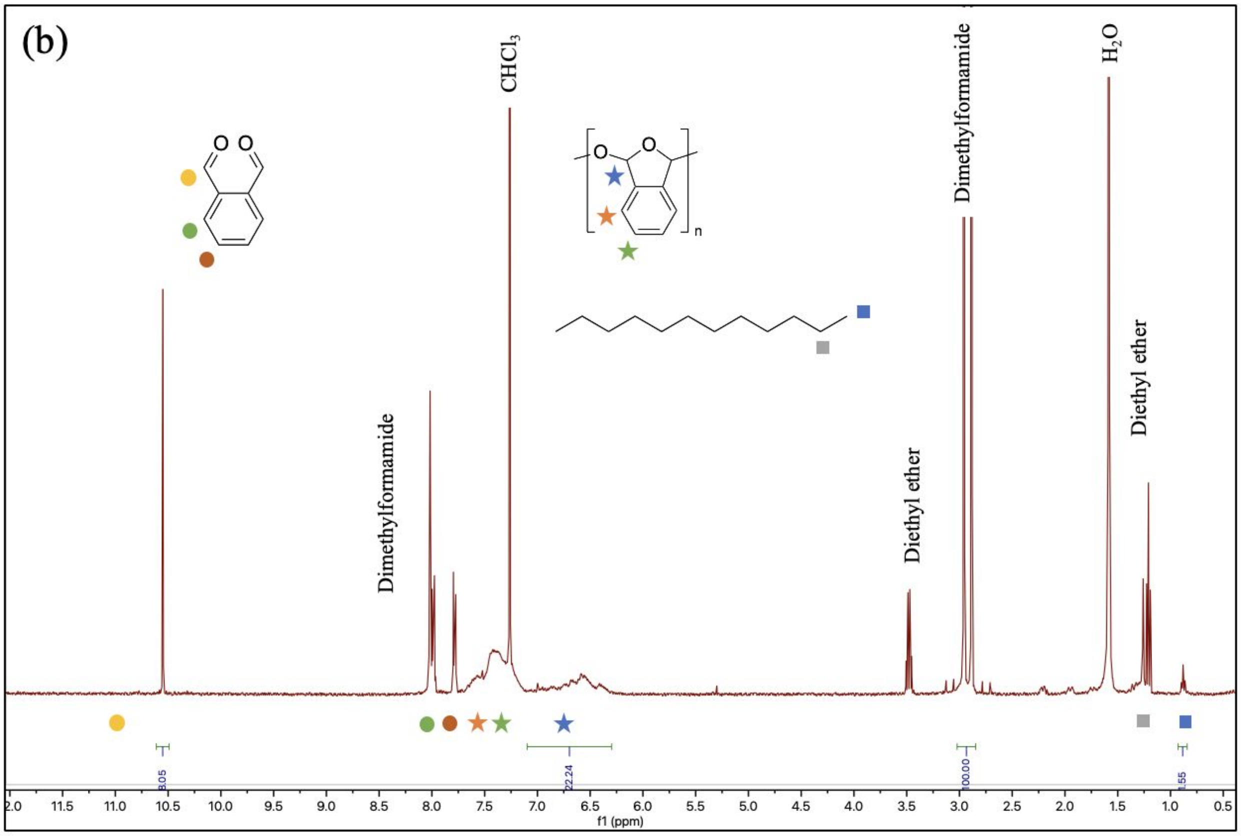

Figure S9. (a) ^{1}H NMR spectrum of washed low MW cPPA/DD/BCSD microcapsules shows the residuals from diethyl ether extraction after 180 s of UV irradiation. (b) ^{1}H NMR spectrum is shown for the remaining microcapsules which were washed with diethyl ether and dissolved in $CDCl_3$.

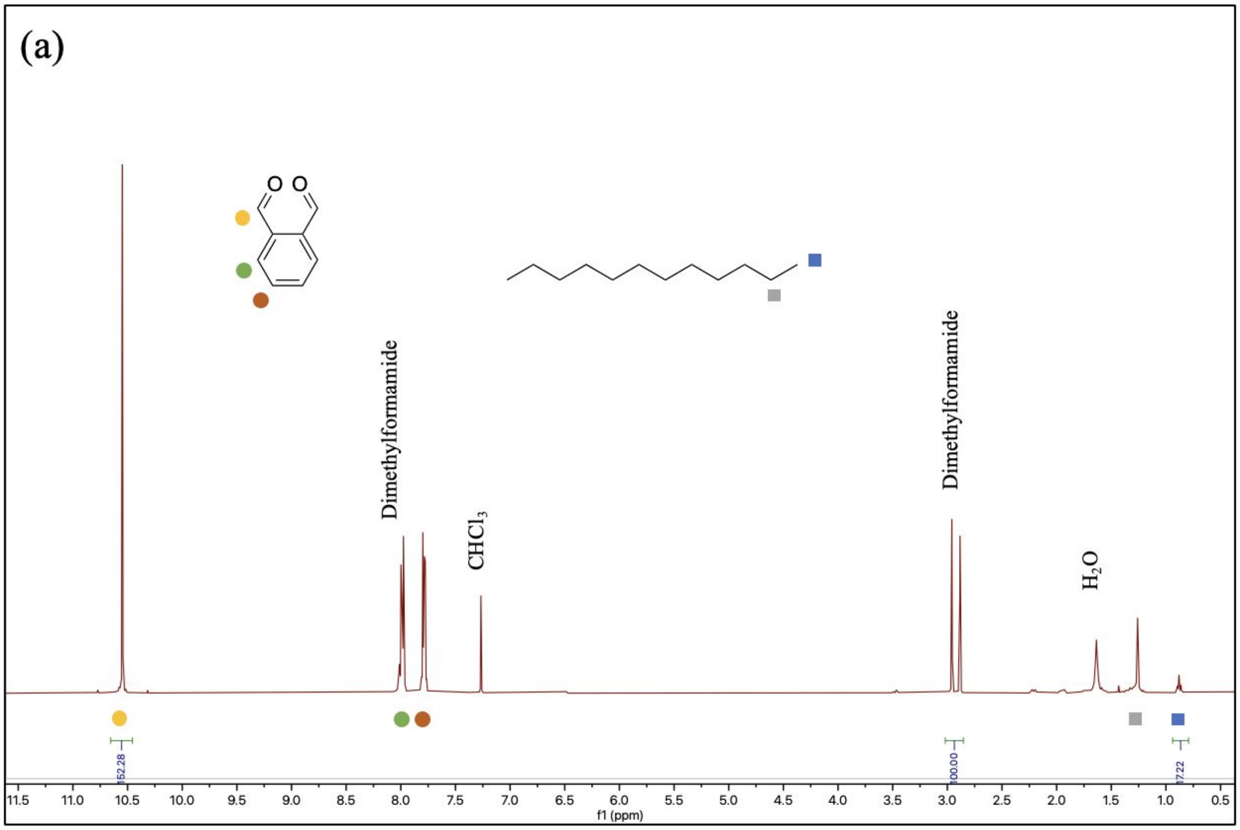
(a)
Dimethylformamide
CHCl3
Dimethylformamide
H2O
152.28
100.00
17.22
11.5
11.0
10.5
10.0
9.5
9.0
8.5
8.0
7.5
7.0
6.5
6.0
5.5
5.0
4.5
4.0
3.5
3.0
2.5
2.0
1.5
1.0
0.5
f1 (ppm)

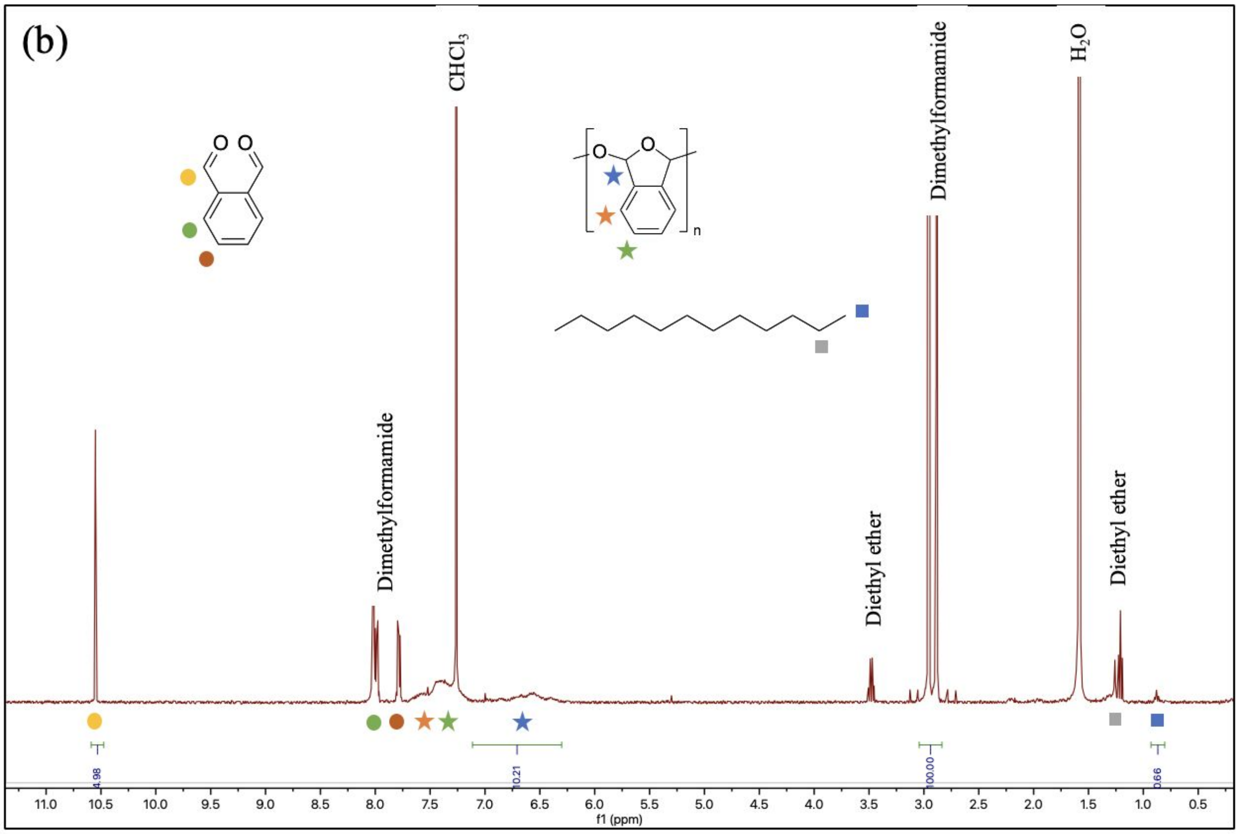

Figure S10. (a) ^{1}H NMR spectrum of washed low MW cPPA/DD/BCSD microcapsules shows the residuals from diethyl ether extraction after 300 s of UV irradiation. (b) ^{1}H NMR spectrum is shown for the remaining microcapsules which were washed with diethyl ether and dissolved in $CDCl_3$.

Table S4. (a) The core (DD) release and cPPA depolymerization values in Figure 4 are shown. (b) – (h) Integration values (I.V.s) to calculate DD release and cPPA depolymerization values after UV irradiation are tabulated. Sample 1, 2, and 3 are different batches of emulsification to form microcapsules. Sample 1-1, 1-2, and 1-3 are duplicates from Sample 1 batch.

(a) Numerical values of Figure 4

	5 wt% BCSD microcapsules		No PAG microcapsule (control)	
UV irradiation	DD release (%)	cPPA depolymerization (%)	DD release (%)	cPPA depolymerization (%)
0 s	2.6 ± 1.1	1.87 ± 0.61	1.8	1.5
15 s	10.6 ± 4.9	16.5 ± 5.2	-	-
30 s	29 ± 12	24.5 ± 7.0	-	-
60 s	43 ± 17	42 ± 12	2.0	2.0
180 s	67 ± 24	68 ± 16	1.5	1.0
300 s	94.1 ± 5.7	90.9 ± 5.2	1.2	2.2

(b) 0 s UV irradiation on 5 wt% BCSD microcapsules

		DMF	DD	cPPA	Monomer
Peak (ppm)		2.88 to 2.95	0.85 to 0.90	6.25 to 7.1	10.55
The number of protons		6	6	2	2
			I.V.		
Sample 1-1	In DE	100	3.7	0.0	0.7
	In WM	100	127.5	153.4	1.8
Sample 1-2	In DE	100	3.6	0.0	0.8
	In WM	100	263.8	314.4	5.6
Sample 1-3	In DE	100	4.0	0.0	1.3
	In WM	100	239.6	280.5	6.6
Sample 2-1	In DE	100	1.4	0.0	0.4
	In WM	100	62.6	226.4	3.9
Sample 3-1	In DE	100	10.4	0.0	1.0
(Figure S5)	In WM	100	257.0	1335.0	13.3

(c) 15 s UV irradiation on 5 wt% BCSD microcapsules

		DMF	DD	cPPA	Monomer
Peak (ppm)		2.88 to 2.95	0.85 to 0.90	6.25 to 7.1	10.55
The number of protons		6	6	2	2
			I.V.		
Sample 2-1	In DE	100	4.8	0.0	14.3
	In WM	100	24.7	135.0	27.6
Sample 2-2	In DE	100	10.5	0.0	86.7
	In WM	100	158.7	910.7	83.0
Sample 2-3	In DE	100	20.2	0.0	149.9
	In WM	100	163.9	812.9	30.2
Sample 3-1	In DE	100	2.0	0.0	23.0
(Figure S6)	In WM	100	26.5	249.2	20.0
Sample 3-2	In DE	100	5.0	0.0	48.9
	In WM	100	46.7	333.7	10.3
Sample 3-3	In DE	100	14.8	0.0	58.3
	In WM	100	137.9	1037.9	54.5

(d) 30 s UV irradiation on 5 wt% BCSD microcapsules

		DMF	DD	cPPA	Monomer
Peak (ppm)		2.88 to 2.95	0.85 to 0.90	6.25 to 7.1	10.55
The number of protons		6	6	2	2
			I.V.		
Sample 1-1	In DE	100	16.5	0.0	17.8
	In WM	100	51.1	85.3	2.6
Sample 1-2	In DE	100	47.7	0.0	49.2
	In WM	100	44.8	103.0	6.5
Sample 1-3	In DE	100	40.5	0.0	41.9
	In WM	100	85.9	146.2	8.0
Sample 2-1	In DE	100	6.2	0.0	23.4
	In WM	100	11.7	78.3	4.6

Sample 2-2	In DE	100	48.9	0.0	114.4
	In WM	100	154.5	853.7	34.0
Sample 2-3	In DE	100	39.2	0.0	163.8
	In WM	100	142.3	675.5	57.3
Sample 3-1	In DE	100	4.0	0.0	34.7
(Figure S7)	In WM	100	17.8	134.7	9.9
Sample 3-2	In DE	100	5.4	0.0	38.5
	In WM	100	33.6	342.0	23.8
Sample 3-3	In DE	100	16.1	0.0	166.8
	In WM	100	33.5	585.9	86.8

(e) 60 s UV irradiation on 5 wt% BCSD microcapsules

		DMF	DD	cPPA	Monomer
Peak (ppm)		2.88 to 2.95	0.85 to 0.90	6.25 to 7.1	10.55
The number of protons		6	6	2	2
			I.V.		
Sample 1-1	In DE	100	38.9	0.0	41.7
	In WM	100	34.7	77.3	11.2
Sample 1-2	In DE	100	45.9	0.0	54.4
	In WM	100	27.1	72.7	8.6
Sample 1-3	In DE	100	46.9	0.0	45.0
	In WM	100	57.8	114.4	8.5
Sample 2-1	In DE	100	5.2	0.0	24.8
	In WM	100	8.4	55.2	16.2
Sample 2-2	In DE	100	22.2	48.91	48.91
	In WM	100	130.7	154.53	154.53
Sample 2-3	In DE	100	40.5	0.0	234.7
	In WM	100	49.9	322.2	81.7
Sample 3-1	In DE	100	7.4	0.0	62.2
(Figure S8)	In WM	100	6.8	63.1	4.8
Sample 3-2	In DE	100	9.8	0.0	105.1
	In WM	100	6.2	86.2	17.6
Sample 3-3	In DE	100	16.9	0.0	138.6
	In WM	100	81.1	589.9	147.6

(f) 180 s UV irradiation on 5 wt% BCSD microcapsules

		DMF	DD	cPPA	Monomer
Peak (ppm)		2.88 to 2.95	0.85 to 0.90	6.25 to 7.1	10.55
The number of protons		6	6	2	2
			I.V.		
Sample 1-1	In DE	100	0.0	0.0	57.6
	In WM	100	34.7	34.7	7.7
Sample 1-2	In DE	100	52.5	0.0	57.6
	In WM	100	14.2	34.1	8.6
Sample 1-3	In DE	100	69.6	0.0	78.3
	In WM	100	6.0	13.4	6.7

Sample 2-1	In DE	100	1.5	0.0	26.4
	In WM	100	4.8	22.8	7.9
Sample 2-2	In DE	100	0.0	0.0	219.0
	In WM	100	309.8	309.8	75.5
Sample 2-3	In DE	100	38.5	0.0	276.3
	In WM	100	12.9	140.6	21.9
Sample 3-1	In DE	100	3.2	0.0	55.6
(Figure S9)	In WM	100	1.6	22.2	8.1
Sample 3-2	In DE	100	15.4	0.0	115.2
	In WM	100	0.7	6.9	3.2
Sample 3-3	In DE	100	28.0	0.0	199.4
	In WM	100	44.0	268.4	28.0

(g) 300 s UV irradiation on 5 wt% BCSD microcapsules

		DMF	DD	cPPA	Monomer
Peak (ppm)		2.88 to 2.95	0.85 to 0.90	6.25 to 7.1	10.55
The number of protons		6	6	2	2
			I.V.		
Sample 1-1	In DE	100	50.3	0.0	67.8
	In WM	100	1.2	3.8	3.8
Sample 1-2	In DE	100	18.9	0.0	34.5
	In WM	100	0.2	0.3	0.8
Sample 1-3	In DE	100	22.7	0.0	27.7
	In WM	100	0.6	3.5	1.5
Sample 2-1	In DE	100	3.5	0.0	26.1
	In WM	100	0.2	4.2	1.7
Sample 2-2	In DE	100	49.7	0.0	289.0
	In WM	100	10.9	70.0	38.3
Sample 2-3	In DE	100	49.0	0.0	263.8
	In WM	100	3.6	32.7	11.9
Sample 3-1	In DE	100	17.2	0.0	152.3
(Figure S10)	In WM	100	0.7	10.2	5.0
Sample 3-2	In DE	100	16.1	0.0	106.5
	In WM	100	0.3	5.8	2.4
Sample 3-3	In DE	100	30.0	0.0	298.2
	In WM	100	4.2	47.6	15.7

(h) UV irradiation on No PAG microcapsules (control)

		DMF	DD	cPPA	Monomer
Peak (ppm)		2.88 to 2.95	0.85 to 0.90	6.25 to 7.1	10.55
The number of protons		6	6	2	2
			I.V.		
0 s UV	In DE	100	2.2	0.0	0.2
irradiation	In WM	100	123.4	552.8	8.2
60 s UV	In DE	100	2.4	0.0	0.0
irradiation	In WM	100	115.6	469.3	9.6
180 s UV	In DE	100	1.8	0.0	0.0
irradiation	In WM	100	118.4	481.4	4.6

300 s UV	In DE	100	1.3	0.0	0.0
irradiation	In WM	100	112.0	460.1	10.5

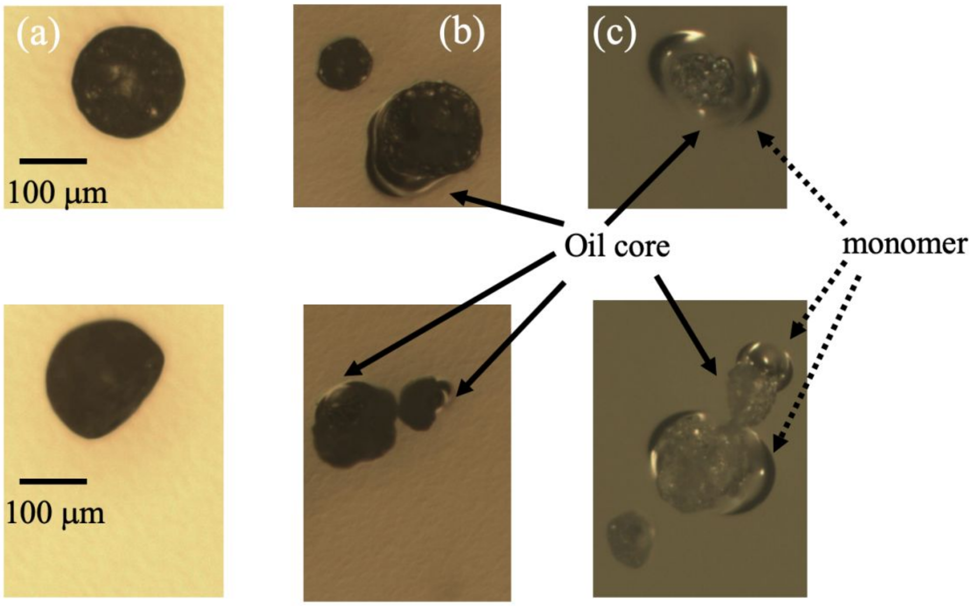

Figure S11. Optical images of cPPA/DD microcapsules under 90 ^{o}C heat treatment are shown. No PAGs are included. The images 24 hr (a), 41 hr (b), and 44 hr (c) after heat treatment, respectively.

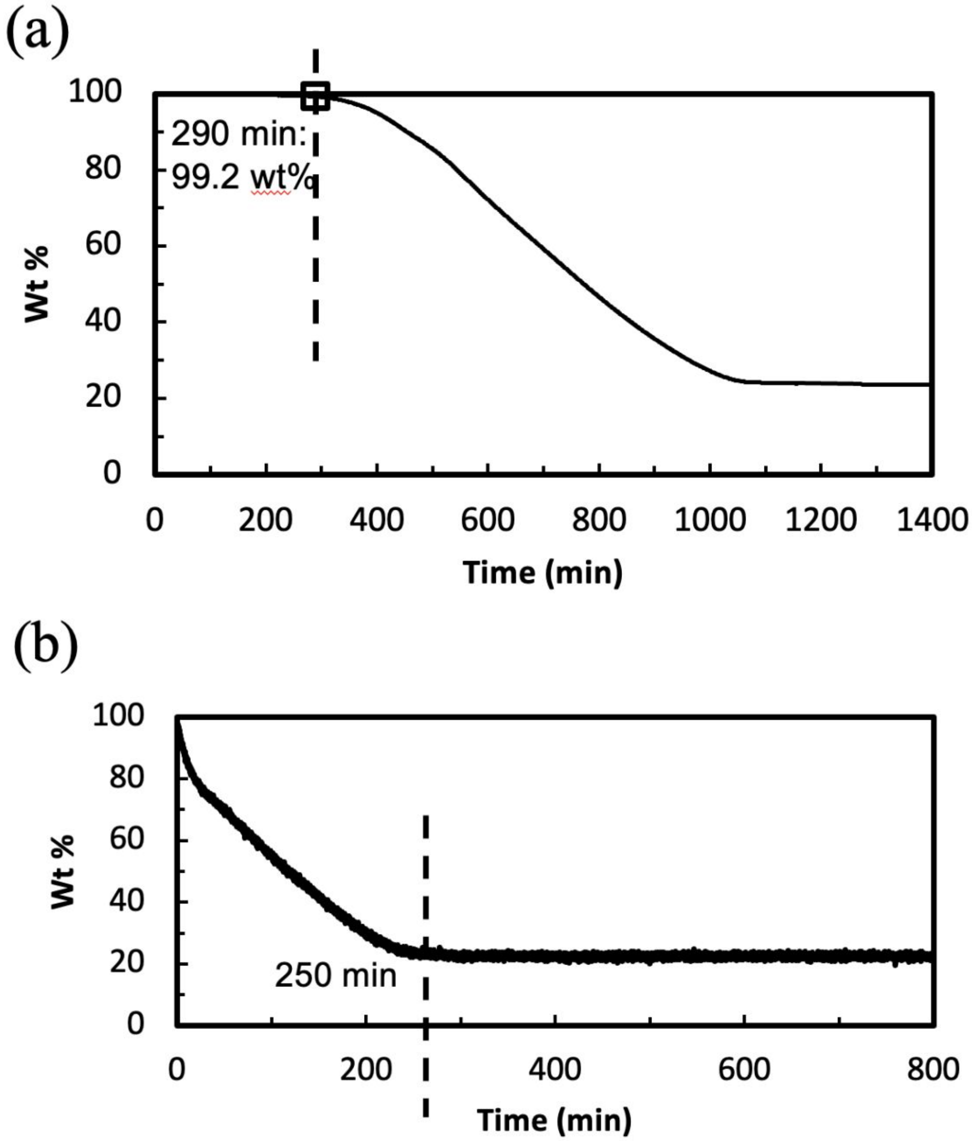

Figure S12. Isothermal thermal gravimetric analysis of cPPA particles (i.e., microcapsules without core) with 20 wt% BCSD at 60 ℃ is shown (a) without UV irradiation and (b) after UV irradiation.

Table S5 -1. cPPA (wt%) data of 20 wt% BCSD microcapsules in Figure 5 (a).

UV (mJ/cm^2)	cPPA weight (mg) before any treatment	cPPA weight (mg) after UV exposure and 250 min heat treatment at 60 °C	cPPA wt%
9	4.800	4.782	99.6
18	6.400	6.097	95.3
36	10.538	10.650	99.0
108	12.895	13.300	97.0
144	8.000	7.265	90.8
180	6.300	1.964	31.2
216	7.700	1.505	19.6
252	7.200	1.636	22.7
288	7.900	1.898	24.0
324	5.300	1.244	23.5
360	8.150	1.702	20.9
396	15.350	2.880	18.8
432	7.900	1.571	19.9
468	10.650	3.011	28.3
756	14.300	3.469	24.3
828	14.350	3.665	25.5
1116	13.700	2.684	19.6
1188	14.700	2.684	18.3
1476	25.750	9.884	38.4
1548	17.150	3.207	18.7
2448	4.000	0.783	19.6
2448	6.200	0.993	16.0
2448	4.700	0.547	11.6
2556	6.800	0.668	9.8
2628	5.400	0.547	10.1
3636	9.000	1.460	16.2
4356	4.900	0.935	19.1

Table S5-2. cPPA (wt%) data of No BCSD microcapsules (control) in Figure 5 (a).

UV (mJ/cm^2)	cPPA weight (mg) before any treatment	cPPA weight (mg) after UV exposure and 250 min heat treatment at 60 °C	cPPA wt%
10800	4.556	4.056	89.0
21600	9.667	8.444	87.4
32400	6.278	5.444	86.7
43200	9.167	8.167	89.1
64800	13.056	11.556	88.5

Table S5-3. cPPA (wt%) data in Figure 5 (b).

	5 wt% BCSD microcapsules	No PAG microcapsule (control)
UV (mJ/cm^2)	cPPA wt (%)	cPPA wt (%)
540	83.5 ± 5.2	-
1080	75.5 ± 7.0	-
2160	59 ± 12	98
6480	32 ± 16	99
10800	9.1 ± 5.2	98

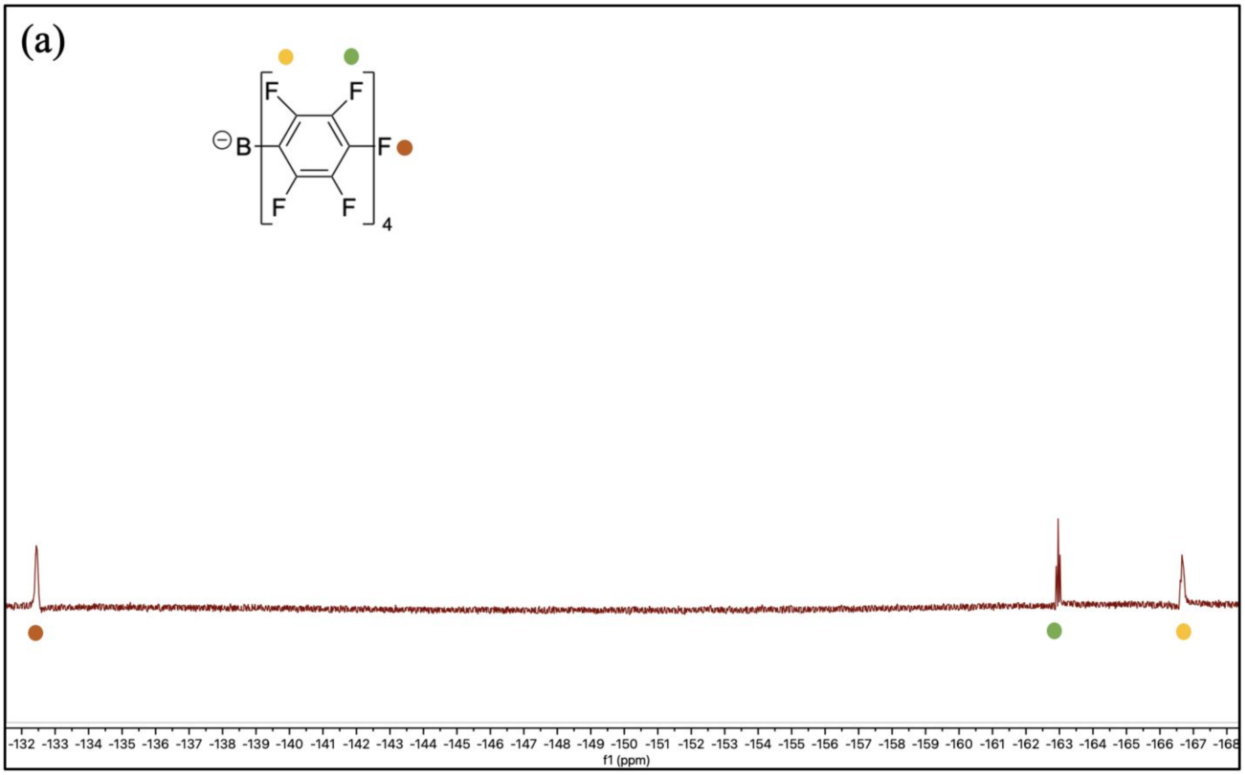
(a)
B
F
F
F
F
F
4
-132 -133 -134 -135 -136 -137 -138 -139 -140 -141 -142 -143 -144 -145 -146 -147 -148 -149 -150 -151 -152 -153 -154 -155 -156 -157 -158 -159 -160 -161 -162 -163 -164 -165 -166 -167 -168
f1 (ppm)

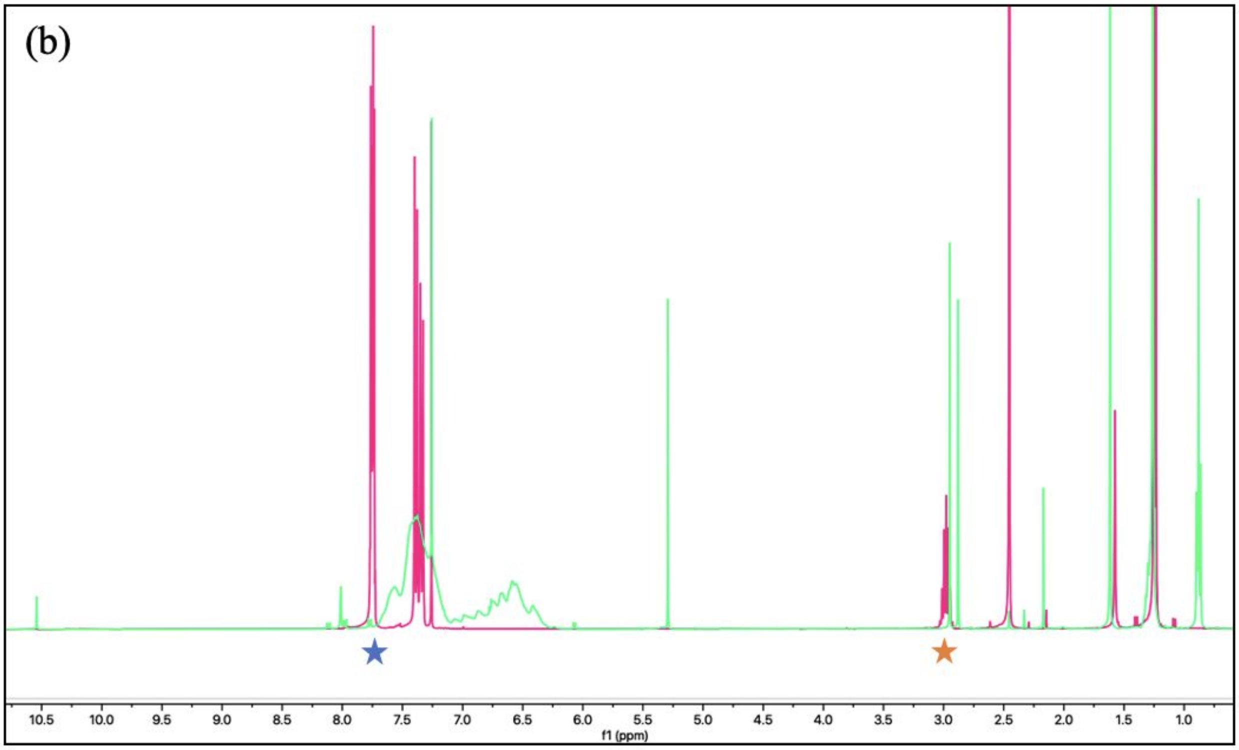
(b)
10.5 10.0 9.5 9.0 8.5 8.0 7.5 7.0 6.5 6.0 5.5 5.0 4.5 4.0 3.5 3.0 2.5 2.0 1.5 1.0
f1 (ppm)

Figure S13. ^1H NMR spectra of 5 wt% Rhodorsil FABA/cPPA/DD are shown: (a) ^{19}F NMR spectrum of the microcapsules and (b) ^1H NMR spectrum of the microcapsule (green) and that of Rhodorsil FABA (pink). Trifluoro-toluene was used as ^{19}F NMR solvent. Two samples in (b) included the same amount of Rhodorsil FABA, representing that the amount of Rhodorsil FABA in the microcapsules is over the limit of detection. In other words, the Rhodorsil FABA peaks should be present in ^1H NMR spectrum of the microcapsules if present. The two distinct Rhodorsil FABA peaks, which are not shown in the ^1H NMR spectrum of the microcapsules, are denoted as stars.

Figure S14 to S18 show ^1H NMR spectra of high MW cPPA/DD microcapsules with 5wt% HNT after diethyl ether wash. The NMR spectra were analyzed to quantify DD release (%) and cPPA depolymerization (%) in the same manner for low MW cPPA/DD/BCSD microcapsules, described in page S-8 to S-9.

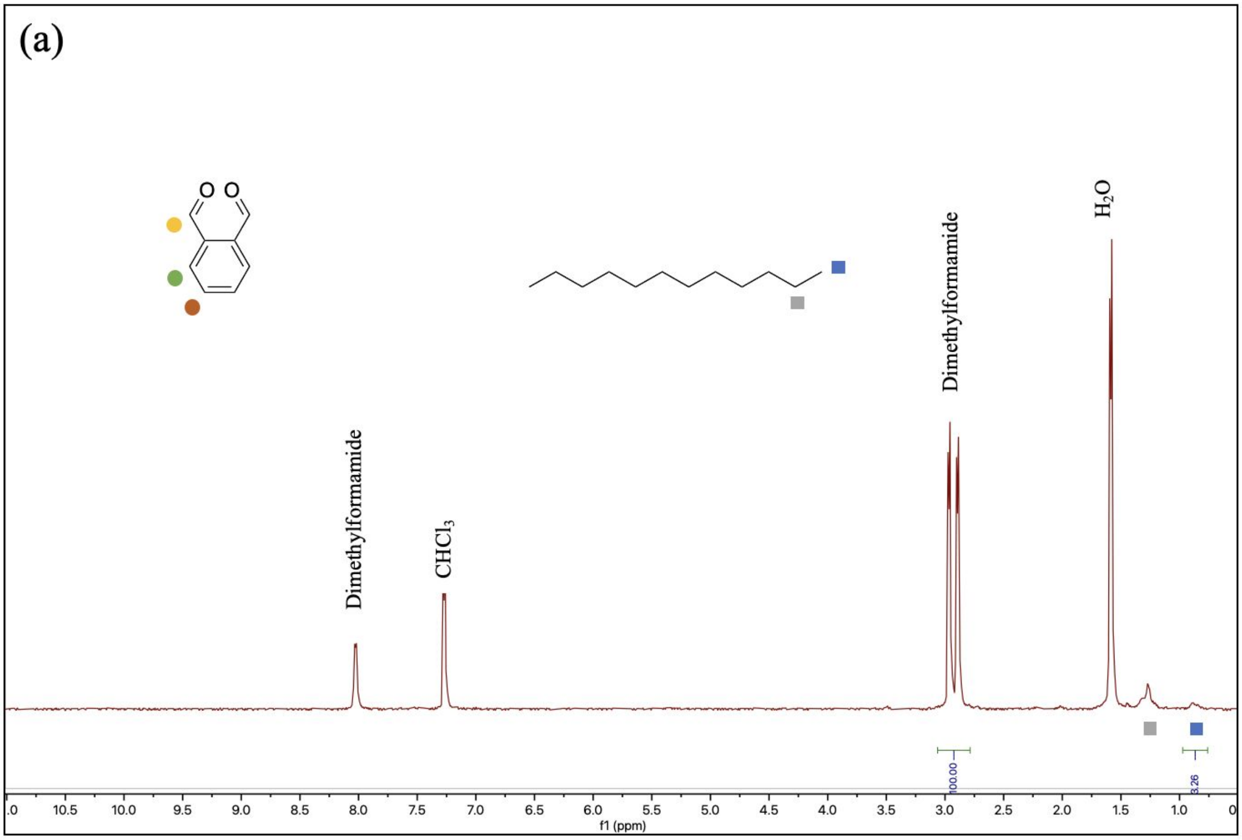

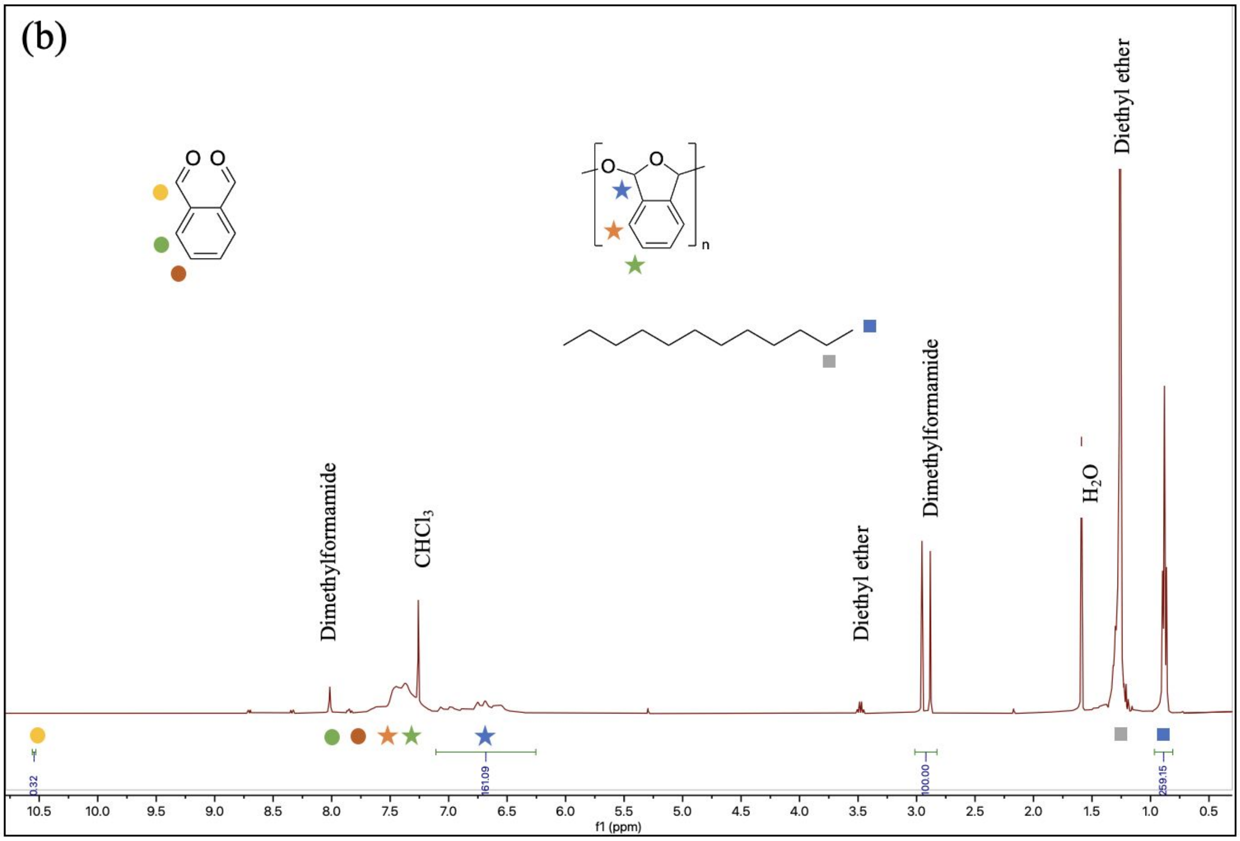

Figure S14. (a) ^{1}H NMR spectrum of washed high MW cPPA/DD/HNT microcapsules shows the residuals from diethyl ether extraction after 0 min of UV irradiation. (b) ^{1}H NMR spectrum is shown for the remaining microcapsules which were washed with diethyl ether and dissolved in $CDCl_3$.

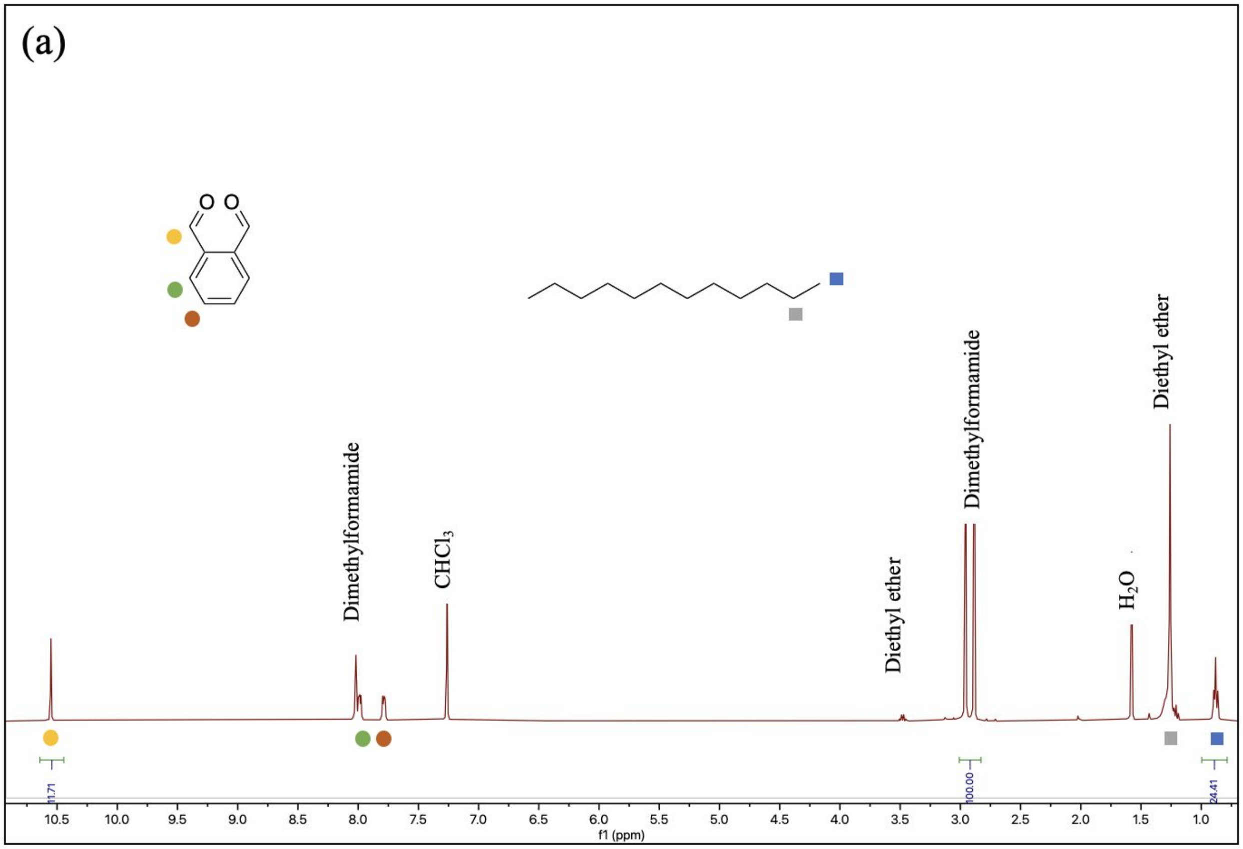
(a)
Dimethylformamide
CHCl3
Diethyl ether
Dimethylformamide
H2O
Diethyl ether
11.71
100.00
24.41
10.5
10.0
9.5
9.0
8.5
8.0
7.5
7.0
6.5
6.0
5.5
5.0
4.5
4.0
3.5
3.0
2.5
2.0
1.5
1.0
f1 (ppm)

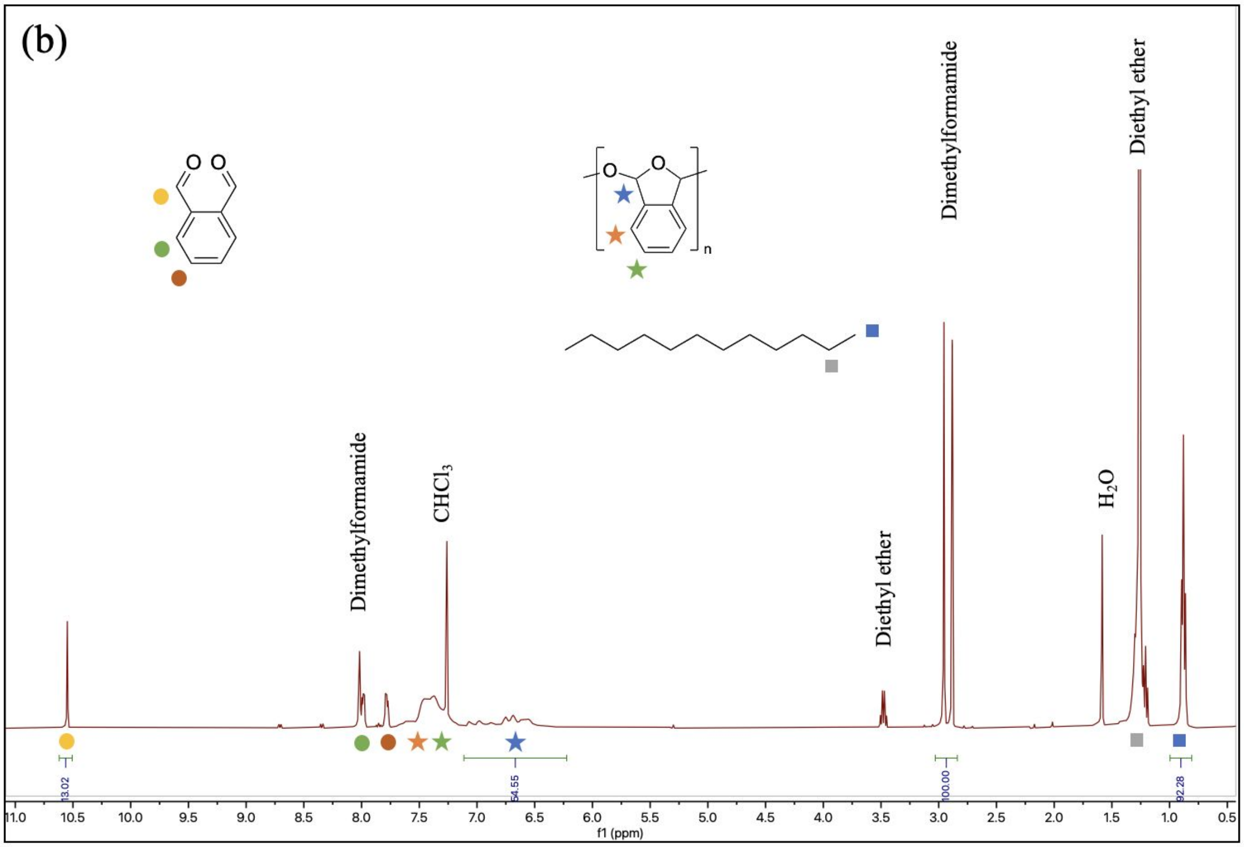

Figure S15. (a) ^{1}H NMR spectrum of washed high MW cPPA/DD/HNT microcapsules shows the residuals from diethyl ether extraction after 1 min of UV irradiation. (b) ^{1}H NMR spectrum is shown for the remaining microcapsules which were washed with diethyl ether and dissolved in $CDCl_3$.

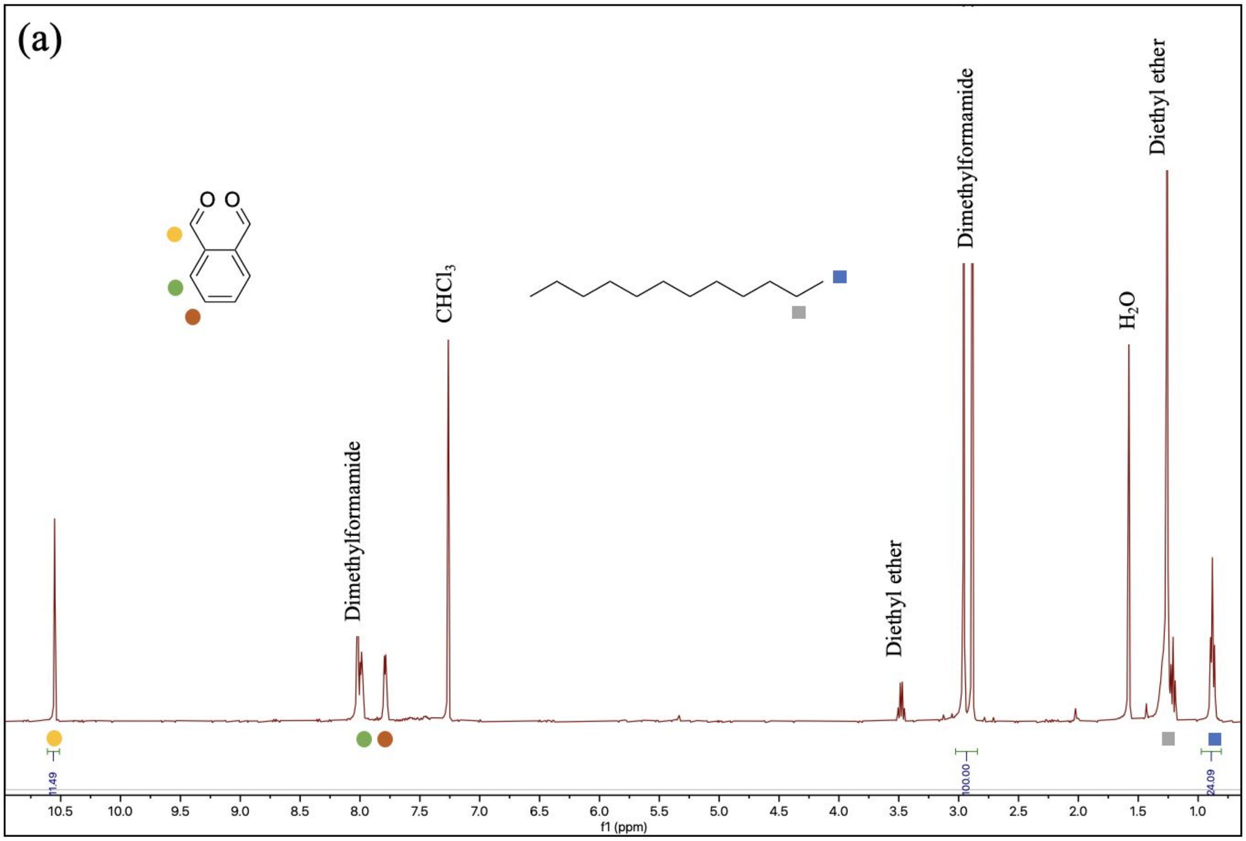

(a)
Dimethylformamide
CHCl3
Diethyl ether
Dimethylformamide
H2O
Diethyl ether
11.49
100.00
24.09
f1 (ppm)

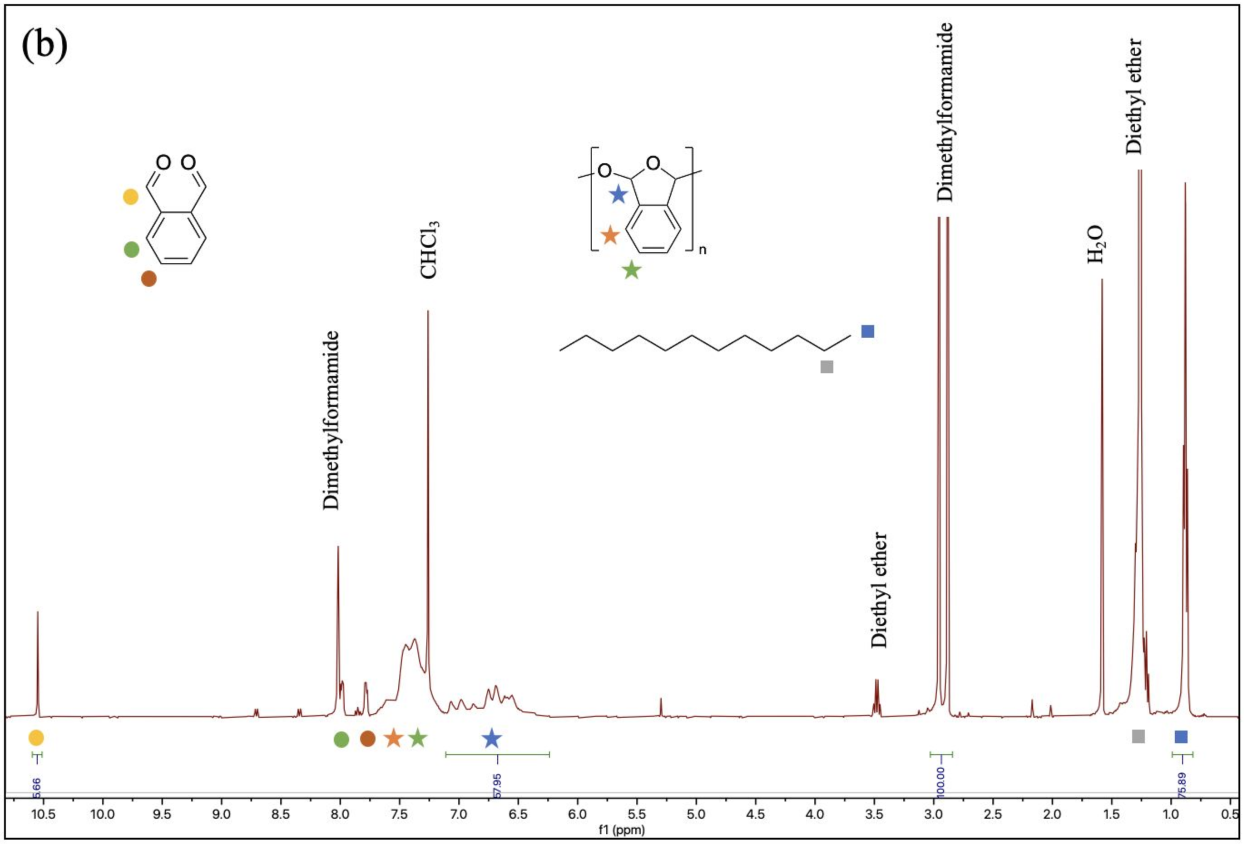

Figure S16. (a) ^{1}H NMR spectrum of washed high MW cPPA/DD/HNT microcapsules shows the residuals from diethyl ether extraction after 3 min of UV irradiation. (b) ^{1}H NMR spectrum is shown for the remaining microcapsules which were washed with diethyl ether and dissolved in $CDCl_3$.

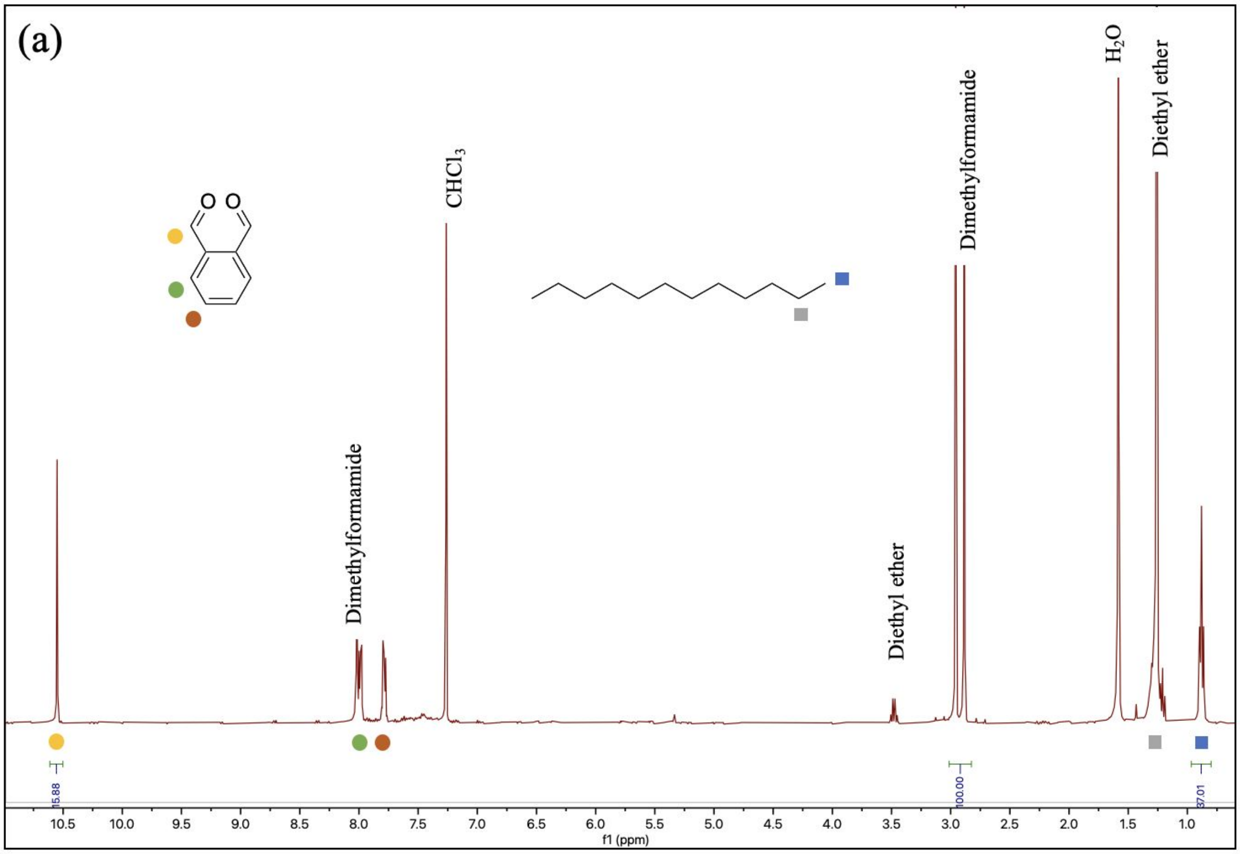
(a)
CHCl3
Dimethylformamide
Diethyl ether
H2O
15.88
100.00
37.01
10.5 10.0 9.5 9.0 8.5 8.0 7.5 7.0 6.5 6.0 5.5 5.0 4.5 4.0 3.5 3.0 2.5 2.0 1.5 1.0
f1 (ppm)

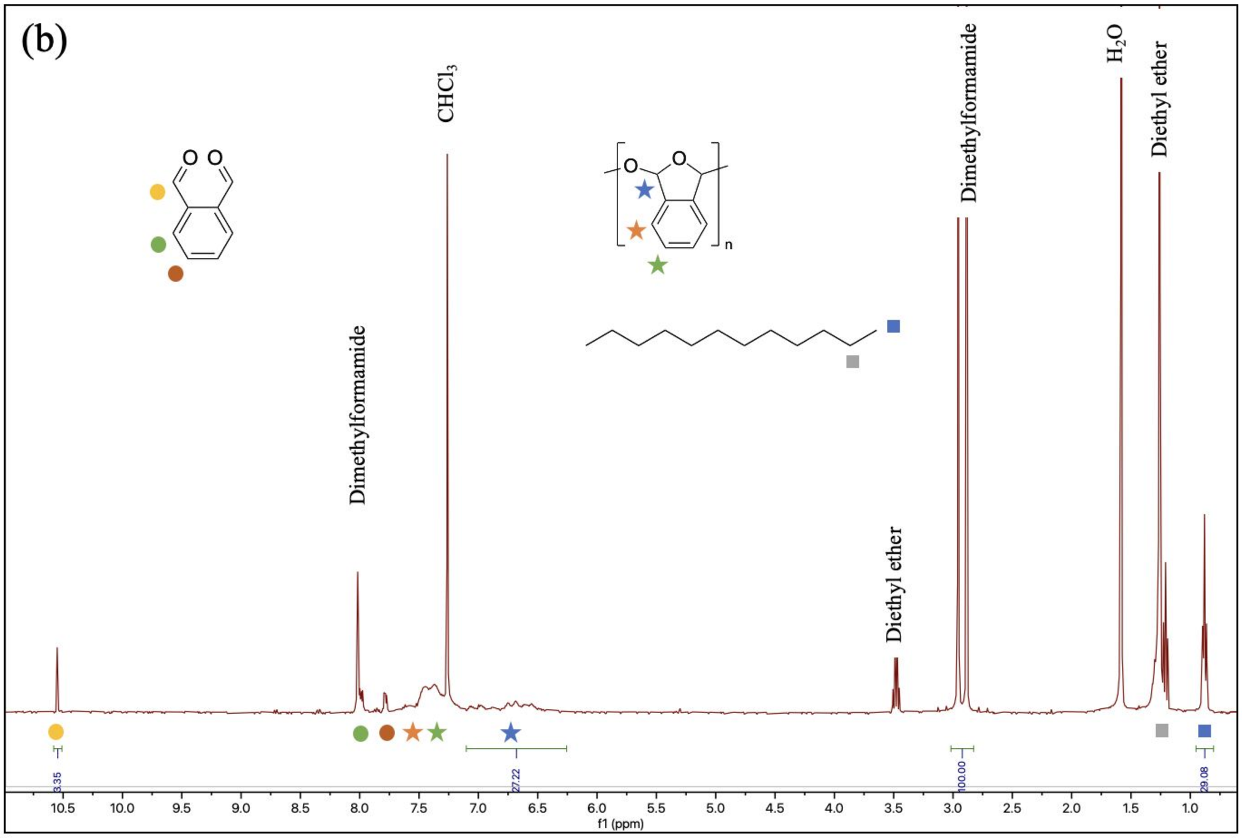

Figure S17. (a) ^{1}H NMR spectrum of washed high MW cPPA/DD/HNT microcapsules shows the residuals from diethyl ether extraction after 5 min of UV irradiation. (b) ^{1}H NMR spectrum is shown for the remaining microcapsules which were washed with diethyl ether and dissolved in $CDCl_3$.

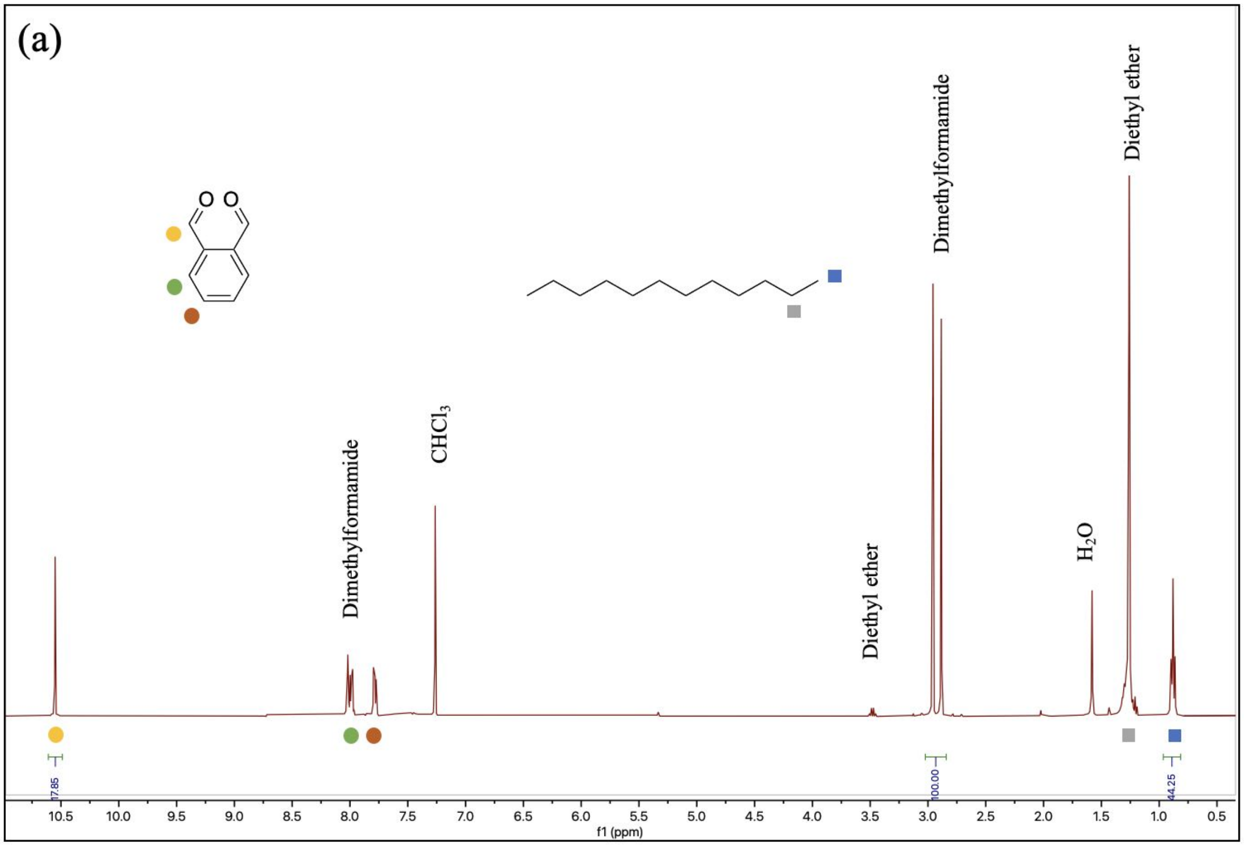
(a)
Dimethylformamide
Diethyl ether
CHCl3
Dimethylformamide
Diethyl ether
H2O
17.85
100.00
44.25
f1 (ppm)

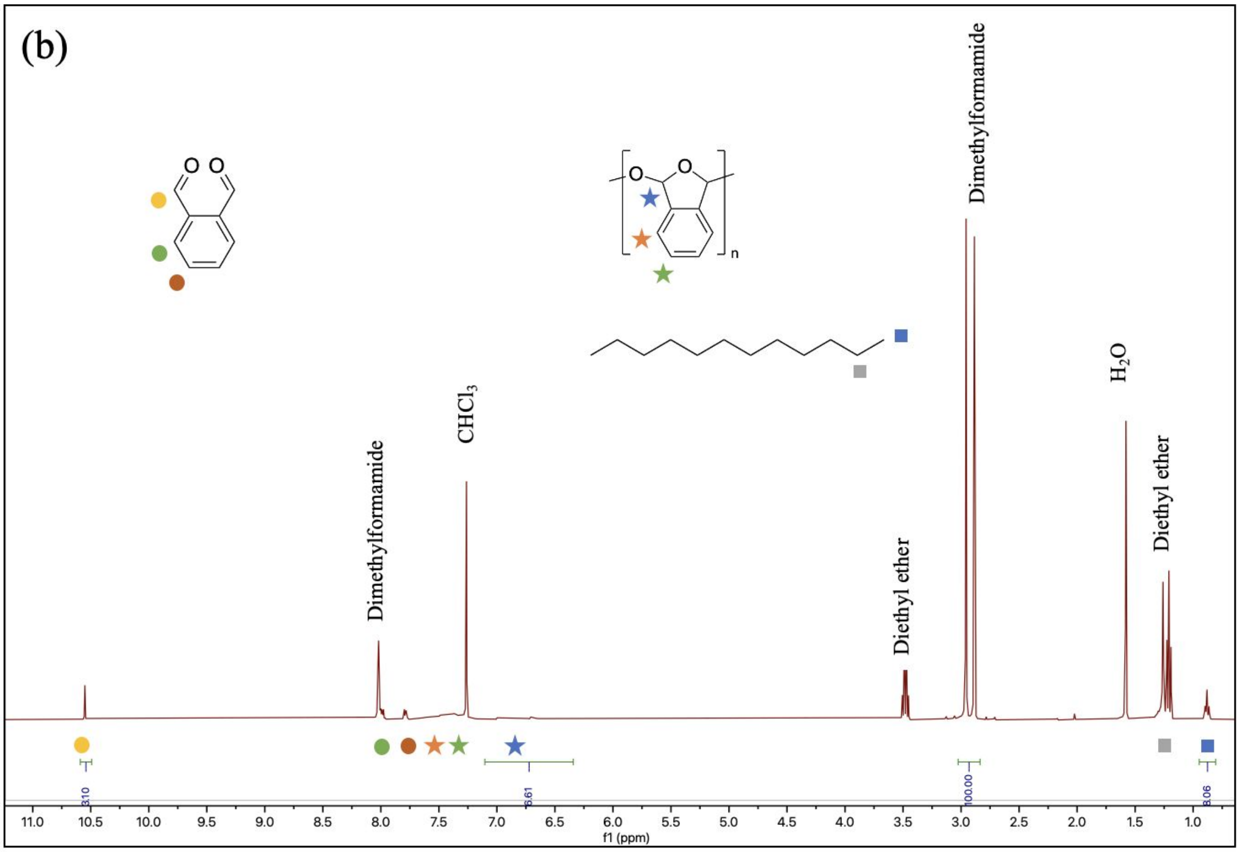
(b)
n
Dimethylformamide
CHCl3
Diethyl ether
Dimethylformamide
H2O
Diethyl ether
3.10
6.61
100.00
8.06
f1 (ppm)

Figure S18. (a) ^{1}H NMR spectrum of washed high MW cPPA/DD/HNT microcapsules shows the residuals from diethyl ether extraction after 7 min of UV irradiation. (b) ^{1}H NMR spectrum is shown for the remaining microcapsules which were washed with diethyl ether and dissolved in $CDCl_3$.

Table S6. (a) The core (DD) release and cPPA depolymerization values from Figure 6 are present. (b) – (g) Integration values (I.V.s) to calculate DD release and cPPA depolymerization values after UV irradiation are tabulated. According to Table S4 and Figure 4, different batches of microcapsules do not differ much. So, only one batch of microcapsules were used for Figure 6.

(a) Numerical values of Figure 6

	5 wt% HNT microcapsules		No PAG microcapsule (control)	
UV irradiation	DD release (%)	cPPA depolymerization (%)	DD release (%)	cPPA depolymerization (%)
0 min	2.6	0.2	-	-
1 min	14.7 ± 6.1	18 ± 12	-	-
3 min	33.3 ± 9.6	28.0 ± 5.6	1.8	0.0
5 min	66.5 ± 9.4	57 ± 14	5.5	0.0
7 min	90.7 ± 6.1	86 ± 12	2.3	0.0

(b) 0 min UV irradiation on 5 wt% HNT microcapsules

		DMF	DD	cPPA	Monomer
Peak (ppm)		2.88 to 2.95	0.85 to 0.90	6.25 to 7.1	10.55
The number of protons		6	6	2	2
				I.V.	
Sample 1 (Figure S14)	In DE	100	3.3	0.0	0.0
	In WM	100	259.2	161.1	0.3

(c) 1 min UV irradiation on 5 wt% HNT microcapsules

		DMF	DD	cPPA	Monomer
Peak (ppm)		2.88 to 2.95	0.85 to 0.90	6.25 to 7.1	10.55
The number of protons		6	6	2	2
				I.V.	
Sample 1-1 (Figure S15)	In DE	100	24.4	0.0	11.7
	In WM	100	92.3	54.6	13.0
Sample 1-2	In DE	100	13.5	0.0	7.7
	In WM	100	139.4	100.3	2.7
Sample 1-3	In DE	100	20.8	0.0	9.9
	In WM	100	123.7	91.5	4.6

(d) 3 min UV irradiation on 5 wt% HNT microcapsules

		DMF	DD	cPPA	Monomer
Peak (ppm)		2.88 to 2.95	0.85 to 0.90	6.25 to 7.1	10.55
The number of protons		6	6	2	2
		I.V.			
Sample 1-1	In DE	100	24.1	0.0	11.5
(Figure S16)	In WM	100	75.9	58.0	5.7
Sample 1-2	In DE	100	28.3	0.0	14.1
	In WM	100	58.6	46.2	3.1
Sample 1-3	In DE	100	40.3	0.0	16.4
	In WM	100	52.8	44.6	6.6

(e) 5 min UV irradiation on 5 wt% HNT microcapsules

		DMF	DD	cPPA	Monomer
Peak (ppm)		2.88 to 2.95	0.85 to 0.90	6.25 to 7.1	10.55
The number of protons		6	6	2	2
		I.V.			
Sample 1-1	In DE	100	37.0	0.0	15.9
(Figure S17)	In WM	100	29.1	27.2	3.4
Sample 1-2	In DE	100	45.1	0.0	20.7
	In WM	100	15.7	11.2	2.4
Sample 1-3	In DE	100	42.4	0.0	20.9
	In WM	100	18.9	14.7	2.9

(f) 7 min UV irradiation on 5 wt% HNT microcapsules

		DMF	DD	cPPA	Monomer
Peak (ppm)		2.88 to 2.95	0.85 to 0.90	6.25 to 7.1	10.55
The number of protons		6	6	2	2
		I.V.			
Sample 1-1	In DE	100	44.3	0.0	17.9
(Figure S18)	In WM	100	8.1	6.6	3.1
Sample 1-2	In DE	100	53.1	0.0	22.3
	In WM	100	5.5	5.8	2.6
Sample 1-3	In DE	100	45.2	0.0	17.6
	In WM	100	1.5	0.1	1.7

(g) UV irradiation on No PAG microcapsules (control)

		DMF	DD	cPPA	Monomer
Peak (ppm)		2.88 to 2.95	0.85 to 0.90	6.25 to 7.1	10.55
The number of protons		6	6	2	2
		I.V.			
3 min UV	In DE	100	1.7	0.0	0.0
irradiation	In WM	100	94.2	66.2	0.0
5 min UV	In DE	100	3.9	0.0	0.0
irradiation	In WM	100	67.7	46.8	0.0
7 min UV	In DE	100	1.9	0.0	0.0
irradiation	In WM	100	78.4	55.7	0.0

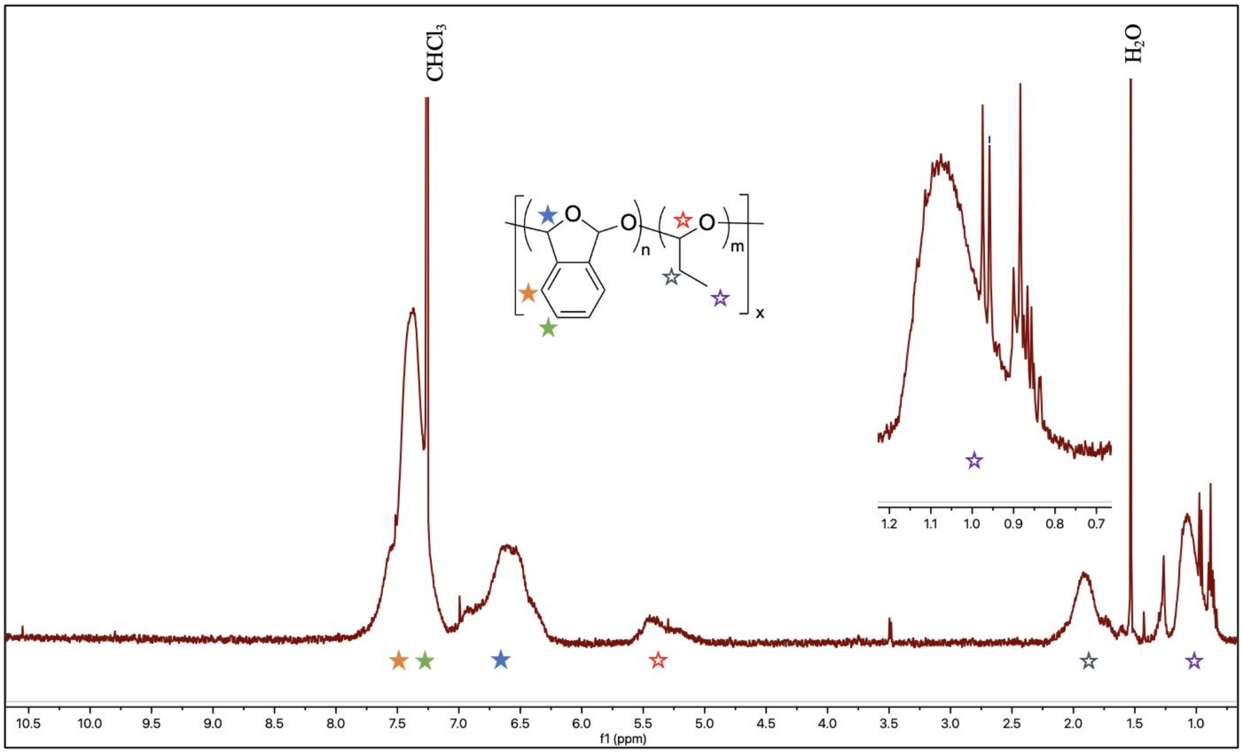

Figure S19. ^{1}H NMR spectrum of copolymer. A broad peak of cPcP (0.82 to 1.18 ppm) is magnified.

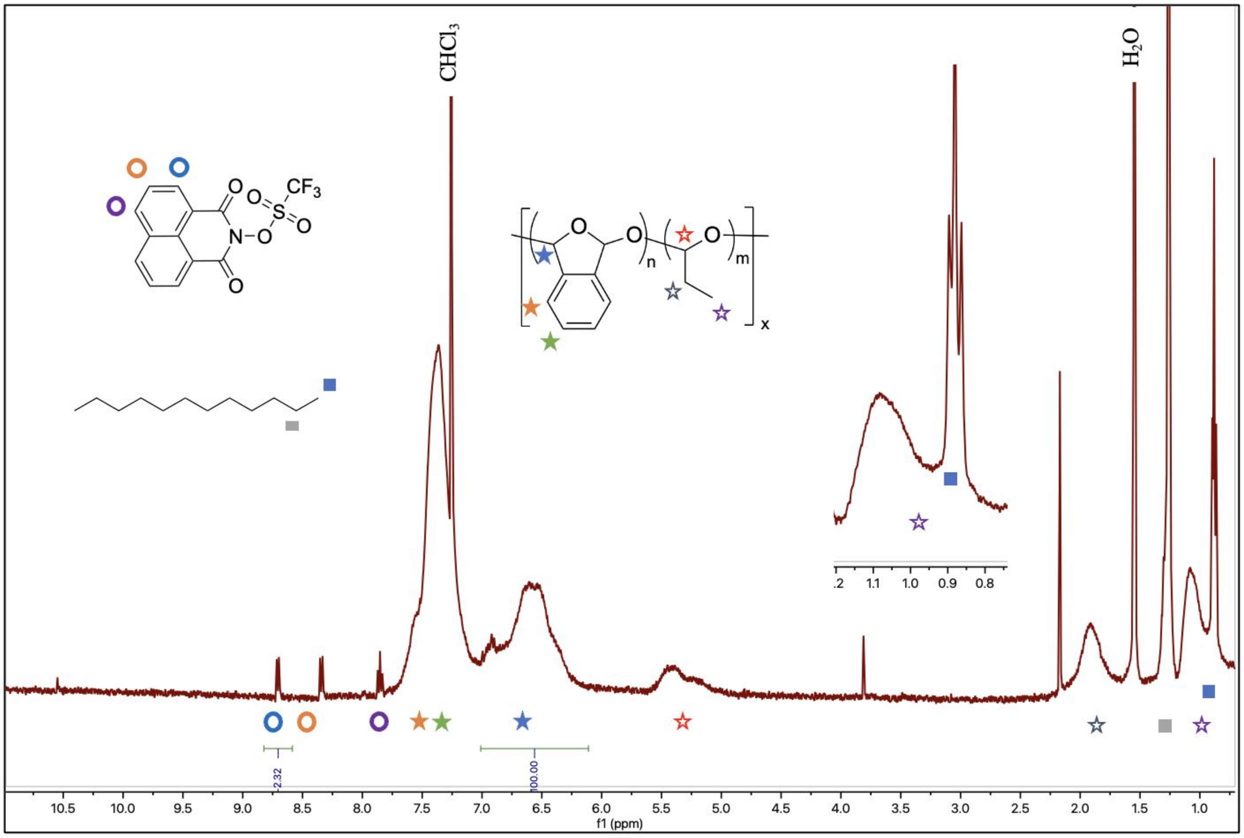

Figure S20. ^{1}H NMR spectrum of copolymer microcapsules with 5 wt% HNT. An overlap of broad peak of cPcP (0.82 to 1.18 ppm) and DD (0.85 to 0.90 ppm) are shown.

Table S7. Integration values (I.V.s) of copolymer and HNT.

	Copolymer	HNT
ppm	6.25 to 7.1	8.7
I.V.	100	2.32
The number of protons	2	2

HNT/cPPA wt% was calculated to be 4.48 %, close to HNT input in the microcapsule fabrication.

Figure S21 to S24 show ^{1}H NMR spectra of copolymer microcapsules after diethyl ether wash. A broad peak of cPcP (0.82 to 1.18 ppm, denoted as purple hollow star) is overlapped with DD (0.85 to 0.90 ppm, denoted as blue square). This region is magnified. The integral values are tabulated in Table S8.

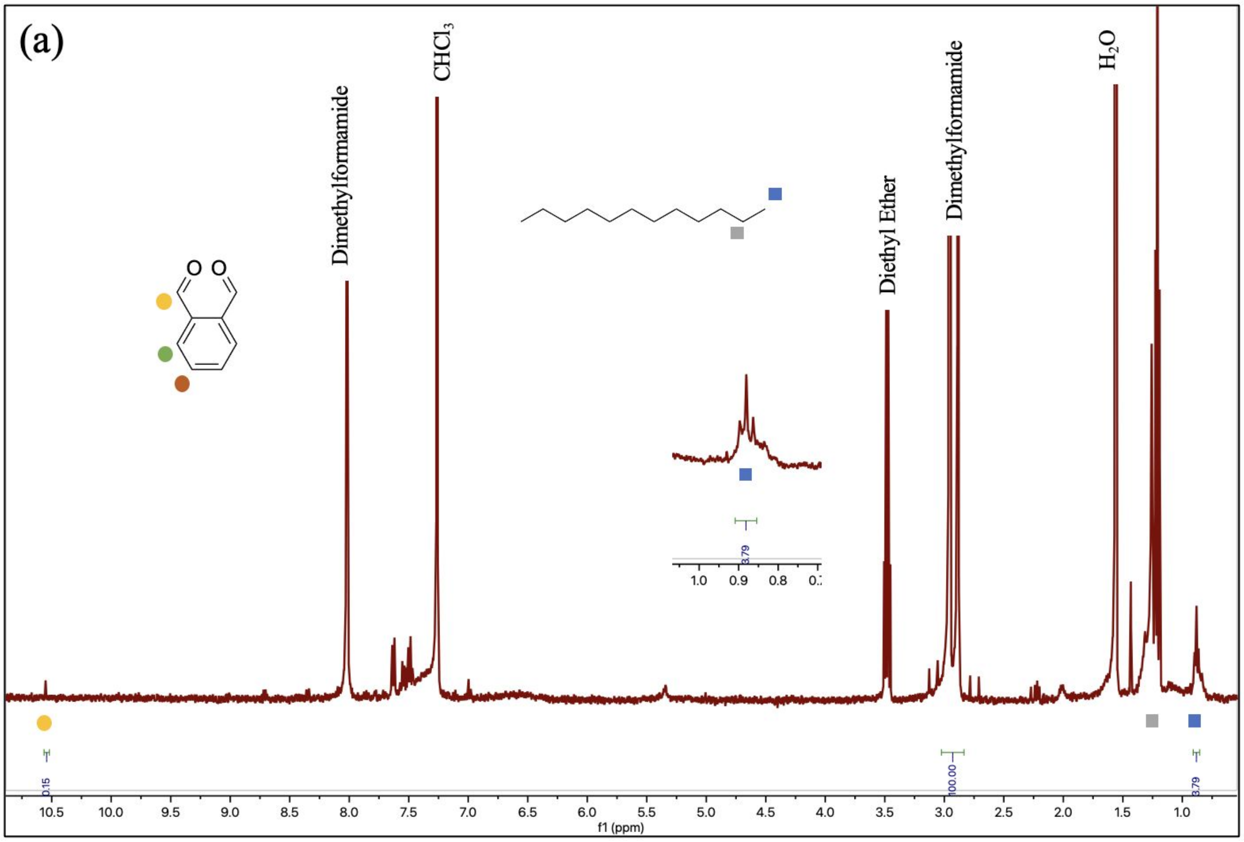
(a)
Dimethylformamide
$CHCl_3$
Diethyl Ether
Dimethylformamide
H_2O
0.15
100.00
3.79
f1 (ppm)

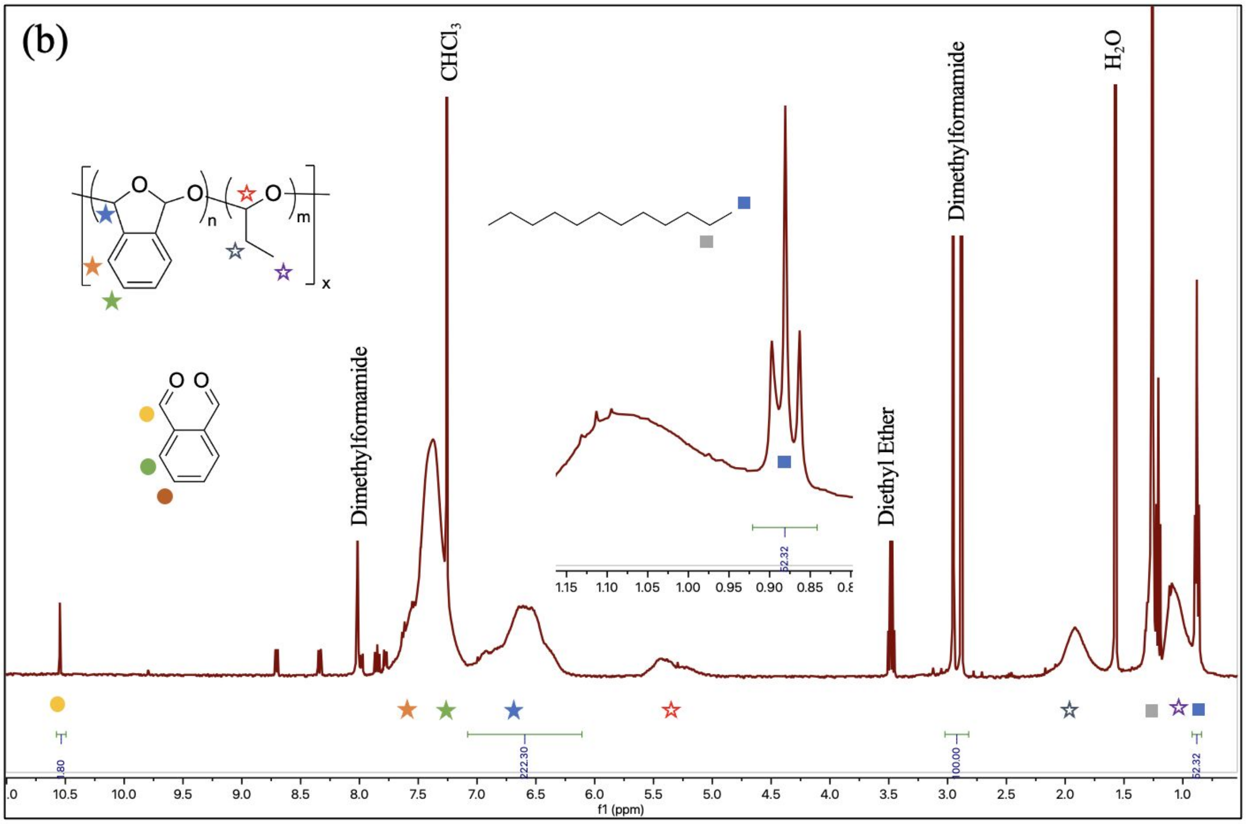
(b)
$CHCl_3$
Dimethylformamide
Dimethylformamide
Diethyl Ether
H_2O
1.80
222.30
100.00
52.32
f1 (ppm)

Figure S21. (a) ^{1}H NMR spectrum of residuals from diethyl ether extraction after 0 s UV irradiation are shown. The copolymer peaks are not shown because it didn't dissolve in diethyl ether. (b) ^{1}H NMR spectrum of remaining microcapsules which were washed with diethyl ether and dissolved in $CDCl_3$ are shown.

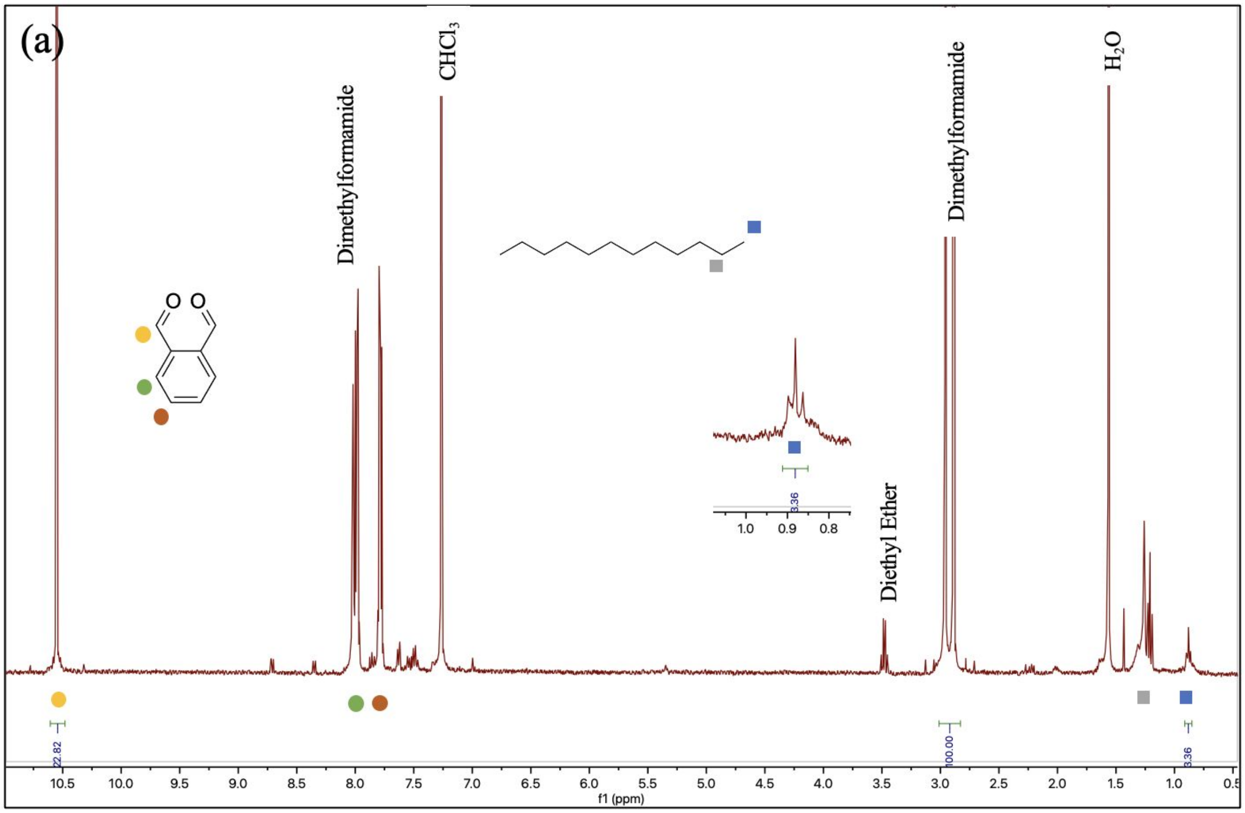

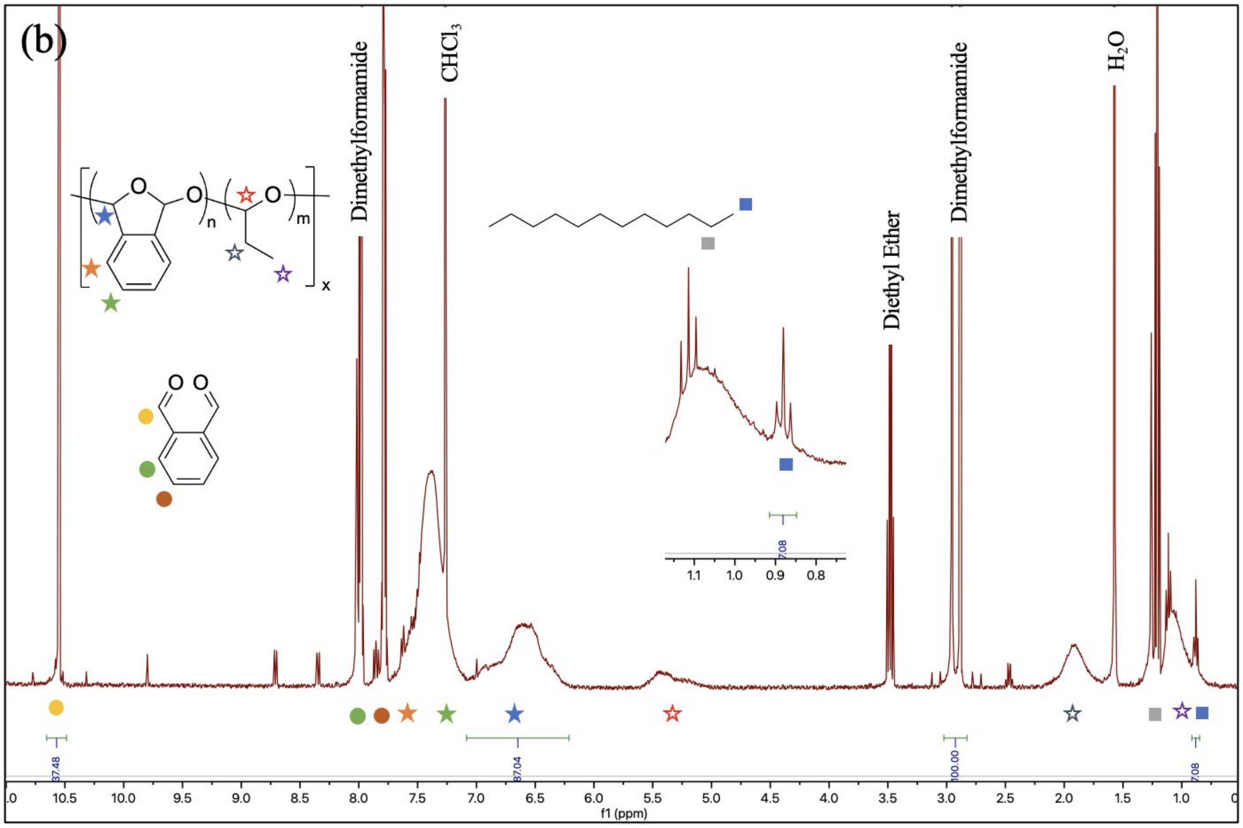

Figure S22. (a) ^{1}H NMR spectrum of residuals from diethyl ether extraction after 10 s UV irradiation are shown. The copolymer peaks are not shown because it didn't dissolve in diethyl ether. (b) ^{1}H NMR spectrum of remaining microcapsules which were washed with diethyl ether and dissolved in $CDCl_3$ are shown.

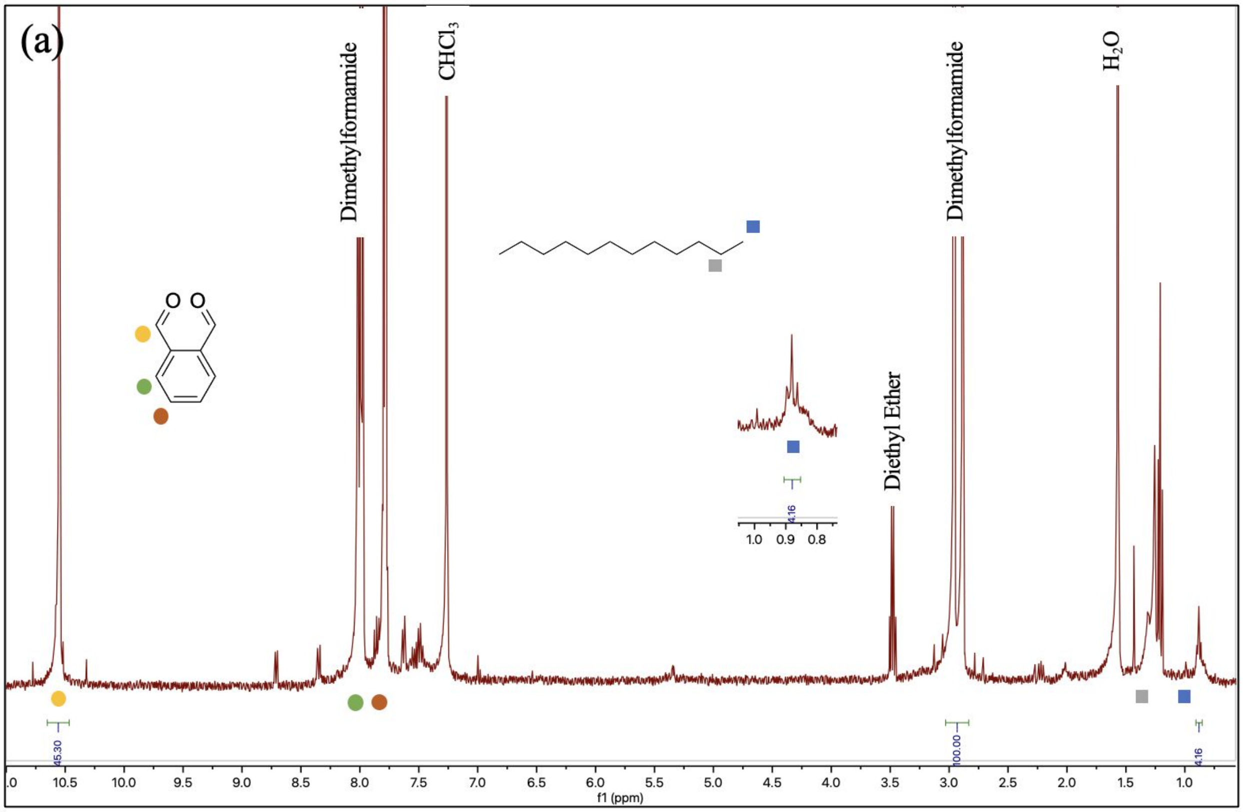
(a)
Dimethylformamide
CHCl3
Dimethylformamide
H2O
Diethyl Ether
O
O
4.16
1.0
0.9
0.8
45.30
100.00
4.16
f1 (ppm)

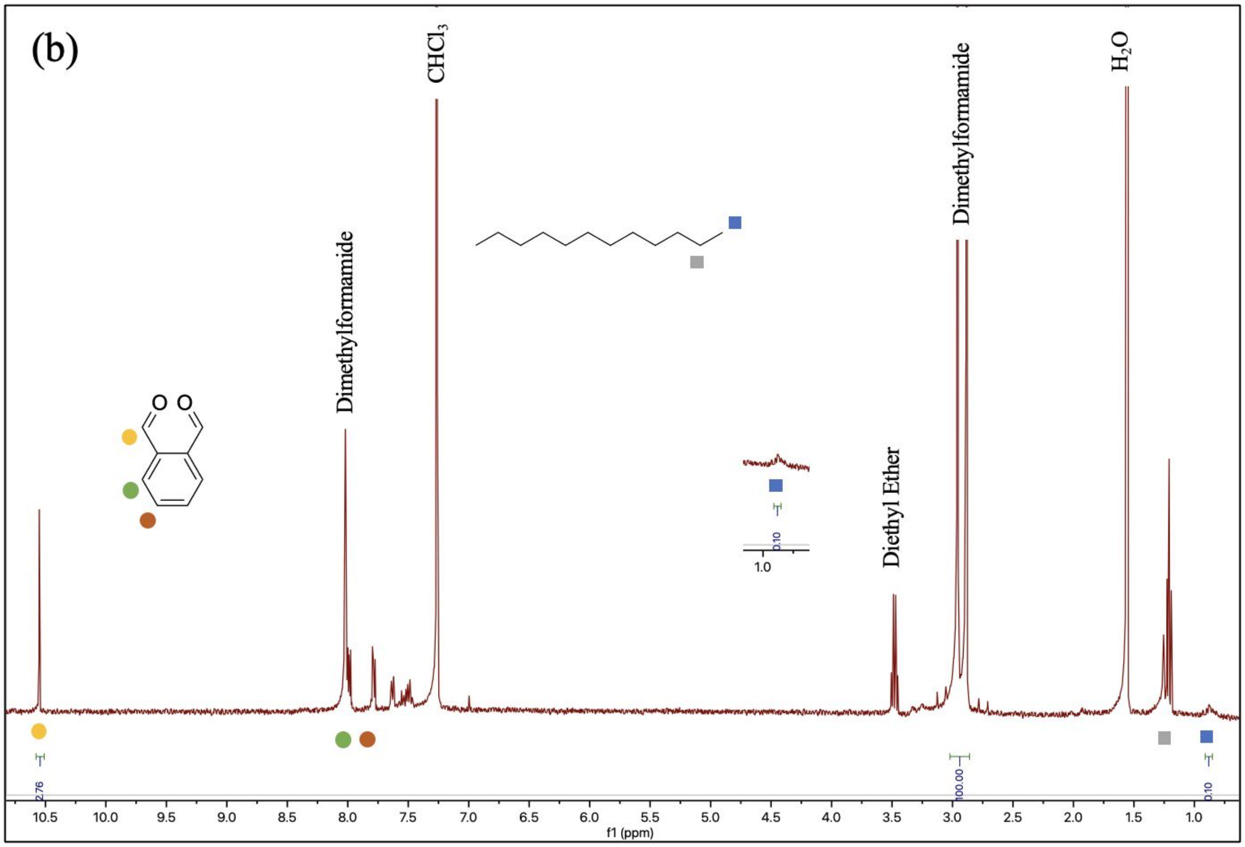
(b)
CHCl3
Dimethylformamide
H2O
Dimethylformamide
Diethyl Ether
O
O
0.10
1.0
2.76
100.00
0.10
f1 (ppm)

Figure S23. (a) ^{1}H NMR spectrum of residuals from diethyl ether extraction after 30 s UV irradiation are shown. The copolymer peaks are not shown because it didn't dissolve in diethyl ether. (b) ^{1}H NMR spectrum of remaining microcapsules which were washed with diethyl ether and dissolved in $CDCl_3$ are shown.

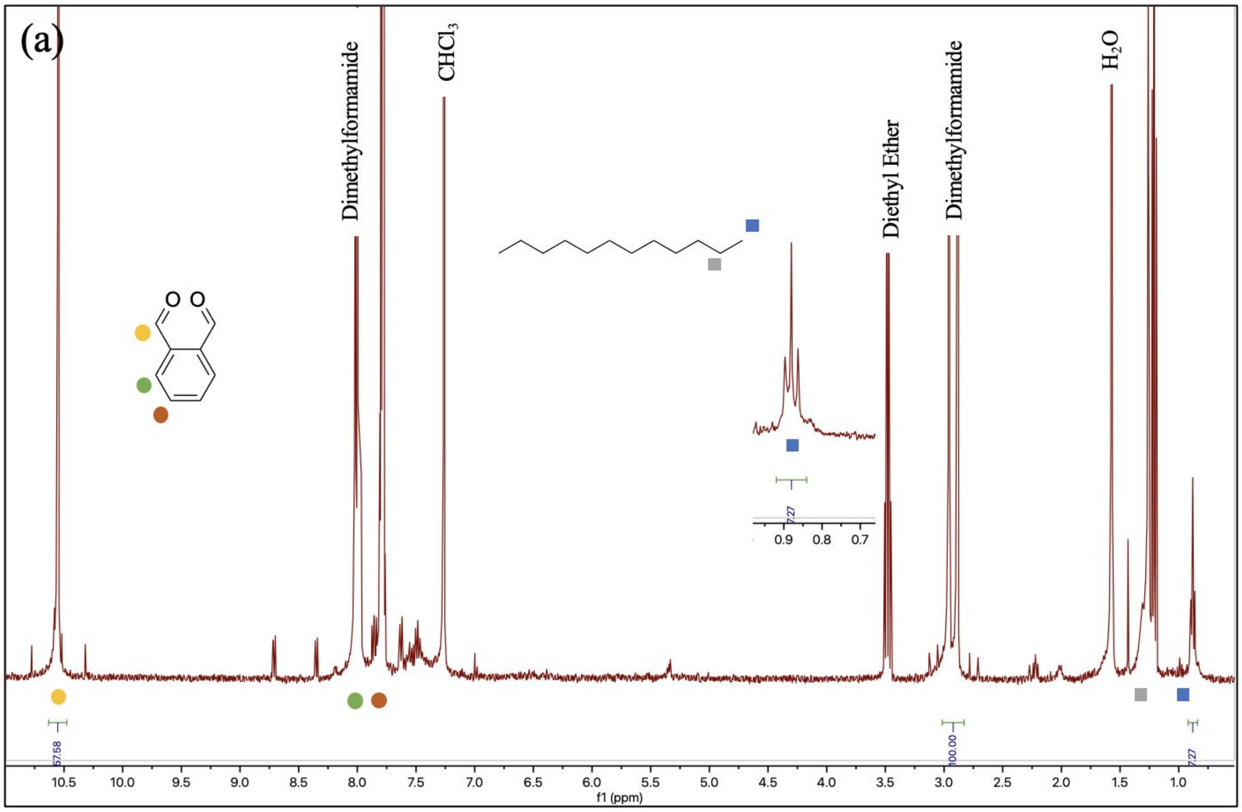

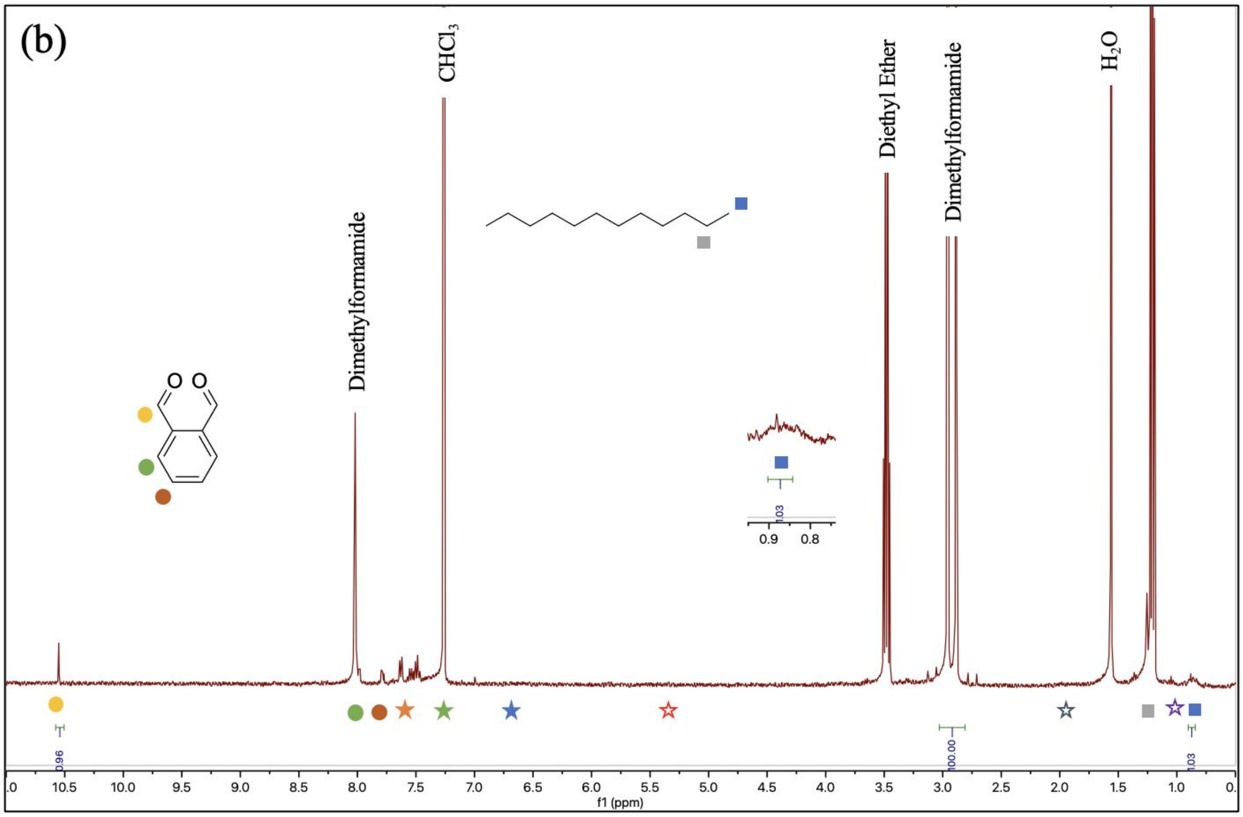

Figure S24. (a) ^{1}H NMR spectrum of residuals from diethyl ether extraction after 60 s UV irradiation are shown. The copolymer peaks are not shown because it didn't dissolve in diethyl ether. (b) ^{1}H NMR spectrum of remaining microcapsules which were washed with diethyl ether and dissolved in $CDCl_3$ are shown.

Table S8. (a) The core (DD) release and copolymer depolymerization values from Figure 7 (c) are present. (b) – (f) Integration values (I.V.s) to calculate DD release (%) and copolymer depolymerization (%) values after UV irradiation are tabulated. According to Table S3 and Figure 4, different batches of microcapsules do not differ much. So, only one batch of microcapsules were used for Figure 7 (c).

(a) Numerical values

	5 wt% HNT microcapsules		No PAG microcapsule (control)	
UV irradiation	DD release (%)	Copolymer depolymerization (%)	DD release (%)	Copolymer depolymerization (%)
0 s	6.5 ± 1.5	1.3 ± 0.5	12.1	0.4
10 s	33.7 ± 5.4	53.0 ± 17.8	-	-
30 s	82.2 ± 13.4	94.8 ± 8.9	-	-
60 s	88.6 ± 1.2	100.0 ± 0.0	13.9	0.2

(b) 0 s UV irradiation on 5 wt% HNT microcapsules

		DMF	DD	Copolymer	Monomer
Peak (ppm)		2.88 to 2.95	0.85 to 0.90	6.25 to 7.1	10.55
The number of protons		6	6	2	2
		I.V.			
Sample 1-1 (Figure S21)	In DE	100	3.8	0	0.15
	In WM	100	52.3	222.3	1.8
Sample 1-2	In DE	100	3.2	0	0.1
	In WM	100	62.3	249.6	0.5
Sample 1-3	In DE	100	6.9	9.2	0.0
	In WM	100	80.8	316.1	4.2

(c) 10 s UV irradiation on 5 wt% HNT microcapsules

		DMF	DD	Copolymer	Monomer
Peak (ppm)		2.88 to 2.95	0.85 to 0.90	6.25 to 7.1	10.55
The number of protons		6	6	2	2
		I.V.			
Sample 1-1 (Figure S22)	In DE	100	3.4	0.0	22.8
	In WM	100	7.1	87.0	37.5
Sample 1-2	In DE	100	3.0	0.0	28.1
	In WM	100	4.6	31.2	58.1
Sample 1-3	In DE	100	2.3	0.0	19.9
	In WM	100	5.6	61.1	29.2

(d) 30 s UV irradiation on 5 wt% HNT microcapsules

		DMF	DD	Copolymer	Monomer
Peak (ppm)		2.88 to 2.95	0.85 to 0.90	6.25 to 7.1	10.55
The number of protons		6	6	2	2
		I.V.			
Sample 1-1 (Figure S23)	In DE	100	4.2	0.0	45.3
	In WM	100	0.1	0.0	2.8
Sample 1-2	In DE	100	9.8	0.0	54.9
	In WM	100	3.4	12.3	12.4
Sample 1-3	In DE	100	2.3	0.0	39.1
	In WM	100	0.8	0.0	0.6

(e) 60 s UV irradiation on 5 wt% HNT microcapsules

		DMF	DD	Copolymer	Monomer
Peak (ppm)		2.88 to 2.95	0.85 to 0.90	6.25 to 7.1	10.55
The number of protons		6	6	2	2
		I.V.			
Sample 1-1 (Figure S24)	In DE	100	7.3	0.0	57.6
	In WM	100	1.0	0.0	1.0
Sample 1-2	In DE	100	1.1	0.0	10.8
	In WM	100	0.1	0.0	0.4
Sample 1-3	In DE	100	1.5	0.0	13.0
	In WM	100	0.2	0.0	0.3

(f) UV irradiation on No PAG microcapsules (control)

		DMF	DD	Copolymer	Monomer
Peak (ppm)		2.88 to 2.95	0.85 to 0.90	6.25 to 7.1	10.55
The number of protons		6	6	2	2
			I.V.		
0 s UV irradiation	In DE	100	2.0	0.0	0.0
	In WM	100	15.8	59.0	0.3
60 s UV irradiation	In DE	100	3.2	0.0	0.0
	In WM	100	20.1	106.4	0.2

The copolymer microcapsules were stored at 3°C for two months, and DD release (%) and copolymer depolymerization were studied. Figures S25 to S28 show ^1H NMR spectra of copolymer microcapsules after diethyl ether wash. A broad peak of cPcP (0.82 to 1.18 ppm, denoted as a purple hollow star) is overlapped with DD (0.85 to 0.90 ppm, denoted as blue square) at low UV dose. This region is magnified. The integral values are tabulated in Table S11.

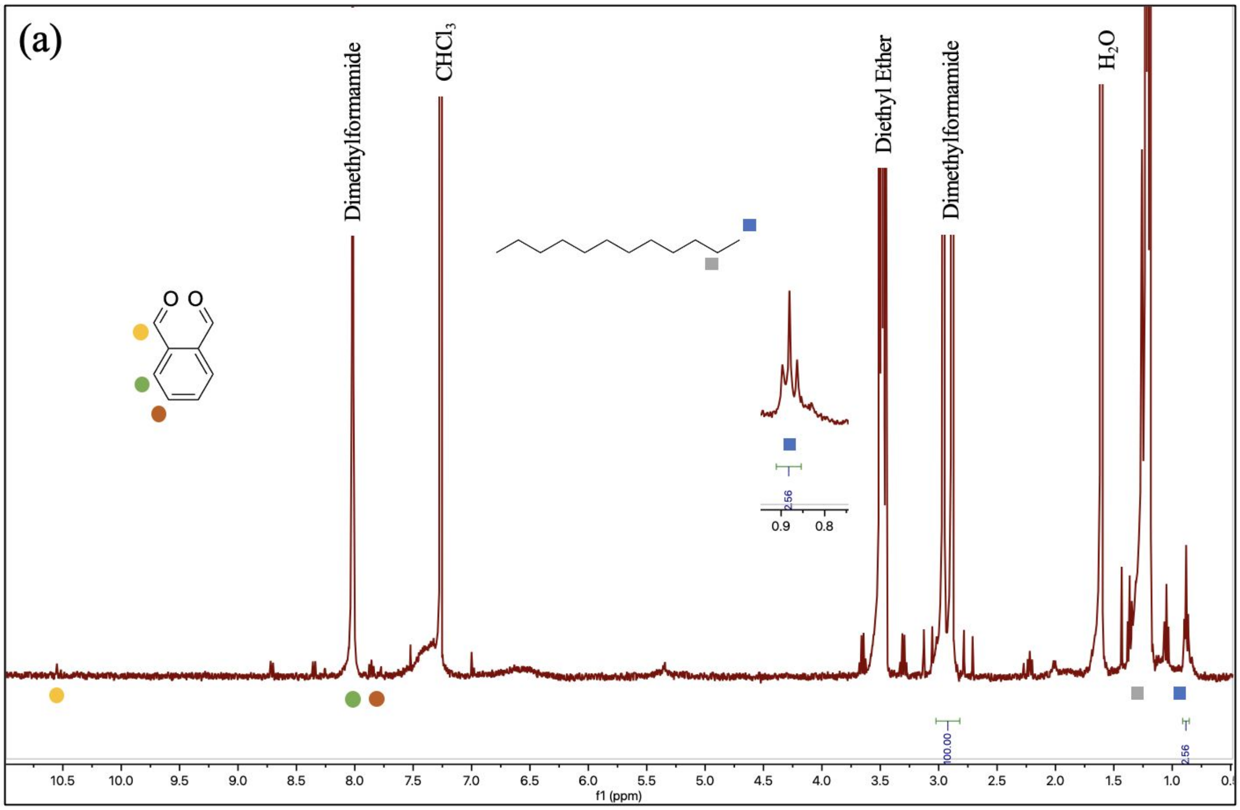

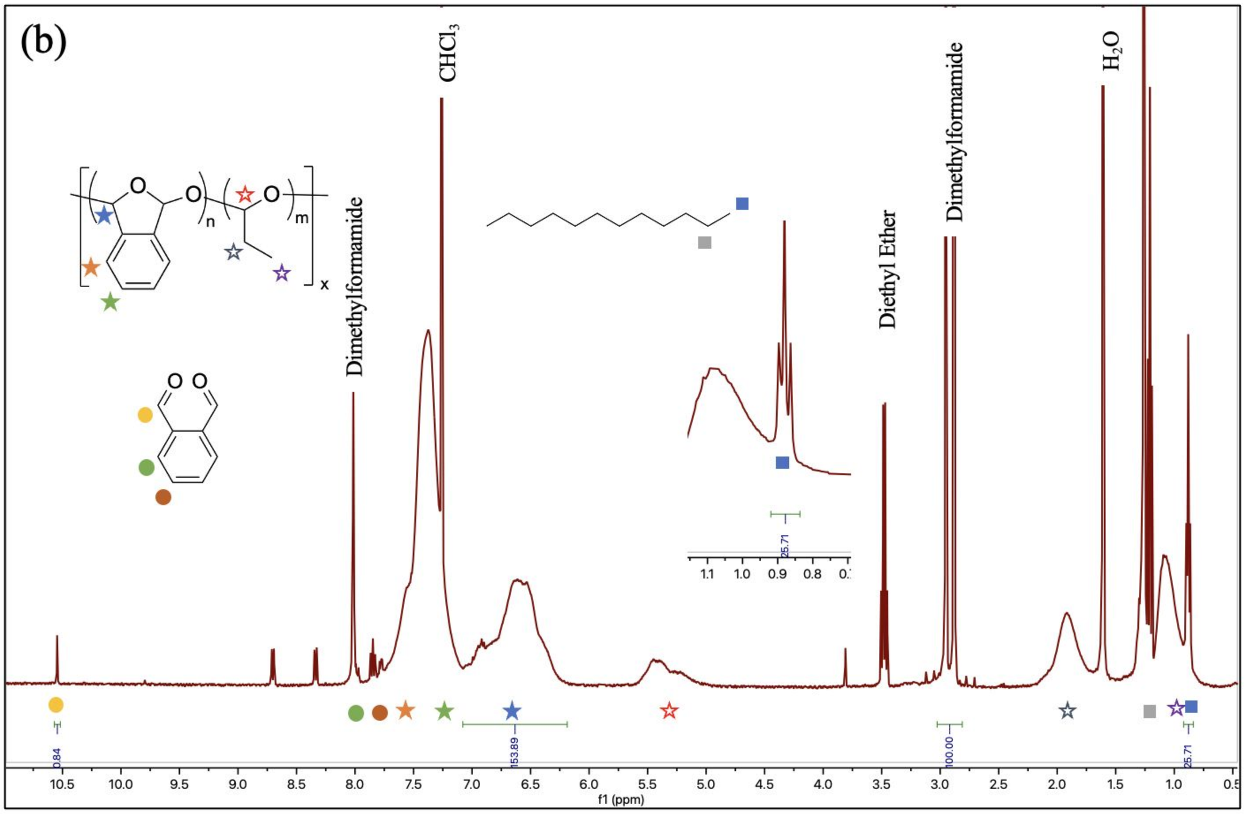

Figure S25. (a) ^{1}H NMR spectrum of residuals from diethyl ether extraction after 0 s UV irradiation are shown. The copolymer peaks are not shown because it didn't dissolve in diethyl ether. (b) ^{1}H NMR spectrum of remaining microcapsules which were washed with diethyl ether and dissolved in $CDCl_3$ are shown.

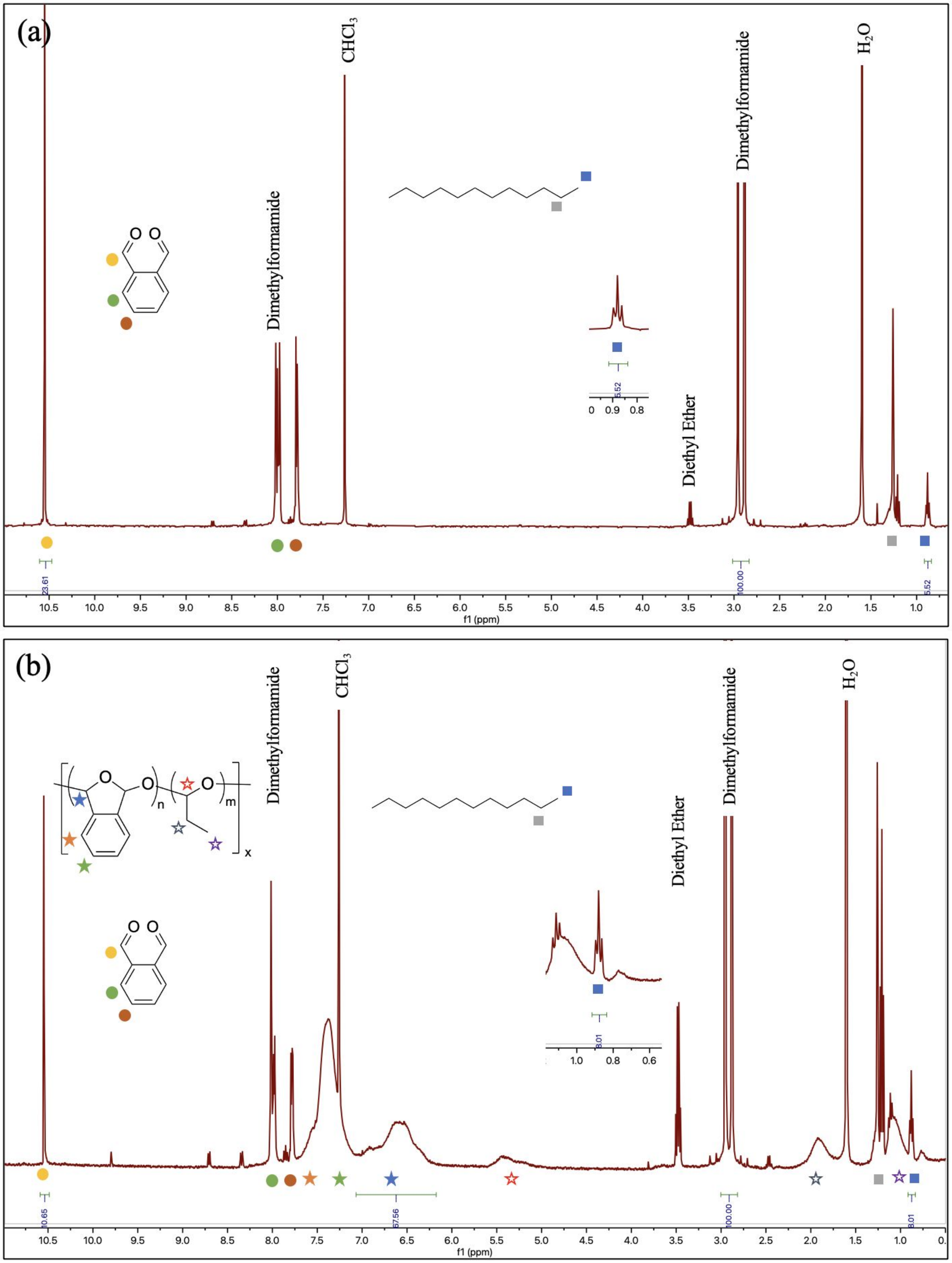
(a)
CHCl$_3$
Dimethylformamide
Dimethylformamide
Diethyl Ether
H$_2$O
23.61
100.00
5.52
10.5 10.0 9.5 9.0 8.5 8.0 7.5 7.0 6.5 6.0 5.5 5.0 4.5 4.0 3.5 3.0 2.5 2.0 1.5 1.0
f1 (ppm)
(b)
CHCl$_3$
Dimethylformamide
Dimethylformamide
Diethyl Ether
H$_2$O
n
m
x
10.65
67.56
100.00
8.01
10.5 10.0 9.5 9.0 8.5 8.0 7.5 7.0 6.5 6.0 5.5 5.0 4.5 4.0 3.5 3.0 2.5 2.0 1.5 1.0
f1 (ppm)

Figure S26. (a) ^{1}H NMR spectrum of residuals from diethyl ether extraction after 10 s UV irradiation are shown. The copolymer peaks are not shown because it did not dissolve in diethyl ether. (b) ^{1}H NMR spectrum of remaining microcapsules which were washed with diethyl ether and dissolved in $CDCl_3$ are shown.

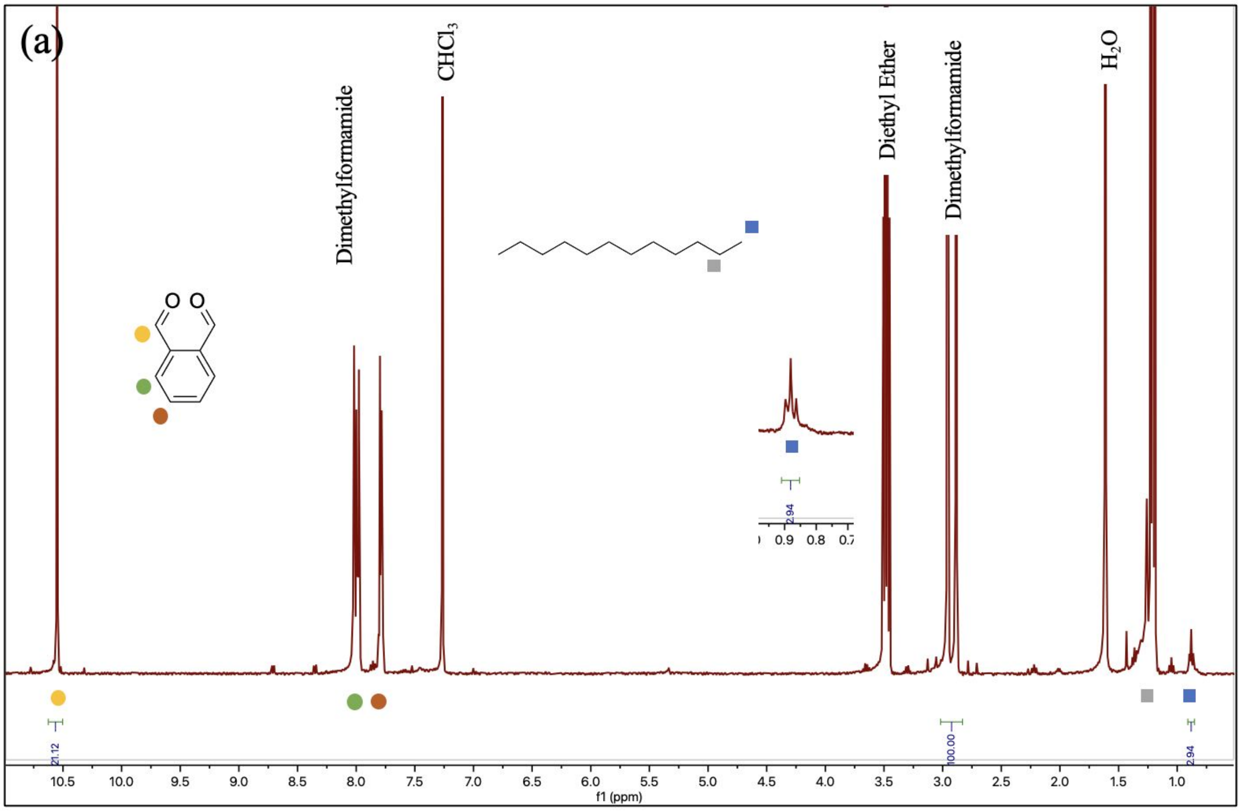

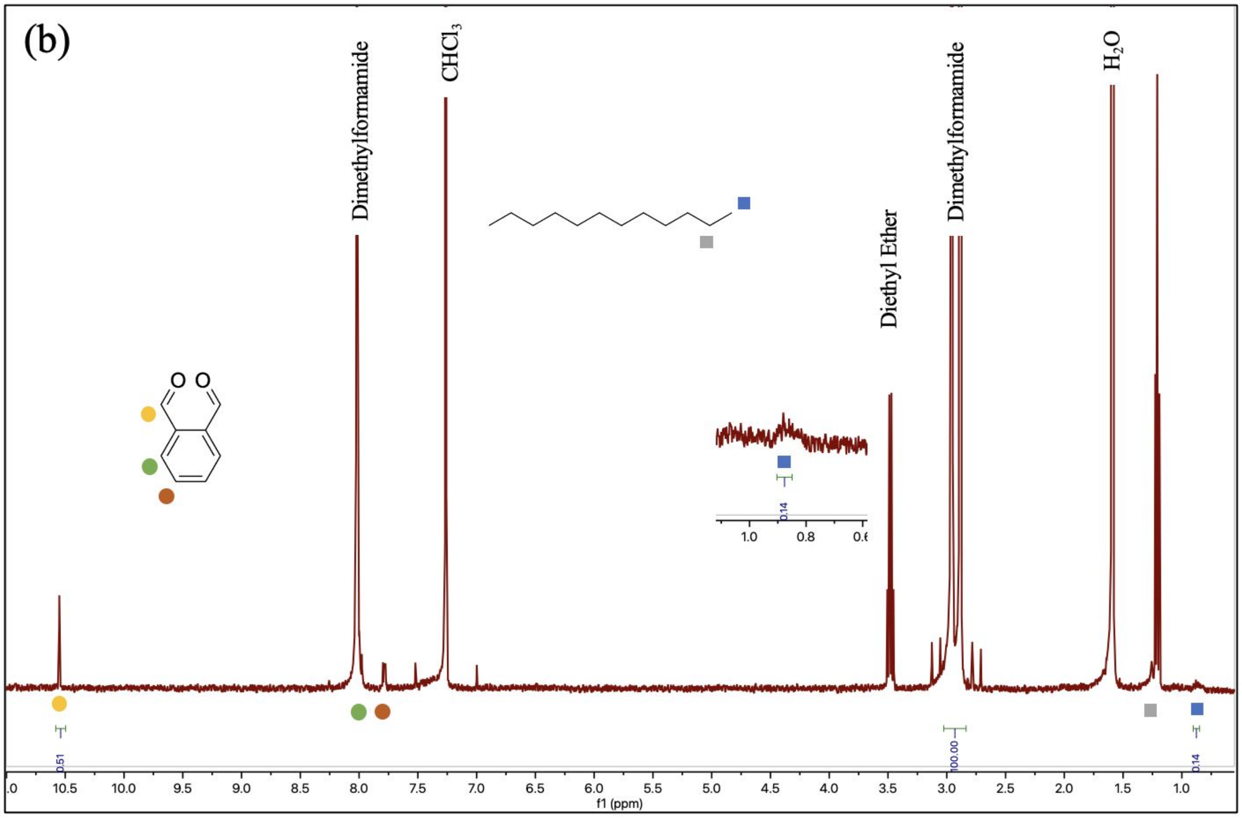

Figure S27. (a) ^{1}H NMR spectrum of residuals from diethyl ether extraction after 30 s UV irradiation are shown. The copolymer peaks are not shown because it did not dissolve in diethyl ether. (b) ^{1}H NMR spectrum of remaining microcapsules which were washed with diethyl ether and dissolved in $CDCl_3$ are shown.

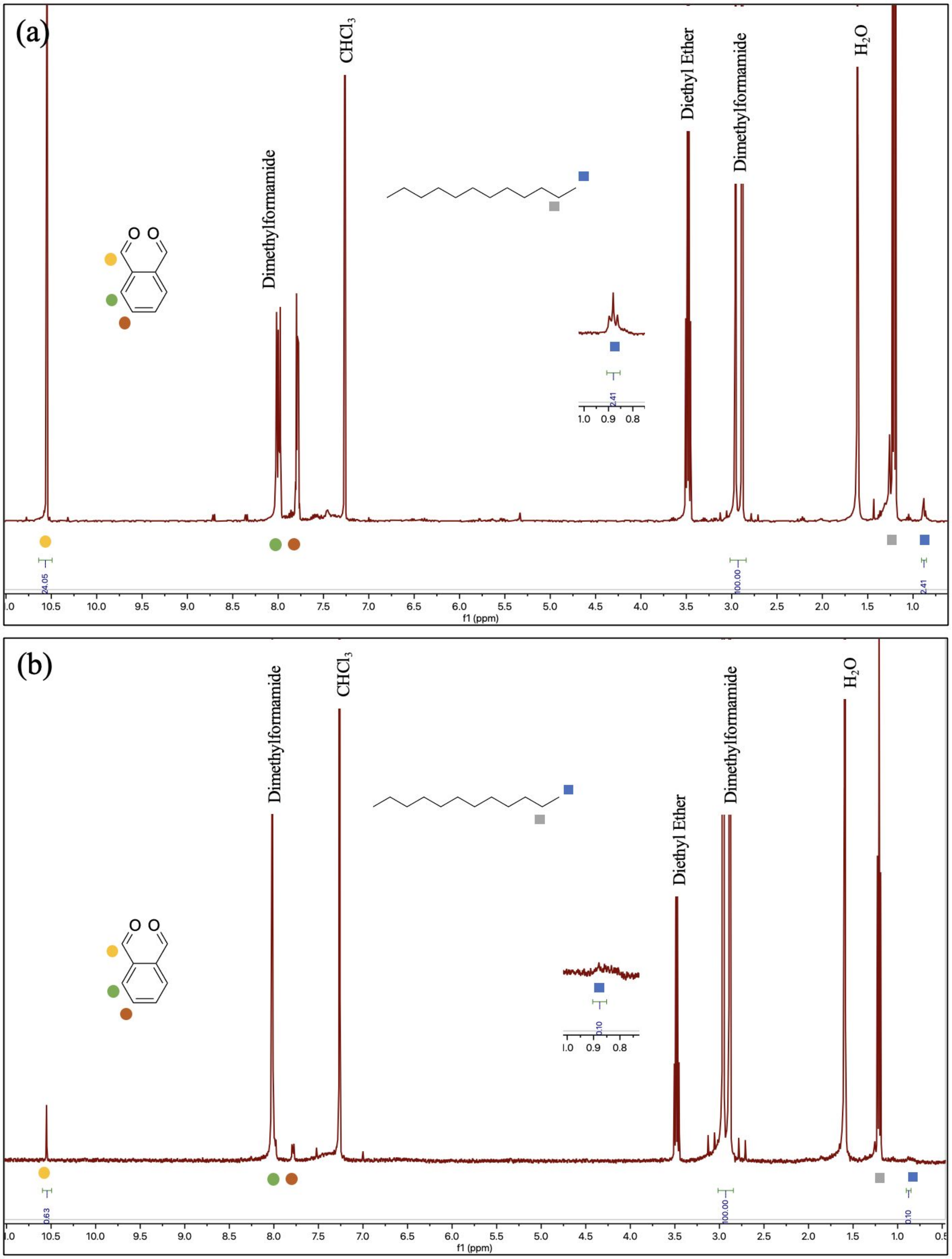

Figure S28. (a) ^{1}H NMR spectrum of residuals from diethyl ether extraction after 60 s UV irradiation are shown. The copolymer peaks are not shown because it did not dissolve in diethyl

ether. (b) ^{1}H NMR spectrum of remaining microcapsules which were washed with diethyl ether and dissolved in $CDCl_3$ are shown.

Table S9. (a) The core (DD) release and copolymer depolymerization values after two months of storage are present (Figure 7 (c)). (b) – (f) Integration values (I.V.s) to calculate DD release and copolymer depolymerization values after UV irradiation are tabulated.

(a) Numerical values

	5 wt% HNT microcapsules after storage	
UV irradiation	DD release (%)	Copolymer depolymerization (%)
0 s	9.1	0.5
10 s	40.8	33.6
30 s	95.5	100
60 s	96.0	99.3

(b) Integral values (I.V.s)

		DMF	DD	Copolymer	Monomer
Peak (ppm)		2.88 to 2.95	0.85 to 0.90	6.25 to 7.1	10.55
The number of protons		6	6	2	2
		I.V.			
0 s UV irradiation (Figure S25)	In DE	100	2.6	0.0	0.0
	In WM	100	25.7	153.9	0.8
10 s UV irradiation (Figure S26)	In DE	100	5.5	0.0	23.6
	In WM	100	8.0	67.6	10.7
30 s UV irradiation (Figure S27)	In DE	100	2.9	0.0	21.1
	In WM	100	0.1	0.0	0.5
60 s UV irradiation (Figure S28)	In DE	100	2.4	0.0	24.1
	In WM	100	0.1	0.0	0.6

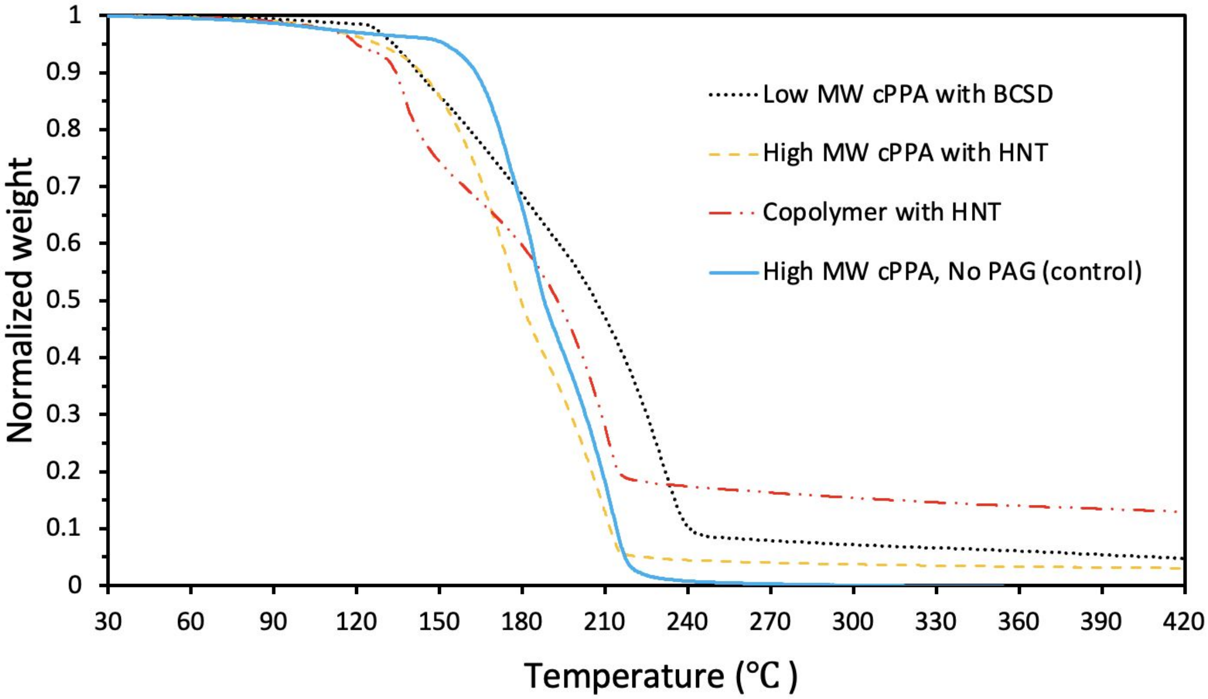

Figure S29. Dynamic thermal gravimetric analysis for the series of microcapsules used in this study. The temperature ramp was 10 °C/min.
